# Jinlida for Diabetes Prevention in Impaired Glucose Tolerance and Multiple Metabolic Abnormalities: The FOCUS Randomized Clinical Trial

**DOI:** 10.1001/jamainternmed.2024.1190

**Published:** 2024-06-03

**Authors:** Hangyu Ji, Xuefei Zhao, Xinyan Chen, Hui Fang, Huailin Gao, Geng Wei, Min Zhang, Hongyu Kuang, Baijing Yang, Xiaojun Cai, Yanjin Su, Chunli Piao, Shuyu Zhao, Liyang Li, Wenliang Sun, Tianshu Xu, Qinghua Xu, Yuan Fan, Jianhua Ye, Chen Yao, Meixia Shang, Guangyao Song, Liming Chen, Qingshan Zheng, Xinhua Xiao, Li Yan, Fengmei Lian, Xiaolin Tong, Zhenhua Jia

**Affiliations:** 1 Good Clinical Practice Office, Guang’anmen Hospital China Academy of Chinese Medical Sciences, Beijing, China; 2 Department of Endocrinology, Guang’anmen Hospital China Academy of Chinese Medical Sciences, Beijing, China; 3 Department of Prevention and Treatment of Disease, Guangdong Provincial Hospital of Traditional Chinese Medicine, Guangzhou, China; 4 Department of Endocrinology, Tangshan Gongren Hospital, Tangshan, China; 5 Department of Endocrinology, Hebei Yiling Hospital, Shijiazhuang, China; 6 Department of Traditional Chinese Medicine, Shijiazhuang 2nd Hospital, Shijiazhuang, China; 7 Department of General Practice, Baotou Central Hospital, Baotou, China; 8 Department of Endocrinology, The First Affiliated Hospital of Harbin Medical University, Harbin, China; 9 Department of Traditional Chinese Medicine, The First Affiliated Hospital of Medical College of Shihezi University, Shihezi, China; 10 Department of Endocrinology, Heilongjiang Academy of Traditional Chinese Medicine, Harbin, China; 11 Department of Endocrinology, Affiliated Hospital of Shaanxi University of Traditional Chinese Medicine, Xianyang, China; 12 Department of Endocrinology, Shenzhen Hospital (Futian) of Guangzhou University of Chinese Medicine, Shenzhen, China; 13 Department of Endocrinology, Tongliao City Horqin District First People’s Hospital, Tongliao, China; 14 Department of Endocrinology, Baoji Second People’s Hospital, Baoji, China; 15 Department of Endocrinology, Hebei Cangzhou Hospital of Integrated Chinese and Western Medicine, Cangzhou, China; 16 Department of Traditional Chinese Medicine, Nanjing Drum Tower Hospital, Nanjing, China; 17 Geriatrics Department, Liaocheng People’s Hospital, Liaocheng, China; 18 Department of Endocrinology, Second Affiliated Hospital of Yunnan University of Chinese Medicine, Kunming, China; 19 Department of Endocrinology, The First Affiliated Hospital of Guangdong Pharmaceutical University, Guangzhou, China; 20 Department of Biostatistics, Peking University First Hospital, Beijing, China; 21 Department of Endocrinology, Hebei General Hospital, Shijiazhuang, China; 22 NHC Key Laboratory of Hormones and Development, Chu Hsien-I Memorial Hospital and Tianjin Institute of Endocrinology, Tianjin Medical University, Tianjin, China; 23 Center for Drug Clinical Research, Shanghai University of Traditional Chinese Medicine, Shanghai, China; 24 Department of Endocrinology, Peking Union Medical College Hospital, Beijing, China; 25 Department of Endocrinology, Sun Yat-Sen Memorial Hospital, Sun Yai-Sen University, Guangzhou, China; 26 Metabolic Disease Institute, Guang’anmen Hospital China Academy of Chinese Medical Sciences, Beijing, China; 27 State Key Laboratory for Innovation and Transformation of *Luobing* Theory of Hebei Yiling Hospital, Shijiazhuang, Hebei Province, China

## Abstract

**Importance:**

Previous studies have shown that Jinlida (JLD) granules, an approved treatment for type 2 diabetes in China, can reduce blood glucose level, reduce glycated hemoglobin (HbA_1c_), and improve insulin resistance in people with type 2 diabetes.

**Objective:**

To evaluate the effect of long-term administration of JLD vs placebo on the incidence of diabetes in participants with impaired glucose tolerance (IGT) and multiple metabolic abnormalities.

**Design, Setting, and Participants:**

This multicenter, double-blind, placebo-controlled randomized clinical trial (FOCUS) was conducted across 35 centers in 21 cities in China from June 2019 to February 2023. Individuals aged 18 to 70 years with IGT and multiple metabolic abnormalities were enrolled.

**Intervention:**

Participants were randomly allocated 1:1 to receive JLD or placebo (9 g, 3 times per day, orally). They continued this regimen until they developed diabetes, withdrew from the study, were lost to follow-up, or died.

**Main Outcomes and Measures:**

The primary outcome was the occurrence of diabetes, which was determined by 2 consecutive oral glucose tolerance tests. Secondary outcomes included waist circumference; fasting and 2-hour postprandial plasma glucose levels; HbA_1c_; fasting insulin level; homeostatic model assessment for insulin resistance (HOMA-IR); total cholesterol, low-density lipoprotein cholesterol, and triglyceride levels; ankle-brachial index; and carotid intima-media thickness.

**Results:**

A total of 889 participants were randomized, of whom 885 were in the full analysis set (442 in the JLD group; 443 in the placebo group; mean [SD] age, 52.57 [10.33] years; 463 [52.32%] female). Following a median observation period of 2.20 years (IQR, 1.27-2.64 years), participants in the JLD group had a lower risk of developing diabetes compared with those in the placebo group (hazard ratio, 0.59; 95% CI, 0.46-0.74; *P* < .001). During the follow-up period, the JLD group had a between-group difference of 0.95 cm (95% CI, 0.36-1.55 cm) in waist circumference, 9.2 mg/dL (95% CI, 5.4-13.0 mg/dL) in 2-hour postprandial blood glucose level, 3.8 mg/dL (95% CI, 2.2-5.6 mg/dL) in fasting blood glucose level, 0.20% (95% CI, 0.13%-0.27%) in HbA_1c_, 6.6 mg/dL (95% CI, 1.9-11.2 mg/dL) in total cholesterol level, 4.3 mg/dL (95% CI, 0.8-7.7 mg/dL) in low-density lipoprotein cholesterol level, 25.7 mg/dL (95% CI, 15.9-35.4 mg/dL) in triglyceride levels, and 0.47 (95% CI, 0.12-0.83) in HOMA-IR compared with the placebo group. After 24 months of follow-up, the JLD group had a significant improvement in ankle-brachial index and waist circumference compared with the placebo group.

**Conclusions and Relevance:**

The findings suggest that JLD can reduce the risk of diabetes in participants with IGT and multiple metabolic abnormalities.

**Trial Registration:**

Chinese Clinical Trial Register: ChiCTR1900023241

## Key Points

**Question:**

Can long-term use of Jinlida granules (JLD) reduce the incidence of diabetes in participants with impaired glucose tolerance (IGT) and multiple metabolic abnormalities?

**Findings:**

In this randomized clinical trial of 889 participants with IGT and multiple metabolic abnormalities, after a median follow-up of 2.20 years, the JLD group had a significantly lower risk of developing diabetes compared with the placebo group.

**Meaning:**

In participants with IGT combined with multiple metabolic disorders, JLD reduced the incidence of diabetes compared with placebo.

## Teaser

This randomized clinical trial investigates whether long-term use of Jinlida granules decreases the incidence of diabetes in participants with impaired glucose tolerance and multiple metabolic abnormalities.

## Introduction

Diabetes ranks among the world’s most pressing health concerns. Prediabetes, encompassing impaired fasting glucose (IFG), impaired glucose tolerance (IGT), and their combined state, poses a significant challenge. The American Diabetes Association reports that up to 50% of individuals with prediabetes progress to diabetes within 5 years.^1^ Notably, IGT alone is associated with a significantly increased risk of developing diabetes and cardiovascular disease (CVD) compared with IFG alone.^2^ When IGT co-occurs with additional risk factors like obesity, dyslipidemia, and hypertension, the risk of diabetes increases further and the CVD risk has been shown to increase by 34%.^3,4^ Therefore, robust management strategies for individuals with IGT and multiple metabolic abnormalities are urgently needed. Existing studies have shown that intensive lifestyle modifications and pharmacologic interventions can slow the progression from IGT to diabetes.^5,6,7,8,9,10^ To date and to our knowledge, no randomized clinical trials (RCTs) have conclusively proven the effectiveness of traditional Chinese medicine (TCM) in lowering the risk of diabetes among individuals with IGT and multiple metabolic abnormalities.

Jinlida (JLD) granules are a TCM compound granule preparation comprising 17 herbal ingredients (eAppendix in Supplement 1). Approved by the Chinese National Medical Products Administration in 2005 for treating type 2 diabetes, JLD has demonstrated potential in alleviating insulin resistance, lowering blood glucose level and glycated hemoglobin (HbA_1c_), and even reversing IGT.^11,12,13,14^ Further foundational research underscores the comprehensive benefits of JLD, including reports of decreased weight and waist circumference in mice,^15^ along with significant improvements in high-fat diet–induced obesity and fat accumulation.^15^ Importantly, JLD promotes glucose and lipid homeostasis, ameliorates hepatic steatosis and inflammation, and significantly activates brown adipose tissue thermogenesis in high-fat diet–induced obese mice, enhancing mitochondrial biogenesis and fatty acid oxidation metabolism.^16^ Additionally, JLD significantly reduces insulin resistance in high-fat diet–fed rats, thereby improving hyperglycemia, hyperinsulinemia, and hyperlipidemia. Its capacity to modulate glucose and lipid metabolism while reducing hepatic oxidative stress adds to its therapeutic potential.^17^ Grounded on initial clinical findings and experimental evidence, our hypothesis was that JLD could diminish the diabetes risk among individuals with IGT and metabolic abnormalities by enhancing insulin sensitivity.

## Methods

### Study Design

The trial protocol and statistical analysis plan for this multicenter, double-blind, placebo-controlled RCT (FOCUS; ChiCTR1900023241) are given in Supplement 2. Detailed methods are available in the eMethods in Supplement 1 and in published literature.^18^ Conducted in accordance with the Declaration of Helsinki,^19^ Good Clinical Practice guidelines, and Chinese regulations, the study received independent ethics committee approval at all participating centers. All participants provided written informed consent. This study followed the Consolidated Standards of Reporting Trials (CONSORT) Extension for Chinese Herbal Medicine Formulas 2017 reporting guideline.^20^

### Participants

The inclusion criteria were determined according to metabolic syndrome diagnostic criteria in the Chinese Type 2 Diabetes Prevention and Treatment Guidelines (2017 edition).^21^ The study was conducted from June 2019 to February 2023 at 35 centers in 21 cities across China.

Inclusion criteria were age 18 to 70 years, abdominal obesity (waist circumference ≥90 cm for men or ≥85 cm for women), presence of IGT diagnostic criteria (ie, fasting blood glucose level <126 mg/dL and 2-hour postprandial blood glucose level ≥140 mg/dL and <200 mg/dL [to convert to mmol/L, multiply by 0.0555]), and at least 1 of the following conditions: hypertension (blood pressure ≥130/85 mm Hg and/or confirmed hypertension and treatment), fasting triglyceride levels of 150.44 mg/dL or greater (to convert to mmol/L, multiply by 0.0113), or fasting high-density lipoprotein cholesterol (HDL-C) level less than 40.15 mg/dL (to convert to mmol/L, multiply by 0.0259). Exclusion criteria were history of type 1 or type 2 diabetes, use of hypoglycemic drugs within the past 3 months, hyperthyroidism or hypothyroidism, uncontrolled hypertension or hypotension, severe liver or kidney dysfunction (glutamic pyruvic transaminase or alanine aminotransferase level >3 times the upper limit of normal or creatinine level >1.49 mg/dL [to convert to μmol/L, multiply by 88.4]), other serious organ diseases (eg, severe organic heart disease), and pregnancy or breastfeeding.

### Randomization and Blinding

To ensure rigorous randomization and blinding, a block randomized design was used (eMethods in Supplement 1). An interactive web response system was used to centrally manage participant allocation. Randomization numbers were generated independently by a statistician from the Peking University Clinical Institute using SAS, version 9.4 (SAS Institute Inc). A comprehensive blinding protocol was implemented to safeguard treatment allocation concealment prior to randomization among participants, investigators, and other trial personnel. To maintain blinding integrity, placebo granules were meticulously matched with JLD in terms of color, odor, taste, shape, texture, specifications, appearance, packaging, labeling, and identification, making it virtually impossible for participants to distinguish them (eAppendix in Supplement 1).

### Intervention

All investigators were proficient in diabetes management and underwent comprehensive protocol training prior to the study commencement. Throughout the induction and follow-up phases, all participants received an ongoing lifestyle intervention, including monthly sessions offering at least 20 minutes of professional guidance according to Chinese Type 2 Diabetes Prevention and Treatment Guidelines (2017 edition).^21^ Additionally, they received a standardized lifestyle intervention booklet offering specific recommendations for adjusting daily habits, including engaging in regular physical activity; regulating intake of macronutrients (protein, carbohydrates), dietary fiber, and trace elements; and reducing sodium consumption.

**Figure 1.**
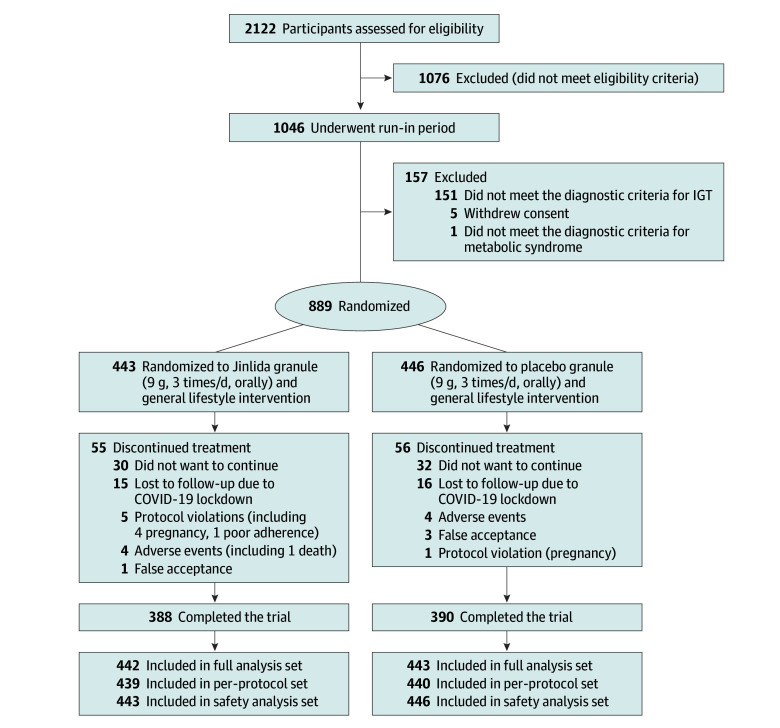
Enrollment, Randomization, and Follow-Up of Study Participants IGT indicates impaired glucose tolerance.

Following a 1-month lifestyle intervention induction period, participants were randomly assigned 1:1 to either the JLD group (9 g, 3 times per day, orally) or the placebo group (9 g, 3 times per day, orally). Both groups continued receiving lifestyle intervention throughout the study. The JLD granules and placebo granules were provided by Shijiazhuang Yiling Pharmaceutical Co, Ltd. The use of any other oral or injectable antidiabetic medications or health products, including TCM with hypoglycemic effects, was strictly prohibited during the study. Investigators conducted monthly visits during which participants underwent examinations and tests per the study protocol. Additionally, adherence to the lifestyle intervention and study drug use was evaluated, and participants received individualized guidance on further lifestyle adjustments. This continued until the study was completed or until participants developed diabetes, withdrew, were lost follow-up, or died. The study flowchart is presented in Figure 1.

### Outcomes

The primary outcome was the development of diabetes, defined according to Chinese Type 2 Diabetes Prevention and Treatment Guidelines (2017 edition).^21^ Participants underwent an oral glucose tolerance test (OGTT) every 3 months. If their fasting plasma glucose level was 126 mg/dL or greater or their 2-hour postprandial glucose level was 200 mg/dL or greater, the OGTT was repeated 1 week later for confirmation. Throughout the study, participants underwent capillary blood glucose monitoring for fasting glucose and 2-hour postprandial glucose levels using a glucometer every month. If these levels met the criteria, confirmatory OGTT was performed. After a center’s principal investigator determined that a participant had reached the end point, the participant’s data were submitted to the end point adjudication committee for final confirmation.

Secondary outcome measures included waist circumference; blood pressure; fasting and 2-hour postprandial plasma glucose levels; HbA_1c_; fasting insulin level; homeostatic model assessment for insulin resistance (HOMA-IR); total cholesterol, low-density lipoprotein cholesterol (LDL-C), HDL-C, and triglyceride levels; ankle-brachial index (ABI); and carotid intima-media thickness (CIMT).

### Quality Control

The study was governed by an academic committee, a data safety and monitoring board (DSMB), and an end point adjudication committee (eMethods in Supplement 1). Participant safety and end point adjudication were conducted independently by the DSMB and end point adjudication committee, respectively. All center laboratories held interlaboratory quality assessment certificates (National Center for Clinical Laboratories external quality assessment certificates) ensuring precise and reliable measurements of blood pressure, CIMT, and ABI adhered to by following standardized operating procedures. Notably, this project spanned the COVID-19 pandemic. During brief lockdowns in various regions of China, researchers adapted to maintain participant adherence by implementing a combination of remote and home visits.

### Statistical Analysis

The primary end point, incidence of diabetes, was compared between groups using a log-rank test. For further analysis, a Cox proportional hazards regression model incorporating age, sex, and study center as covariates was used to estimate the hazard ratio (HR) and 95% CI between treatment groups. Additionally, Kaplan-Meier curves were generated to visualize survival differences. Assuming a 4-year study duration (2 years of enrollment, 2 years of follow-up), 40% diabetes incidence in the placebo group, and 880 participants with 380 diabetes events, the study achieved at least 80% power to detect a risk ratio of 0.75 between groups at a 2-sided α level of .05.

For secondary repeated measures (eg, changes from baseline in waist circumference, 2-hour postprandial glucose level), repeated-measures mixed models with unstructured variance-covariance structures were used. The model included group, visit, group × visit interaction, and baseline value as terms. The ABI and CIMT comparisons between groups used the Wilcoxon rank sum test.

The full analysis set (FAS) for primary and secondary end point analyses comprised all randomized participants meeting inclusion criteria and receiving at least 1 study drug dose. The safety analysis included participants who received at least 1 dose. Statistical analyses adhered to the prespecified plan (statistical analysis plan in Supplement 2) and were conducted in SAS, version 9.4; 2-sided *P* < .05 denoted statistical significance.

## Results

### Study Population

The trial commenced with its first participant in June 2019, and by April 2021, a total of 889 participants had been randomized. Follow-up extended until February 2023, with the median follow-up duration being 2.20 years (IQR, 1.27-2.64 years; mean [SD], 2.01 [0.90] years) and the longest at 3.43 years. The last participant enrolled was diagnosed with diabetes and exited the study in February 2023. Among initial participants, 111 withdrew (55 in the JLD group and 56 in the placebo group). Notably, 31 withdrawals (15 from the JLD group and 16 from the placebo group) were attributed to the COVID-19 pandemic. One participant died during the trial. Ultimately, 885 participants (442 in the JLD group and 443 in the placebo group) comprised the FAS for subsequent analysis (Figure 1). Of these participants, 463 (52.32%) were female and 422 (47.68%) were male; mean (SD) age was 52.57 (10.33) years.

### Baseline Demographic and Clinical Characteristics of Participants

The Table presents the baseline characteristics of participants in the FAS. The JLD and placebo groups had similar distributions for age, sex, waist circumference, fasting and 2-hour postprandial glucose levels, HbA_1c_, fasting insulin level, blood pressure, lipid levels, HOMA-IR, ABI, CIMT, and the prevalence of comorbidities, use of antihypertensive agents, and use of lipid-lowering agents.

**Table.** Baseline Characteristics of the Participants in the Full Analysis Set^a^

Characteristic	JLD group (n = 442)	Placebo group (n = 443)	Total (N = 885)
Age, mean (SD), y	52.26 (10.10)	52.88 (10.57)	52.57 (10.33)
Age group, y			
18-44	100 (22.62)	101 (22.80)	201 (22.71)
45-59	222 (50.23)	204 (46.05)	426 (48.14)
≥60	120 (27.15)	138 (31.15)	258 (29.15)
Sex			
Female	224 (50.68)	239 (53.95)	463 (52.32)
Male	218 (49.32)	204 (46.05)	422 (47.68)
Waist circumference, mean (SD), cm	94.88 (8.24)	94.86 (8.52)	94.87 (8.38)
BMI, mean (SD)	26.71 (3.01)	26.93 (3.39)	26.82 (3.21)
Fasting plasma glucose level, mean (SD), mg/dL	105.8 (12.4)	105.2 (12.3)	105.6 (12.4)
2-h Postprandial glucose level, mean (SD), mg/dL	164.7 (18.0)	164.9 (18.0)	164.7 (18.0)
Glycated hemoglobin, mean (SD), %	5.87 (0.61)	5.84 (0.64)	5.85 (0.62)
Fasting insulin level, mean (SD), mIU/L	13.11 (9.13)	14.10 (10.81)	13.61 (10.02)
HOMA-IR, mean (SD)	3.40 (2.29)	3.67 (2.93)	3.54 (2.64)
Systolic blood pressure, mean (SD), mm Hg	135.03 (11.62)	134.03 (10.56)	134.53 (11.10)
Diastolic blood pressure, mean (SD), mm Hg	84.88 (8.40)	83.84 (7.42)	84.36 (7.94)
Total cholesterol level, mean (SD), mg/dL	196.1 (39.4)	197.3 (44.0)	196.5 (41.7)
LDL cholesterol level, mean (SD), mg/dL	116.2 (32.8)	117.0 (35.1)	116.6 (34.0)
HDL cholesterol level, mean (SD), mg/dL	49.0 (12.4)	49.4 (12.0)	49.0 (12.0)
Triglyceride level, mean (SD), mg/dL	180.5 (108.0)	177.0 (100.9)	178.8 (104.4)
Carotid intima-media thickness, mean (SD), mm	1.07 (0.56)	1.12 (0.63)	1.10 (0.60)
Ankle-brachial index, mean (SD)	1.11 (0.12)	1.11 (0.11)	1.11 (0.12)
Medical history			
Dyslipidemia	277 (62.67)	283 (63.88)	560 (63.28)
Hypertension	183 (41.40)	187 (42.21)	370 (41.81)
Coronary heart disease	34 (7.69)	39 (8.80)	73 (8.25)
Antihypertensive medication			
Calcium channel blocker	66 (14.93)	71 (16.03)	137 (15.48)
Renin-angiotensin system inhibitors	28 (6.33)	42 (9.48)	70 (7.91)
β-Blocker	15 (3.39)	19 (4.29)	34 (3.84)
Lipid regulation agents			
Statin	70 (15.84)	73 (16.48)	143 (16.16)
Fibrates	0	3 (0.68)	3 (0.34)

Abbreviations: BMI, body mass index (calculated as weight in kilograms divided by height in meters squared); HDL, high-density lipoprotein; HOMA-IR, homeostatic model assessment for insulin resistance; JLD, Jinlida; LDL, low-density lipoprotein.

SI conversion factors: To convert glucose to millimoles per liter, multiply by 0.0555; glycated hemoglobin to proportion of total hemoglobin, multiply by 0.01; HDL, LDL, and total cholesterol to millimoles per liter, multiply by 0.0259; and triglycerides to millimoles per liter, multiply by 0.0113.

^a^ Data are presented as number (percentage) of participants unless otherwise indicated.

### Cumulative Incidence and HR of Diabetes

**Figure 2.**
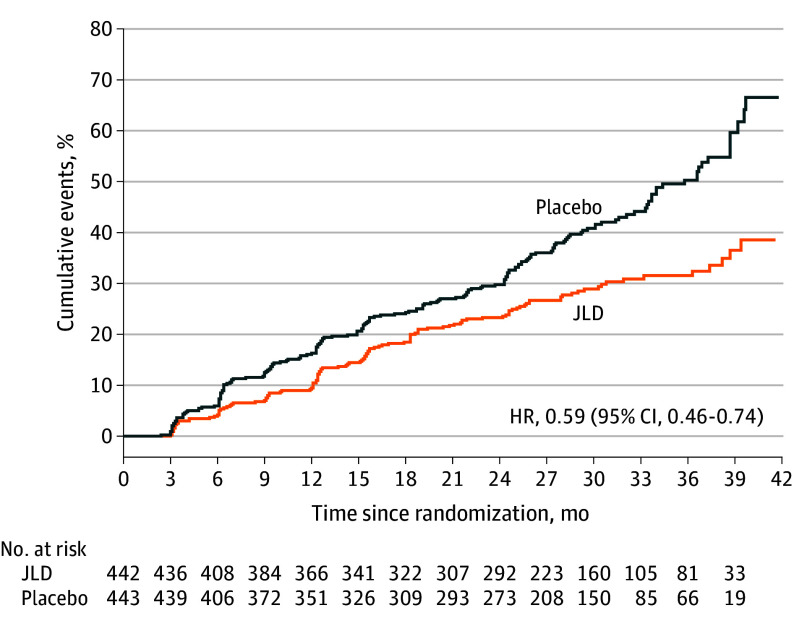
Kaplan-Meier Curve for Time to Diabetes in the Jinlida (JLD) and Placebo Groups HR indicates hazard ratio.

During the median follow-up of 2.20 years, 27.83% (123 of 442) in the JLD group and 42.66% (189 of 443) in the placebo group developed diabetes, representing a relative risk reduction in the JLD group (HR, 0.59; 95% CI, 0.46-0.74; *P* < .001) (Figure 2). The sensitivity analysis results suggest that the results of the FAS analysis were robust (eTable 1 in Supplement 1). Annual incidence rates per person-year were 13.70% and 22.05% in the JLD and placebo groups, respectively (relative risk, 0.62; 95% CI, 0.49-0.78; *P* < .001). Diabetic incidence diverged over time, with 12-month rates at 9.95% and 15.80% (*P* = .007), 24-month rates at 21.72% and 29.12% (*P* = .006), and 36-month rates at 26.92% and 40.18% (*P* < .001) for the JLD and placebo groups, respectively. Notably, among participants completing the study, 39.18% (152 of 388) in the JLD group and 25.64% (100 of 390) in the placebo group achieved normal glucose tolerance (*P* < .001).

### Stratified Analysis

**Figure 3.**
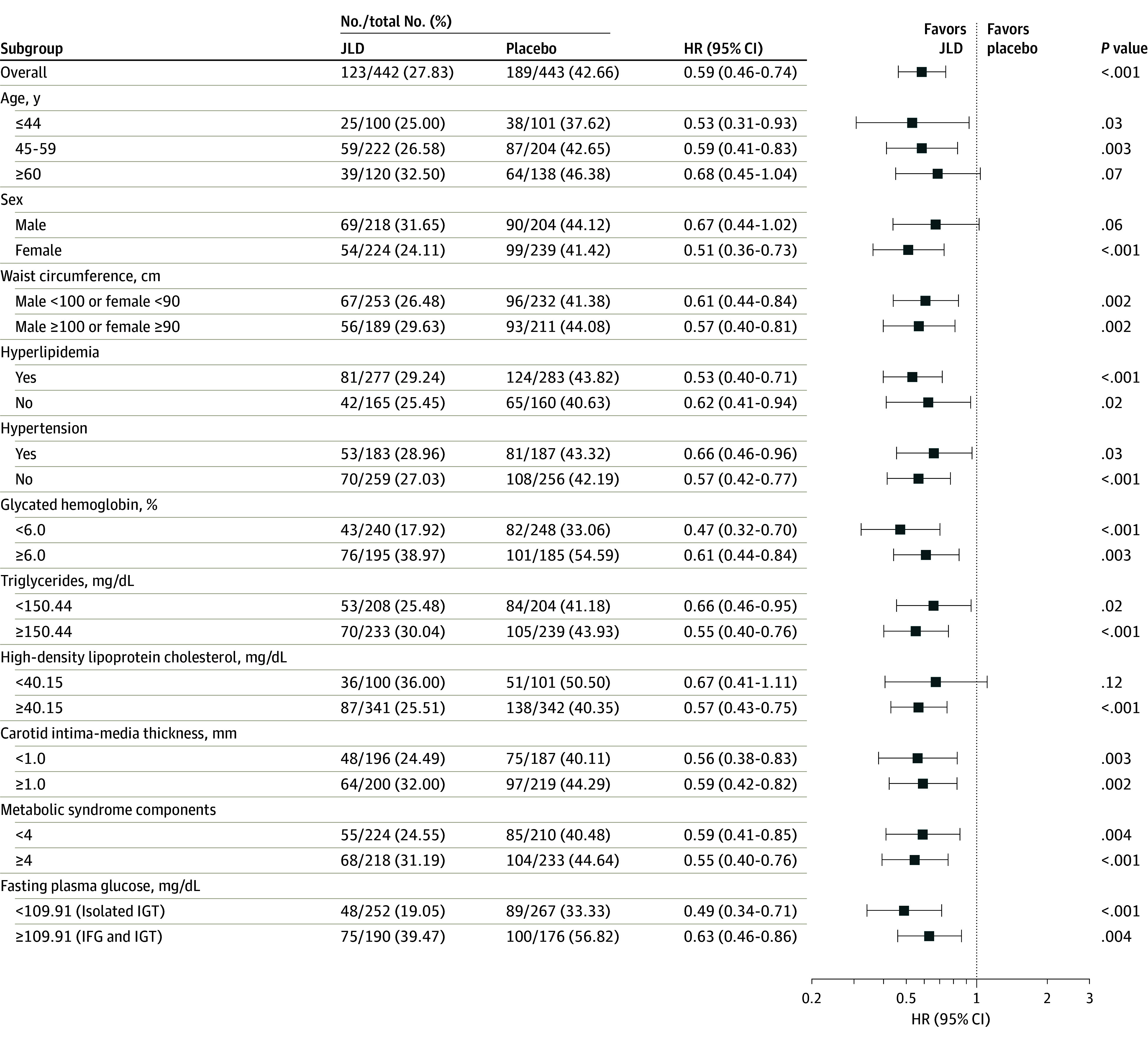
Incidence Rates and Hazard Ratios (HRs) for the Full Analysis Set Population and Selected Subgroups To convert glucose to millimoles per liter, multiply by 0.0555; glycated hemoglobin to proportion of total hemoglobin, multiply by 0.01; high-density lipoprotein cholesterol to millimoles per liter, multiply by 0.0259; and triglycerides to millimoles per liter, multiply by 0.0113. IFG indicates impaired fasting glucose; IGT, impaired glucose tolerance; and JLD, Jinlida.

Subgroup analyses of diabetes incidence (Figure 3) mirrored the overall trend, with JLD consistently reducing rates across diverse participant profiles. This effect held true regardless of age, sex, waist circumference, preexisting hyperlipidemia or hypertension, glycemic markers, lipid profiles, CIMT, or metabolic syndrome components. Notably, the benefit of JLD extended to individuals with IGT both with and without IFG.

As shown in Figure 3, subgroup analyses of diabetes incidence revealed that the effect of JLD on diabetes incidence was consistent with the overall trend, with lower incidence in the JLD group than in the placebo group across all subgroups defined by age, sex, waist circumference, history of hyperlipidemia and hypertension, HbA_1c_, triglyceride and HDL-C levels, CIMT, and metabolic syndrome components. This effect was also observed in individuals with IGT both with and without IFG.

### Secondary Outcomes

**Figure 4.**
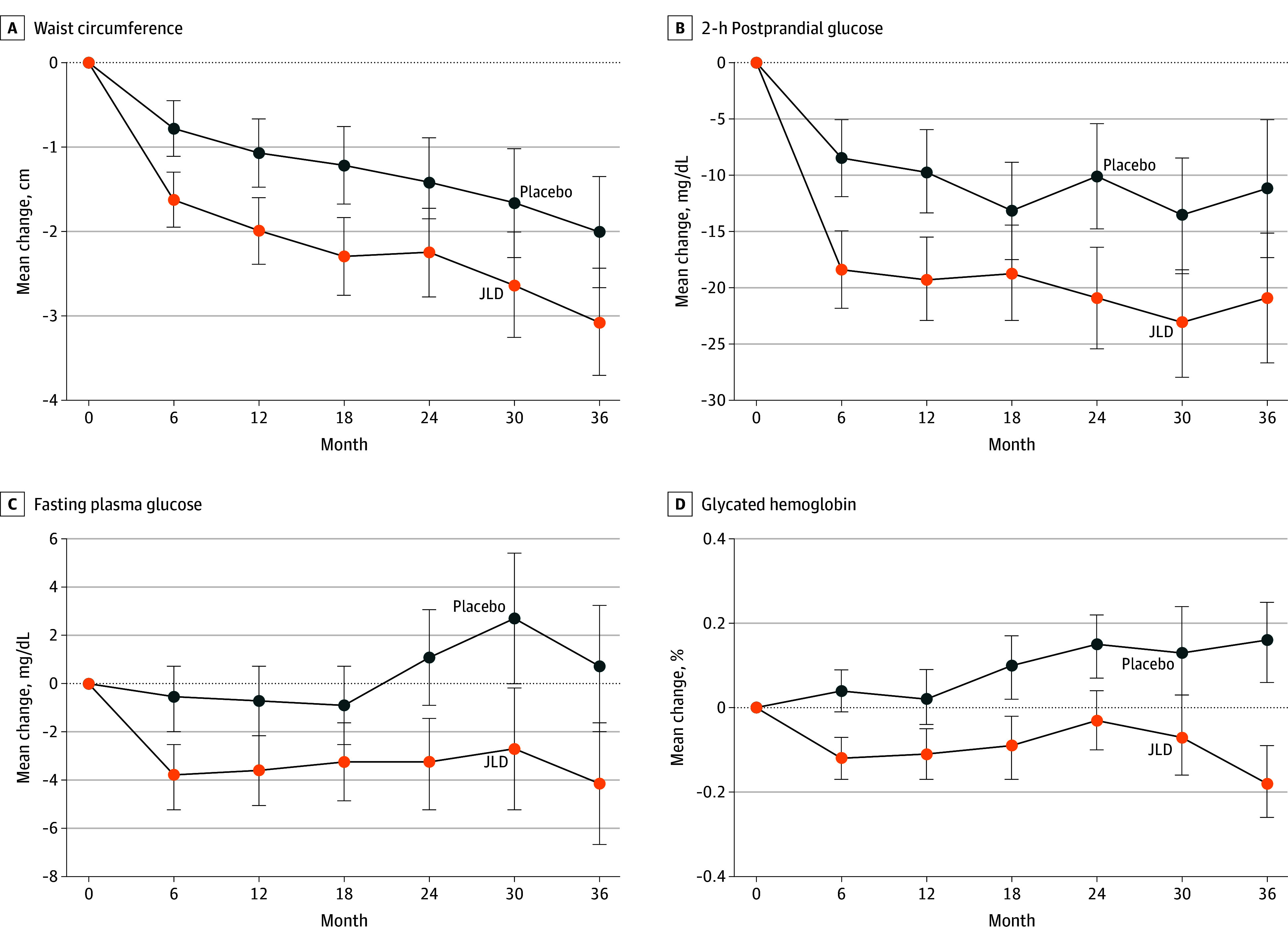
Effects of Jinlida (JLD) on Waist Circumference and Blood Glucose Indexes During the study, the mean change and 95% CI for continuous measurements were calculated using mixed-effects regression models. The models were fitted to all available data for each measurement. *P* values assessed the significance of between-group differences in mean changes (*P* < .001 for all differences except waist circumference [*P* = .002]). Error bars represent 95% CIs. To convert glucose to millimoles per liter, multiply by 0.0555, and glycated hemoglobin to proportion of total hemoglobin, multiply by 0.01.

Both waist circumference and body mass index (calculated as weight in kilograms divided by height in meters squared) significantly differed between the groups (Figure 4A and eFigure 1 in Supplement 1). Compared with the placebo group, the JLD group demonstrated greater reductions in waist circumference (2.31 vs 1.36 cm) and body mass index (0.41 vs 0.14), yielding significant between-group differences of 0.95 cm (95% CI, 0.36-1.55 cm; *P* = .002) (Figure 4A) and 0.27 (95% CI, 0.09-0.44; *P* = .003) (eFigure 1 in Supplement 1), respectively.

The JLD group showed superior improvement in blood glucose level and HbA_1c_ compared with the placebo group (Figure 4B-D). Postprandial 2-hour glucose level decreased by 20.2 mg/dL in the JLD group and 11.0 mg/dL in the placebo group, yielding a significant between-group difference of 9.2 mg/dL (95% CI, 5.4-13.0 mg/dL; *P* < .001) (Figure 4B). Similarly, fasting glucose level decreased by 3.4 mg/dL in the JLD group compared with a 0.4 mg/dL increase in the placebo group, resulting in a significant difference of 3.8 mg/dL (95% CI, 2.2-5.6 mg/dL; *P* < .001) (Figure 4C). Notably, HbA_1c_ decreased by 0.10% (to convert to proportion of total hemoglobin, multiply by 0.01) in the JLD group and increased by 0.10% in the placebo group, showing a significant between-group difference of 0.20% (95% CI, 0.13%-0.27%; *P* < .001) (Figure 4D).

Jinlida granules showed a beneficial effect on lipid profiles. Total cholesterol level was reduced by 12.4 and 5.8 mg/dL in the JLD and placebo groups, respectively, with a between-group difference of 6.6 mg/dL (95% CI, 1.9-11.2 mg/dL; *P* = .007) (to convert to mmol/L, multiply by 0.0259) (eFigure 2 in Supplement 1). The LDL-C level was reduced by 8.9 and 4.6 mg/dL in the JLD and placebo groups, respectively, with a between-group difference of 4.3 mg/dL (95% CI, 0.8-7.7 mg/dL; *P* = .02) (to convert to mmol/L, multiply by 0.0259). The HDL-C level was increased by 2.3 and 0.8 mg/dL in the JLD and placebo groups, respectively, with a between-group difference of 1.5 mg/dL (95% CI, −3.9 to 3.1 mg/dL; *P* = .10). Triglyceride levels were reduced by 23.0 mg/dL in the JLD group and increased by 2.7 mg/dL in the placebo group, with a between-group difference of 25.7 mg/dL (95% CI, 15.9-35.4 mg/dL; *P* < .001).

Systolic and diastolic blood pressure reductions were evident in both groups, but the changes did not differ significantly between them (eFigure 3 in Supplement 1). HOMA-IR decreased by 0.23 in the JLD group and increased by 0.25 in the placebo group, with a between-group difference of 0.47 (95% CI, 0.12-0.83; *P* = .009) (eFigure 4 in Supplement 1).

In the JLD group, CIMT decreased by a mean (SD) of 0.05 (0.53) mm after 24-month treatment, while it increased by a mean (SD) of 0.03 (0.41) mm in the placebo group. The between-group difference was statistically significant. In the JLD group, the ABI decreased by a mean (SD) of 0.00 (0.10), while it increased by a mean (SD) of 0.02 (0.10) in the placebo group. The between-group difference was also statistically significant.

### Adverse Event Analyses

Of the 889 participants in the safety analysis (443 in the JLD group, 446 in the placebo group), 420 (94.81%) in the JLD group and 410 (91.93%) in the placebo group reported adverse events, with 4 (0.90%) per group withdrawing due to these events (eTable 2 in Supplement 1). Notably, 1 participant in the JLD group died by suicide related to depression; DSMB experts deemed this unrelated to the drug.

## Discussion

The findings from both the Da Qing IGT and Diabetes Study^22^ in China and the Diabetes Prevention Program^23^ in the US have substantiated the efficacy of intensive lifestyle interventions in preventing diabetes. However, the practical application of such intensive lifestyle interventions faces challenges, primarily due to difficulties in maintaining long-term adherence to behavioral changes among participants.^22,23^ The exploration of strategies that combine lifestyle interventions with pharmacologic treatments to delay the onset of diabetes remains an area of interest.

The STOP-NIDDM trial^10^ demonstrated that combining lifestyle changes with acarbose reduced the incidence of diabetes by 32% compared with 42% in the group receiving lifestyle interventions plus a placebo. Similarly, the addition of metformin to lifestyle modifications resulted in an incidence of diabetes of 29.8% compared with 36.2% in the group receiving lifestyle modifications combined with a placebo.^6^ To our knowledge, our study was the first to investigate the synergistic effects of TCM and lifestyle modifications among participants with IGT, abdominal obesity, and metabolic disorders. Our findings showed that incorporating JLD treatment with lifestyle modifications substantially decreased the risk of developing diabetes by 41% over a median 2.2-year follow-up period. Moreover, JLD exhibited positive effects on secondary health markers, such as waist circumference; postprandial and fasting blood glucose levels; HbA_1c_; total cholesterol, LDL-C, and triglyceride levels; and the HOMA-IR. These results suggest a significant role of JLD in enhancing insulin sensitivity and managing lipid metabolism disorders while demonstrating favorable drug safety profiles throughout the study duration. Although extensive research, including the Diabetes Prevention Program,^23^ Finnish Diabetes Prevention Study,^24^ and Da Qing study,^22^ has affirmed the lasting benefits of rigorous lifestyle interventions in preventing type 2 diabetes, the evidence for such interventions in reducing the risk of CVD or microvascular complications remains scant.^25^ Our study further revealed that JLD notably improved the ABI^26,27^ and CIMT,^28,29,30,31^ crucial indicators of arterial stiffness and predictors of CV and cerebrovascular disease risk, suggesting the necessity for prolonged studies to validate the long-term CV protective effects of combining JLD with lifestyle interventions.

Comprehensive basic research has illuminated the multifaceted regulatory effects of JLD on diabetes progression. These effects include the stabilization of glucose and lipid levels, reduction in insulin resistance,^17^ and diminishment of obesity and ectopic fat accumulation prompted by a high-fat diet. Investigations into the individual constituents of JLD further revealed their metabolic remedial properties (eFigure 5 in Supplement 1), suggesting the synergistic efficacy of these herbal components. Preliminary findings underscore the potential for JLD as a therapeutic agent in diabetes management.

### Limitations

This study has limitations. First, the study’s participant base, consisting solely of a Chinese population, raises questions about the applicability of the findings across different ethnic groups. This necessitates additional studies to explore the generalizability of the results. Second, the study duration was insufficient to capture the occurrence of CV events requiring longer-term follow-up. Third, identifying the specific active ingredients in JLD responsible for its primary effects remains a critical area for subsequent investigation.

## Conclusions

This RCT demonstrated that JLD can lower the risk of IGT progressing to diabetes by ameliorating multiple metabolic abnormalities. Jinlida granules were found to be safe and effective, offering a promising intervention for participants with IGT with multiple metabolic disorders to prevent diabetes onset.

## Notes

Supplement 1. **eFigure 1.** Mean Changes of BMI in the JLD and Placebo **eFigure 2.** Mean Changes of Blood Lipids in the JLD and Placebo **eFigure 3.** Mean Changes of Blood Pressure in the JLD and Placebo **eFigure 4.** Effects of JLD on HOMA-IR **eFigure 5.** The Herb-Compound-Target Network of JLD Regulating Insulin Resistance Pathway **eTable 1.** Sensitivity Analysis of Diabetes Incidence **eTable 2.** Adverse Events **eMethods.** Methodologic Appendix **eAppendix.** Detailed Information of Jinlida Granules and Placebos

Supplement 2.
Trial Protocol and Statistical Analysis Plan

Supplement 3.
FOCUS Trial Committees and Investigators

Supplement 4.
Data Sharing Statement
