# Supplementary material for: Jinlida for Diabetes Prevention in Impaired Glucose Tolerance and Multiple Metabolic Abnormalities: The FOCUS Randomized Clinical Trial
Source: JAMA Intern Med. 2024 Jun 3;184(7):727–35. doi: 10.1001/jamainternmed.2024.1190 (PMC11148787; doi:10.1001/jamainternmed.2024.1190)
Supplement: Supplement 1. — eFigure 1. Mean Changes of BMI in the JLD and Placebo eFigure 2. Mean Changes of Blood Lipids in the JLD and Placebo eFigure 3. Mean Changes of Blood Pressure in the JLD and Placebo eFigure 4. Effects of JLD on HOMA-IR eFigure 5. The Herb-Compound-Target Network of JLD Regulating Insulin Resistance Pathway eTable 1. Sensitivity Analysis of Diabetes Incidence eTable 2. Adverse Events eMethods. Methodologic Appendix eAppendix. Detailed Information of Jinlida Granules and Placebos [file jamainternmed-e241190-s001.pdf]

Supplementary Online Content

Ji H, Zhao X, Chen X, et al. Jinlida for diabetes prevention in impaired glucose tolerance and multiple metabolic abnormalities: the FOCUS randomized clinical trial. *JAMA Intern Med*. Published online June 3, 2024.
doi:10.1001/jamainternmed.2024.1190

eFigure 1. Mean Changes of BMI in the JLD and Placebo

eFigure 2. Mean Changes of Blood Lipids in the JLD and Placebo

eFigure 3. Mean Changes of Blood Pressure in the JLD and Placebo

eFigure 4. Effects of JLD on HOMA-IR

eFigure 5. The Herb-Compound-Target Network of JLD Regulating Insulin Resistance Pathway

eTable 1. Sensitivity Analysis of Diabetes Incidence

eTable 2. Adverse Events

eMethods. Methodologic Appendix

eAppendix. Detailed Information of Jinlida Granules and Placebos

This supplementary material has been provided by the authors to give readers additional information about their work.

© 2024 Ji H et al. *JAMA Internal Medicine.*

eFigure 1. Mean changes of BMI in the JLD and placebo

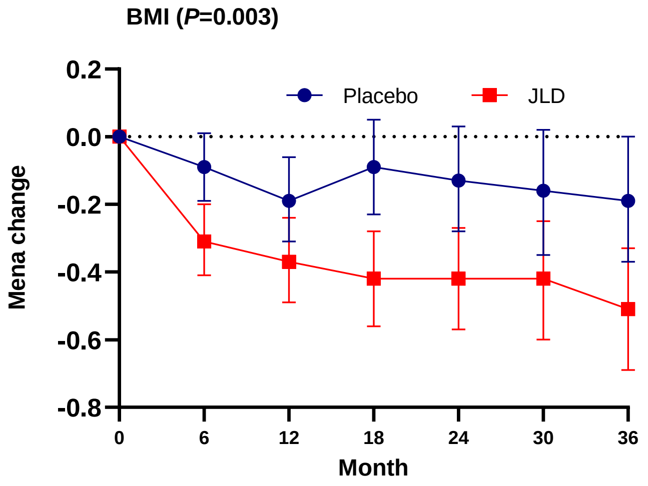

Note: During the study, the mean change and 95% confidence interval (CI) for continuous measurements were calculated using mixed-effects regression models (MMRM). The models were fit to all available data for each measurement. P-values assessed the significance of between-group differences in mean changes, with error bars representing the 95% CI bounds.

© 2024 Ji H et al. *JAMA Internal Medicine.*

eFigure 2. Mean changes of blood lipids in the JLD and placebo

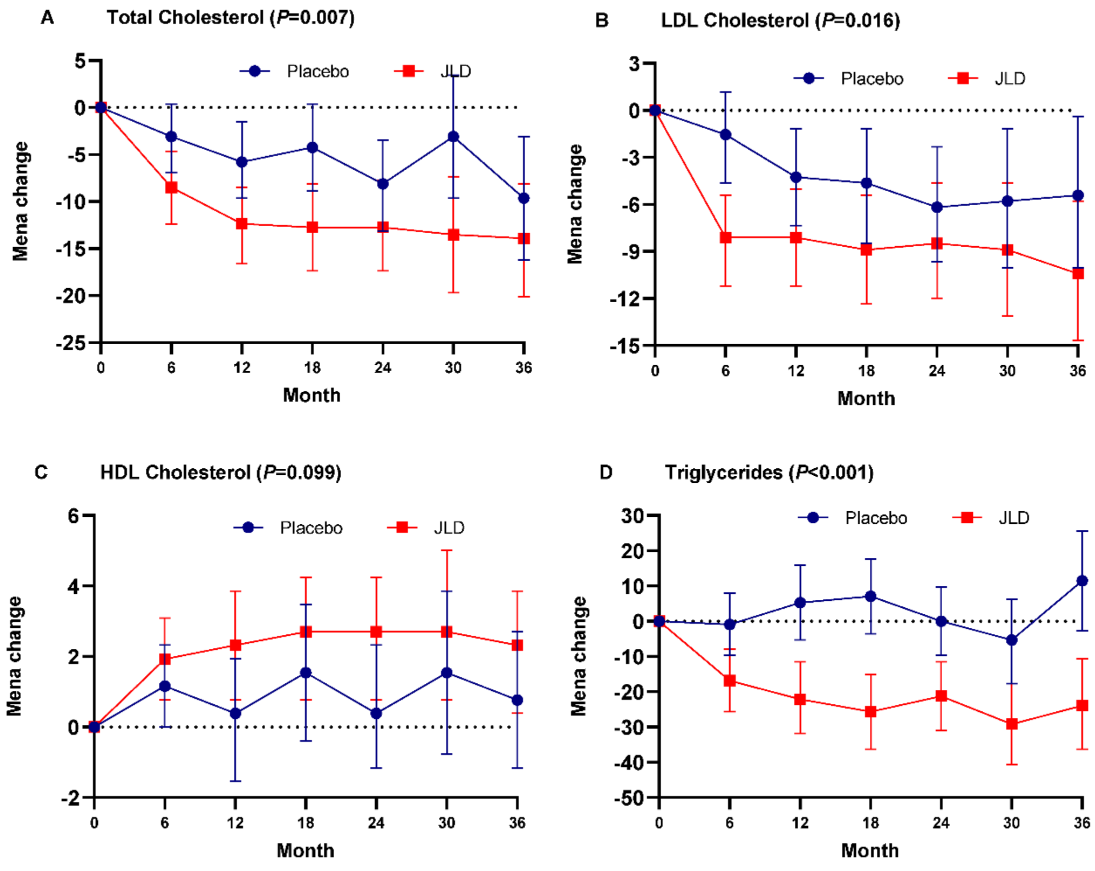

Note: (**A-D**) Effect of JLD on Total Cholesterol, LDL Cholesterol, HDL Cholesterol, Triglycerides. During the study, the mean change and 95% confidence interval (CI) for continuous measurements were calculated using mixed-effects regression models (MMRM). The models were fit to all available data for each measurement. P-values assessed the significance of between-group differences in mean changes, with error bars representing the 95% CI bounds.

© 2024 Ji H et al. *JAMA Internal Medicine.*

eFigure 3. Mean changes of blood pressure in the JLD and placebo

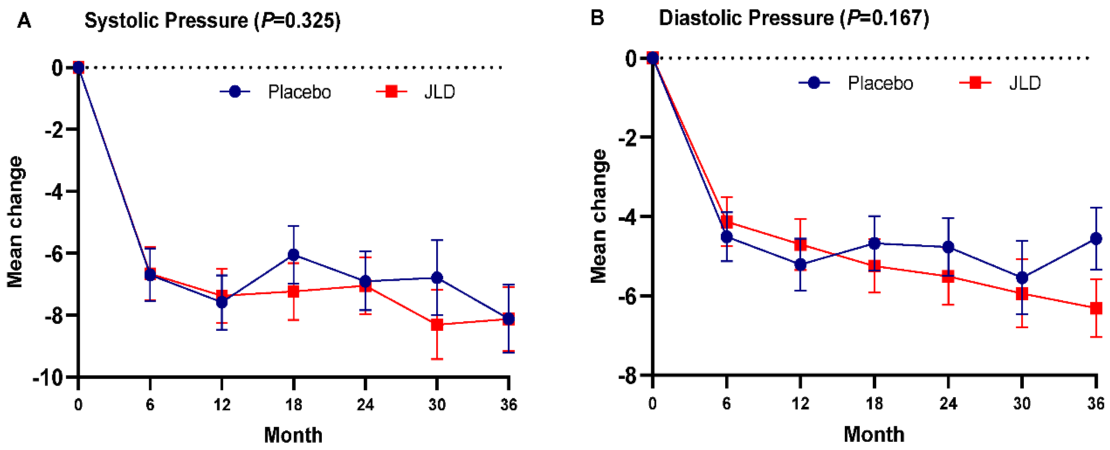

Note: During the study, the mean change and 95% confidence interval (CI) for continuous measurements were calculated using mixed-effects regression models (MMRM). The models were fit to all available data for each measurement. P-values assessed the significance of between-group differences in mean changes, with error bars representing the 95% CI bounds.

© 2024 Ji H et al. *JAMA Internal Medicine.*

eFigure 4. Effects of JLD on HOMA-IR

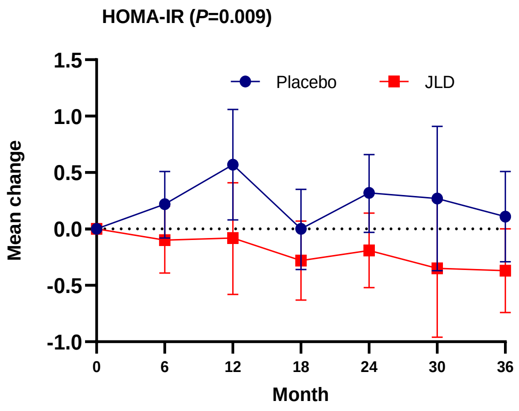

Note: (**A-D**) Effect of JLD on Total Cholesterol, LDL Cholesterol, Triglycerides; HOMA-IR. During the study, the mean change and 95% confidence interval (CI) for continuous measurements were calculated using mixed-effects regression models (MMRM). The models were fit to all available data for each measurement. P-values assessed the significance of between-group differences in mean changes, with error bars representing the 95% CI bounds.

© 2024 Ji H et al. *JAMA Internal Medicine.*

eFigure 5. The herb-compound-target network of JLD regulating insulin resistance pathway

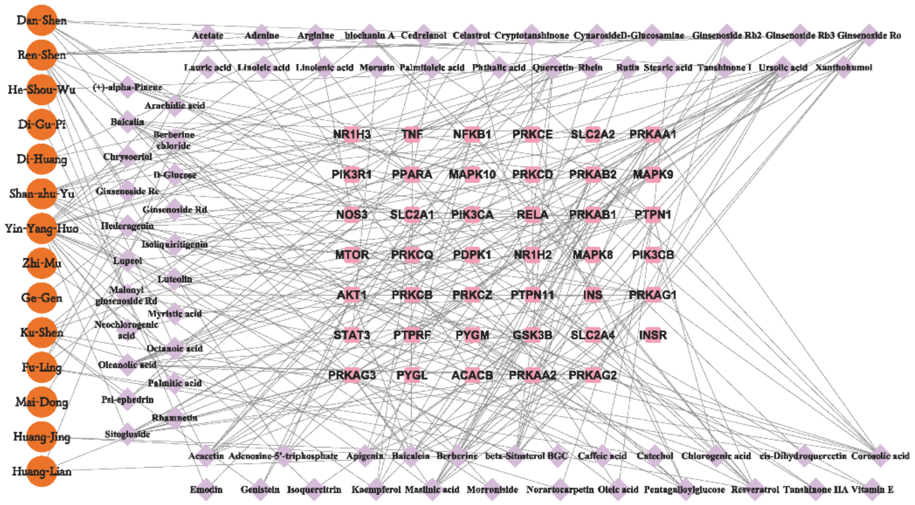

Note: Yellow brown circular nodes represent the herbs in JLD, purple diamond nodes represent the components in JLD, and the pink square nodes are the targets on the insulin resistance pathway. The JLD prescription consists of seventeen herbs: one monarch drug (JUN)- the *Panax ginseng C. A. Mey.* (Ren-Shen), three ministerial drug (CHEN)- *Polygonatum sibiricum.* (Huang-Jing), *Atractylodes species.* (Cang-Zhu), *Sophora flavescens Alt.* (Ku-Shen), eleven adjuvant drug (ZUO)- *Salvia miltiorrhiza Bge.* (Dan-Shen), *Coptis chinensis Franch.* (Huang-Lian), *Cornus officinalis Sieb.et Zucc.* (Shan-zhu-Yu), *Tuber of dwarf lilyturf.* (Mai-Dong), *Rehmannia glutinosa.* (Sheng-Di-Huang), *Poria cocos.* (Fu-Ling), *Fallopia multiflora.* (He-Shou-Wu), *Eupatorium fortune.* (Pei-Lan), *Cortex Lycii.* (Di-Gu-Pi), *Anemarrhena asphodeloides.* (Zhi-Mu) and *Herba Epimedii.* (Yin-Yang-Huo), two envoy drug (SHI)- *Puerariae Lobatae Radix.* (Ge-Gen), *Semen Litchi.* (Li-Zhi-He). JLD is orally administered as granule. The picture of this patent medicine has been permitted to be presented in the manuscript by YILING PHARMACEUTICAL, INC.

© 2024 Ji H et al. *JAMA Internal Medicine.*

eTable 1. Sensitivity analysis of diabetes incidence

Index	PPS		Complete the trial	
	**JLD**	**Placebo**	**JLD**	**Placebo**
N	439	440	388	390
Diabetes incidence（%）	122(27.79)	187(42.50)	123(31.70)	189(48.46)
Censoring（%）	317(72.21)	253(57.50)	265(68.30)	201(51.54)
Logrank test	18.102		20.998	
P value	<0.001		<0.001	
Cox regression				
Hazard Ratio	0.586		0.557	
95%CI	0.465,0.740		0.442,0.703	
Wald value	20.255		24.319	
P value	<0.001		<0.001	

© 2024 Ji H et al. *JAMA Internal Medicine.*

eTable 2. Adverse events

	JLD (n=443)		Placebo (n=446)	
	Participants	Events	Participants	Events
Any adverse events	420 (94.81%)	2321	410 (91.93%)	2233
Serious adverse events	20 (4.51%)	25	18 (4.04%)	21
Adverse events leading to trial product discontinuation	4 (0.90%)	4	4 (0.90%)	4
Fatal events	1 (0.23%)	1	0 (%)	0
Adverse events reported in at least 10% of patients				
Cough	143 (32.28%)	172	130 (29.15%)	152
Nasopharyngits	136 (30.70%)	208	133 (29.82%)	202
Pharyngalgia	104 (23.48%)	117	112 (25.11%)	123
Dizziness	103 (23.25%)	110	108 (24.22%)	119
Headache	84 (18.96%)	92	77 (17.26%)	83
Coronavirus infections	71 (16.03%)	71	56 (12.56%)	56
Diarrhoea	69 (15.58%)	76	74 (16.59%)	87
Upper respiratory tract infection	68 (15.35%)	80	70 (15.70%)	82
Rhinorrhea	66 (14.90%)	81	61 (13.68%)	77
Nausea	53 (11.96%)	54	44 (9.87%)	49
Hyperuricemia	51 (11.51%)	52	53 (11.88%)	53
Nasal congestion	47 (10.61%)	48	51 (11.43%)	52
Safety areas of interest				
Gastrointestinal disorders	197 (44.47%)	406	199 (44.62%)	387
Hepatic disorders	37 (8.35%)	37	32 (7.18%)	32
Acute renal failure	0	0	0	0
Cardiovascular events	28 (6.32%)	29	20 (4.48%)	22
Allergic reactions	0	0	0	0
Malignant neoplasms	3 (0.68%)	4	1 (0.22%)	1
Psychiatric disorders	49 (11.06%)	53	33 (7.40%)	35
Hypoglycaemia	1 (0.23%)	1	1 (0.22%)	1

Notes: Data are n (%) of the safety analysis population (all randomly allocated participants exposed to at least one dose of intervention) experiencing at least one event. Data are for on-treatment adverse events occurring during treatment. Hepatic disorders are defined as serum transaminase higher than the upper limit of normal value. JLD group: one death due to suicide in patient with depression. The

© 2024 Ji H et al. *JAMA Internal Medicine.*

patient was diagnosed with depression 5 months after randomization and hospitalized for treatment many times. One and a half year after randomization, the patient committed suicide. Most common adverse events reported in at least 10% of patients in either group. Events confirmed by event adjudication committee.

© 2024 Ji H et al. *JAMA Internal Medicine.*

eMethods. Methodologic appendix

1) The formulation of the research protocol: The clinical research protocol was jointly developed through extensive discussions among clinical experts and statisticians organized by the principal investigators (Fengmei Lian, Xiaolin Tong, Zhenhua Jia). After finalization, the protocol was registered with the China Clinical Trial Registry (www.ChiCTR.org.cn) (ID: ChiCTR1900023241) and published following the commencement of the experiment (doi:10.3389/fendo.2020.00415. Accepted: 26 May 2020; Published: 25 June 2020), ensuring consistency between the actual clinical implementation and the published protocol. Therefore, the objectivity of the clinical protocol's execution was guaranteed, and the possibility of interference in the execution of the protocol was eliminated.
2) Randomization Implementation and Statistical Analysis: The randomization protocol, subject Blind, drug Blinding, drug coding, and statistical analyses were all independently conducted by a third-party statistical entity. The implementation of randomization utilized a central randomization system, where both randomization blind codes and drug blind codes were stored. The database was locked, and the statistical analysis plan was finalized before unblinding in the central randomization system. This entire process rigorously ensured the implementation of the clinical trial's randomized double-blind design.
3) An independent third-party Contract Research Organization (CRO) is responsible for the organization and implementation of the clinical trial, data collection, and quality control, ensuring the trial's independence, authenticity, accuracy, and completeness.
4) This study has formed the Data Safety Monitoring Board (DSMB) and a Clinical Endpoint Adjudication Committee to independently adjudicate safety data and clinical endpoint events (occurrence of diabetes).

© 2024 Ji H et al. *JAMA Internal Medicine.*

eAppendix: Detailed information of Jinlida granules and placebos

1. Information of Jinlida granules

1.1. For the details about the composition, dosage, efficacy, safety, and quality control of the formula

Composition (Additional material 1)	Components	Name in Chinese	Species	Medicinal Parts
	Ginseng radix et rhizoma	Ren-Shen	Panax ginseng C. A. Mey	Roots and Rhizomes
	Polygonati rhizoma	Huang-Jing	Polygonatum sibiricum	Rhizomes
	Atractylodis rhizoma (stir-baked with bran)	Cang-Zhu（Fu Chao）	Atractylodes species	Rhizomes
	Sophorae flavescentis radix	Ku-Shen	Sophora flavescens Ait	Roots
	Puerariae thomsonii radix	Fen-Ge	Pueraria thomsonii Benth.	Roots
	Litchi semen	Li-Zhi-He	Litchi chinensis Sonn.	Seeds
	Anemarrhenae rhizoma	Zhi-Mu	Anemarrhena asphodgfoides	Rhizomes
	Epimedii folium (stir-baked)	Yin-Yang-Huo（Zhi）	Herba epimedil	Leaves
	Corni fructus	Shan-Zhu-Yu	Cornus officinalis Sieb. et Zucc	Sarcocarp
	Coptidis rhizoma	Huang-Lian	Coptis chinensis Franch.	Rhizomes
	Salviae miltiorrhizae radix et rhizoma	Dan-Shen	Salvia miltiorrhiza Bge.	Roots and Rhizomes
	Ophiopogonis radix	Mai-Dong	Ophiopogon japonicus	Tubers
	Rehmanniae radix	Di-Huang	Rehmanniag glutinosa	Tubers
	Poria	Fu-Ling	Poria cocos	Sclerotia
	Polygoni multiflori radix praeparata	He-Shou-Wu（Zhi）	Polygonum multiflorum Thunb.	Tubers
	Eupatorii herba	Pei-Lan	Eupatorium fortunei Turcz.	Aboveground parts
	Lycii cortex	Di-Gu-Pi	Cortex Lycii	Velamina

© 2024 Ji H et al. *JAMA Internal Medicine.*

**Dosage** (Additional material 1)	Take the medicine orally after mixing it with hot water, 1 pack per time, three times a day for 8 weeks as one treatment course, or as advised by health professionals. The use of this product can be combined with western medicine, and appropriate dose reduction of the latter should be adjusted according to the blood glucose level.
**Efficacy** (Additional material 1)	**Actions:** To *tonify qi*, *nourish yin*, *fortify the spleen* and *transport fluid*.<br>**Indications:** Type 2 diabetes due to dual deficiency of qi and yin, manifested as thirst, increased water and food intake, increased appetite, increased urination, emaciation, fatigue, lack of strength, spontaneous sweating, night sweating, vexing heat in the chest, palms and soles, and constipation, etc.
**Safety** (Additional material 2)	Post-marketing monitoring data show that this product may cause gastrointestinal adverse reactions such as diarrhea, nausea, and rash, itching.
**Quality Control** (Additional material 1)	Comply with the monograph of "Jinlida Granules" in Chinese Pharmacopoeia 2020, Volume I, Page 1145-1147.

1.2. Details of the Jinlida formula

1) The proprietary product name (i.e., brand name)

Jinlida Granules

2) Name of manufacturer

Manufacturer: Shijiazhuang Yiling Pharmaceutical Co., Ltd.

Address: No.238 Tianshan street, High-Tech Area, Shijiazhuang, Hebei, China.

3) Lot number, production date, expiry date

Lot number	Production date	Expiry date
A1810001	Oct 28, 2018	Sep 2021
A1810002	Oct 29, 2018	Sep 2021
2007001	Jul 19, 2020	Jun 2023
2112001	Dec 10, 2021	Nov 2024

4) Certificate of Analysis for Finished Product (Additional material 3)

5) UPLC fingerprint for ten different batches of Jinlida granules and their reference (Additional material 4)

6) Name and percentage of added materials

Lactose Monohydrate: 50%

Dextrin:10%

7) Whether any additional quality control measures were conducted

No.

1.3. Statement of whether the patent proprietary formula used in the trial is for a condition that is identical to the publicly available reference

Proprietary formula (Jinlida granules) used in the experiment are different from the publicly available reference. Jinlida granules was approved by National Medical Products Administration (NMPA) in 2005 for treating type 2 diabetes mellitus (T2DM). In the trial, Jinlida granules was used to intervene in patients with impaired glucose tolerance and multiple metabolic abnormality.

2. Information of Placebo (Additional material 5)

2.1. Name and amount of each ingredient

Malt Extract: 17.81%

Lactose Monohydrate: 68.17%

Dextrin: 13.63%%

Tartrazine: 0.02%

Caramel pigment: 0.37%

2.2. Description of the similarity of placebo with Jinlida granules

After comparison, the placebo and Jinlida granules are consistent in terms of color, smell, taste, appearance, packaging.

2.3. Quality control and safety assessment, if any

No.

2.4. Administration route, regimen, and dosage

Take the medicine orally after mixing it with hot water, 1 pack per time, three times a day.

2.5. Production information: where, when, how, and by whom the placebo was produced

Production date: Oct 28, 2018; Oct 29, 2018; Jul 19, 2020; Dec 10, 2021

Manufacturer: Shijiazhuang Yiling Pharmaceutical Co., Ltd.

Address: No.238 Tianshan street, High-Tech Area, Shijiazhuang, Hebei, China.

2.6. Components of Jinlida granules Placebo and Evaluation Methods

Evaluation Methods:

1）The visual, taste, and olfactory indexes of Jinlida placebo and Jinlida investigational drug are measured independently by visual, taste and olfactory sensors and 10 people’s experiences, and the similarity between them is calculated. For various indexes, the overall scores, objective and quantified scores and human experience sensory scores are given; when the average scores are all greater than 80%, the products are qualified.

2）The weights of visual, taste and olfactory indexes are 40%, 40%, and 20%, respectively.

3）A total of five groups (groups A, B, C, D and E) of Jinlida placebos are prepared, and compared with Jinlida investigational drugs, 11 aliquots for each group, and 20 g each aliquot.

Evaluation Results:

© 2024 Ji H et al. *JAMA Internal Medicine.*

Independent assessment is performed based on the above criteria. The objective, quantitative similarities are as follows: C: 95%, A: 89%, D: 82%, B: 78%, E: 70%. The average sensory scores based on human experiences are as follows: A: 93%, C: 89%, D: 83%, B: 68%, E: 61%.

Note: If none of the five groups meet the conditions, the manufacturing process of placebo will be adjusted, and placebos will be remanufactured until compliance with requirements.

Summary: Through the above evaluation, the placebo C is used and the placebo A is for standby in this study.

© 2024 Ji H et al. *JAMA Internal Medicine.*
