# Supplementary material for: Jinlida for Diabetes Prevention in Impaired Glucose Tolerance and Multiple Metabolic Abnormalities: The FOCUS Randomized Clinical Trial
Source: JAMA Intern Med. 2024 Jun 3;184(7):727–35. doi: 10.1001/jamainternmed.2024.1190 (PMC11148787; doi:10.1001/jamainternmed.2024.1190)
Supplement: Supplement 2. — Trial Protocol and Statistical Analysis Plan [file jamainternmed-e241190-s002.pdf]

The content of this research protocol is confidential and is intended for use by clinical researchers. This material is the property of the funder or its sub-units and may not be copied or distributed to individuals unrelated to this project.

Interventional study of Jinlida for treatment of abnormal glucose metabolism in metabolic syndrome （JLD-FOCUS）

Study Protocol

Protocol Number: SP-YFC-01-JLD-FOCUS

Version Number: 1.0

Version Date: April 10,2019

ChiCTR Registration Number: ChiCTR1900023241

Sponsor-Unit: Hebei Yiling Hospital

Sponsor-Investigator: Professor Jia Zhenhua

Study Period: March 2019 to December 2022

Sponsor and Principal Investigator

**Sponsor**	**Hebei Yiling Hospital**	
**Principal Investigator**	Jia Zhenhua<br>signature:	
**Clinical Research Coordinator**	Guang'anmen Hospital	Professor Lian Fengmei
	Hebei Yiling Hospital	Professor Gao Huailin

Academic Counselors

**Member**	Affilitation
Gao Runlin	Chinese Cardiovascular Center, Chinese Academy of Medical Sciences, Fuwai Hospital
Zhang Boli	China Academy of Chinese Medical Sciences
Han Yaling	Shenyang Military Region General Hospital of the People's Liberation Army, Institute of Cardiovascular Diseases
Ge Junbo	Zhongshan Hospital affiliated to Fudan University
Mei Wang	Leiden University in the Netherlands
Tong Xiaolin	Guang'anmen Hospital, China Academy of Chinese Medical Sciences

Academic Advisory Committee

**Member**	Affilitation
Zhu Dalong	Nanjing Drum Tower Hospital, the Affiliated Hospital of Nanjing University Medical School
Wu Shentao	The First Affiliated Hospital of Tianjin University of Traditional Chinese Medicine
Song Guangyao	Hebei General Hospital
Lu Wei	Shuguang Hospital affiliated to Shanghai University of Traditional Chinese Medicine
Lian Fengmei	Guang'anmen Hospital, China Academy of Chinese Medical Sciences
Li Xiaoying	Zhongshan Hospital, Fudan University
Kuan Hongyu	The First Affiliated Hospital of Harbin Medical University

Endpoint Events Review Committee

Member	Affiliation
Song Guangyao	Hebei General Hospital
Lian Fengmei	Guang'anmen Hospital, China Academy of Chinese Medical Sciences
Chen Liming	NHC Key Laboratory of Hormones and Development, Chu Hsien-I Memorial Hospital and 18 Tianjin Institute of Endocrinology, Tianjin Medical University

Data Safety and Monitoring Board

Member	Affiliation
Qingshan Zheng	Shanghai University of Traditional Chinese Medicine
Xinhua Xiao	Peking Union Medical College Hospital
Li Yan	Sun Yat-Sen Medical Hospital

Third-Party Statistician

Statistical Affilitation	Principal
Peking University Clinical Research Institute	Yao Chen

Drug Provider

Drug Provider	Principal
Shijiazhuang Yiling Pharmaceutical Co., Ltd.	Han Shuolong

Research Affilitation and Principal Investigator

The sites are dynamically adjusted, therefore, in the main manuscript, 35 sub-centers were ultimately identified.

	**Sponsor Investigator**	
	Jia Zhenhua signature:	
	**Sponsor**	**Principal Investigator**
1	Hebei Yiling Hospital	Gao Huailin
	**Collaborator**	
2	Hebei General Hospital	Song Guangyao
3	Guang'anmen Hospital, China Academy of Chinese Medical Science Sciences	Lian Fengmei
4	Shenzhen Hospital, Guangzhou University of Traditional Chinese Medicine	Piao Chunli
5	Shuguang Hospital affiliated to Shanghai University of Traditional Chinese Medicine	Lu Hao
6	Shandong Provincial Hospital	Zhao Jiajun
7	Nanjing Drum Tower Hospital, the Affiliated Hospital of Nanjing	Xu Tianshu
8	Beijing Union Medical College Hospital	Xiao Xinhua
9	The First Affiliated Hospital of Anhui University of Traditional Chinese Medicine	Fang Zhaohui
10	Affiliated Hospital of Liaoning University of Traditional Chinese Medicine	Wang Magnesium
11	The First Affiliated Hospital of Harbin Medical University	Kuang Hongyu
12	Inner Mongolia Autonomous Region People's Hospital	Chen Yu
13	The Second Affiliated Hospital of Yunnan College of Traditional Chinese Medicine	Fan Yuan

14	The Second Affiliated Hospital of Nanchang University	Lai Xiaoyang
15	Tangshan Workers Hospital	Fang Hui
16	Tianjin General Hospital	Zhu Tiehong
17	The Sixth Hospital of Sun Yat-sen University	Zhao Tongfeng
18	First Affiliated Hospital of Anhui Medical University	Zhang Qiu
19	Qilu Hospital of Shandong University	Chen Li
20	Affiliated Hospital of Qingdao University Medical College	Wang Yangang
21	Shijiazhuang Diabetes Hospital	Bitg
22	First Affiliated Hospital of Zhengzhou University	Qin Guijun

Abstract - 8 -
Abbreviations - 10 -
Research project description - 11 -
1. Research title - 11 -
2. Background - 11 -
2.1 Metabolic syndrome and cardiovascular and cerebrovascular diseases - 11 -
2.2 Metabolic syndrome and diabetes - 12 -
2.3 Current status of treatment - 13 -
2.4 Traditional Chinese medicine and Jinlida granules - 13 -
3. Trial objectives - 14 -
4. Study design - 15 -
4.1 Sample size calculation - 15 -
4.2 Randomization - 15 -
5. Subjects **Error! Bookmark not defined.**
5.1 Prediabetes diagnostic criteria - 15 -
5.2 Diagnostic criteria for type 2 diabetes - 16 -
5.3 Diagnostic criteria for metabolic syndrome - 16 -
5.4 Inclusion criteria - 16 -
5.5 Exclusion criteria - 17 -
5.6 Elimination criteria - 17 -
5.7 Drop-out criteria - 18 -
5.8 Termination criteria for subjects - 18 -
5.9 Termination criteria for overall-trial - 19 -
6. Intervention - 19 -
6.1 Treatment - 19 -
6.2 General lifestyle intervention - 19 -
6.3 Dietary intervention - 20 -
6.4 Exercise intervention - 22 -
6.5 Quit smoking - 22 -
6.6 Intervention method and follow-up - 23 -
6.7 Combined medication and combined treatment - 23 -
6.8 Research drug - 23 -
6.8.1 Preparation, packaging and labeling - 24 -
6.8.2 Preservation method - 24 -
6.8.3 Drug dispensing and recycling - 24 -
6.8.4 Drug inventory - 24 -
6.8.5 Drug management - 25 -
7. Study program - 25 -
8. Treatment period - 26 -
9. Compliance evaluation - 26 -
10. Efficacy indicator - 26 -
10.1 Primary efficacy indicator - 26 -
10.2 Secondary efficacy indicator - 26 -
10.3 Safety indicator - 26 -
11. Research process - 27 -
11.1 Screening period (-37 days - 1 month) - 27 -
11.2 Run-in period (-1 month - day 0) - 27 -
11.3 Entry period - 27 -
11.4 Randomized treatment period 1 - 28 -
11.5 Randomized treatment period 2 - 28 -
11.6 Randomized treatment period 3 - 29 -
11.7 Randomized treatment period 4 - 29 -
11.8 Last follow-up - 30 -
11.9 Tracking visits: (required)* - 30 -
12. Defintion of endpoint events and their judgment - 30 -
13. Observation of adverse events - 30 -

13.1 Definition - 30 -
13.2 Classification standards of adverse events - 31 -
13.3 Judging criteria for relationship between adverse events and research drugs - 31 -
13.4 Judgment of serious adverse events - 32 -
13.4.1 Definition of general serious adverse events - 32 -
13.4.2 Operating procedures - 32 -
13.4.3 Measures of serious adverse event management - 33 -
13.5 AE/SAE ending/transition - 34 -
13.6 Abnormal laboratory results - 34 -
14. Blinding method - 35 -
14.1 Emergency letter - 35 -
14.2 Emergency unblinding - 35 -
14.3 Unblinding regulations - 35 -
15. Data management - 36 -
15.1 Completion and transfer the case report form - 36 -
15.2 Data entry and modification - 36 -
15.3 Blindness review and unblinding - 36 -
16. Statistical Analysis - 37 -
16.1 Analysis subjects - 37 -
16.2 Statistical analysis method - 37 -
16.3 Enrollment and completion - 38 -
16.4 Compliance analysis - 38 -
16.5 Efficacy evaluation - 38 -
16.6 Safety evaluation - 38 -
16.7 Interim analysis - 38 -
17. Quality Control - 39 -
17.1 Improvement measures of observation consistency - 39 -
17.2 Laboratory quality control requirements - 40 -
17.3 Measures to ensure subject compliance - 40 -
18. Ethical considertions - 40 -
19. Research progress - 41 -
20. Data reservation - 41 -
21. References - 41 -
Apendix 1: Standardized operating procedures for blood pressure measurement - 45 -
Apendix 2: Common Physical Fitness Indicators - 46 -
Research flow chart - 47 -

Abstract

**Research title**	Interventional study of Jinlida treatment for abnormal glucose metabolism in metabolic syndrome
**Trial objectives**	To evaluate the effectiveness of Jinlida in delaying or inhibiting the development of diabetes mellitus in metabolic syndrome subjects with impaired glucose tolerance. To provide clinical evidence for high-risk factors requiring intervention to prevent arteriosclerosis and inhibit the development of the cardiovascular pathology.
**Study design**	Randomized, double-blind, placebo-controlled, multicenter clinical study
**Inclusion and exclusion criteria**	**Inclusion criteria:**<br>1. Subjects aged between from 18 toand 70 years old;<br>2. After the run-in period, subjects should meet the diagnostic criteria for metabolic syndrome;<br>3. After the run-in period, subjects should meet the diagnostic criteria for impaired glucose tolerance;<br>4. Subjects who have signed the informed consent form.<br>**Exclusion criteria:**<br>1. Subjects who have used hypoglycemic drugs in the past 3 months;<br>2. Subjects with type 1 or type 2 diabetes (T2D), gestational diabetes, secondary diabetes and other special types of diabetes;<br>3. Hyperthyroidism or hypothyroidism caused by endocrine disorders;<br>4. Uncontrollable hypertension/hypotension: systolic blood pressure ≥200mmHg or diastolic blood pressure ≥110mmHg; or systolic blood pressure ≤90mmHg or diastolic blood pressure ≤60mmHg;<br>5. Comorbidities such as various acute infections, or severe infections, severe anemia and neutropenia;<br>6. Major disease comorbidities, such as active or untreated malignant tumors, or clinical remission of malignant tumors less than 5 years ago;<br>7. Natural heart disease, such as congenital heart disease, rheumatic heart disease, hypertrophic or dilated cardiomyopathy, NYHA cardiac function classification > Ⅲ level;<br>8. Subjects who had any of the following conditions within the past 6 months: coronary intervention (eg: CABG or PTCA), stroke (including ischemic and hemorrhagic stroke);<br>9. Severe liver and kidney dysfunction (ALT three times greater than the upper limit of the normal and creatinine greater than 132 μmol / l);<br>10. After the run-in period, fasting TG is less than 5.6mmol/L;

	11. Pregnant or lactating women, and women of childbearing age who have not taken effective contraceptive measures;<br>12. Participation in any other clinical trials;<br>13. For any reason, the investigator may consider a subjects inappropriate for participation in this study.	
**Termination criteria**	1. An allergic reaction that is clearly associated with the study drug;<br>2. Adverse symptoms or signs and abnormal examination results occur that are clearly related to intake of the study drug;<br>3. Women who develop pregnancy during the study;<br>4. Subjects request to withdraw from the study;<br>5. Signs of severe or frequent hypoglycemia: hypoglycemic events that require help from others (eg: increased physical activity or no meals), or weekly hypoglycemic events (plasma or hand-pricked blood glucose measurements ≤70 mg/dL [≤ 3.9mmol/L]; any episode with or without symptoms) occurring more than three times;<br>6. According to the results of OGTT screening, the patient was diagnosed as type 2 diabetes, and retesting one week later, OGTT was still diagnosed type 2 diabetes;<br>7. Cardiovascular or cerebrovascular events that occur during the study including: acute myocardial infarction, stroke, hospitalization for heart failure, etc.	
**Effectiveness assessment**	Primary outcomes	Incidence of type 2 diabetes
	Secondary outcomes	1. Changes in the number of components of the metabolic syndrome；<br>2. Changes in single indicators (waist circumference, blood pressure, TG, HDL-C) in metabolic syndrome;<br>3. Carotid intima-media thickness (IMT);<br>4. Fasting insulin;<br>5. Ankle brachial index;<br>6. Glycated hemoglobin (HbA1c);<br>7. Endothelial function test: Serum NO, ET-1;<br>8. C-reactive protein;<br>9. Urinary microalbumin to creatinine ratio (ACR).
**Safety assessment**	Routine blood and urine examination, liver and kidney function, electrocardiogram, recording adverse events, physical examination, etc.	

**Sample size**	The total number of cases will be 880. The ratio of subjects between the treatment group and the placebo group is designed to be 1:1.
**Intervention**	Study group: General lifestyle intervention + Jinlida granules, 9g / bag, 1 bag / time, 3 times / day;<br>Control group: General lifestyle intervention + placebo, 9g / bag, 1 bag / time, 3 times / day
**Study period**	The run-in period is one month and the intervention, with follow-up, will last 24 to 48 months.
**Statistical analysis**	Peking University Clinical Research Institute
**Expected progress**	Statistical analysis will be completed in December 2021

Abbreviations

ADA	American Diabetes Association
ADR	Adverse Dug Reaction
AE	Adverse Event
ALT	Alanine Aminotransferase
ASCVD	Atherosclerotic Cardiovascular Disease
ATP Ⅲ	Third report of the adult treatment group
BMI	Body Mass Index
CABG	Coronary Artery Bypass Grafting
CDS	Chinese Diabetes Society
CRF	Case Report Form
CVD	Cardiovascular Disease
DM	Diabetes
FAS	Full Analysis Set
HDL-C	High Density Lipoprotein
IDF	International Diabetes Federation
IFG	Impaired Fasting Glucose
IGT	Impaired Glucose Tolerance
MS	Metabolic Syndrome
NCEP	National Cholesterol Education Program
NGT	Normal Glucose Tolerance
OGTT	Oral Glucose Tolerance Test
PPS	Per Protocol Set

PTCA	Percutaneous Transmittal Coronary Angioplasty
SAE	Severe Adverse Event
SS	Security Data Set
T2DM	Type 2 Diabetes
TG	Triglyceride
WHR	Waist-Hip Ratio

Research project text

1 Research title

Interventional study of Jinlida for treatment of abnormal glucose metabolism in metabolic syndrome.

2 Background

2.1 Metabolic syndrome and cerebral-cardio vascular diseases

In the 21st century, disease incidence has quietly changed worldwide. The incidence of acute infectious diseases has gradually declined while the incidence of chronic metabolic diseases is increasing year by year. Metabolic syndrome (MS) is a typical chronic metabolism disorder which has garnered much attention among heath-care professionals[1]. Metabolic syndrome applies to a group of subjects with obesity, hyperglycemia (including diabetes or pre-diabetes), dyslipidemia (high TG and/or low HDL-C) and high blood pressure; all of which have a serious negative affect on a patient’s health. Meanwhile, MS is a group of interrelated risk factors that directly contribute to the development of atherosclerosis cardiovascular disease (ASCVD) and increase the risk of developing type 2 diabetes[2].

Research has shown that the risk of cardiovascular and cerebrovascular diseases (coronary heart disease and stroke) is increased three times, the risk of diabetes is increased five-fold and the risk of cardiovascular or cerebrovascular death is doubled, compared to people without non-metabolic syndrome. The total risk of death is increased by a factor of 1.5compared with non-metabolic syndrome.[3] The results of a cohort study surveying 11 provinces of China demonstrated that the incidence of cardiovascular and cerebrovascular diseases in subjects with metabolic syndrome was 3.12 times greater than those without such a condition[4].

Currently, the prevalence of metabolic syndrome is also increasing. According to data published by the International Diabetes Federation (IDF) in 2005, a quarter of the world's population had metabolic syndrome[5-6]. Accoding to CHNS in 2002, the morbidity rates of Chinese adults aged over 18 years who met the Chinese Medical Association Diabetes Association (CDS) and the American Cholesterol Education Program (NCEP) in adult treatment group in the third report (ATP

Ⅲ) with metabolic syndrome diagnostic criteria were 6.6% and 13.8%, respectively. According to a 2010 report from China Chronic Disease Surveillance, the prevalence of metabolic syndrome in accordance with the diagnostic criteria for NCEP-ATPⅢ among 98,658 adults aged >18 years in 31 provinces was 33.9%. This was a significantly higher prevalence than in 2002. As the global urbanization process accelerates, the population of metabolic syndrome further expands.

2.2 Metabolic syndrome and diabetes

Studies have shown that 86% of diabetic subjects, 71% with impaired fasting glucose (IFG) and 33% with impaired glucose tolerance (IGF) also suffered from metabolic syndrome[7]. The "Guidelines for the Prevention and Treatment of Type 2 Diabetes in China (2017 Edition)" states that type 2 diabetics with metabolic syndrome often have many clinical manifestations such as hypertension, dyslipidemia, and obesity. With the increasing levels of blood glucose, blood pressure, blood lipids and body weight, the risk of developing of type 2 diabetes complications will skyrocket.

As China's population is aging and their lifestyles change, diabetes has become an epidemic and the prevalence of diabetes has mushroomed from 0.67% in 1980 to 10.4% in 2013[2]. Further, diabetes is an independent risk factor for cardiac-cerebrovascular disease[2]. Compared with non-diabetic people, the risk of cardiac-cerebrovascular disease in diabetic subjects has increased 2 to 4 times [6]. As levels of fasting blood glucose and 2h postprandial blood glucose rise, even if the diagnostic criteria for diabetes (eg: pre-diabetes) is not reached, the risk of cardiac-cerebrovascular disease is significantly increased [8-10]. Most people with diabetes have experienced the “pre-diabetes” stage[11-12], a state in which there is a high risk of developing diabetes and become potential risk factor for diabetes[13] and cardiovascular disease[14-15]. Pre-diabetes is an intermediate metabolic state between normal blood glucose status (NGT) and diabetes (DM), including impaired fasting glucose (IFG) and impaired glucose tolerance (IGT).

Ntional research showed that the incidence of pre-diabetes among adults aged >20 years was 15.5% in about 148.2 million adults[16]. According to predication of the American Diabetes Association (ADA), up to 70% of pre-diabetes subjects eventually will develop diabetes[15]. If there is effective intervention during the pre-diabetes stage, glucose levels may reverse to normal glucose metabolism[17-21]. If left untreated it may develop into diabetes with macrovascular and microvascular comorbidities[2]. IGT is more common than IGF and so the risk of developing diabetes and cardiovascular disease in the IGT population is significantly higher than that of the IFG population[22]. In the absence of effective intervention, approximately 90% of IGT subjects develop type 2 diabetes within 20 years, and half of them have at least one myocardial infarction or stroke event[23]. IGT subjects also have with other risk factors of CVD such as hypertension, dyslipidemia, obesity and other metabolic diseases that constitute metabolic syndrome; these have been deemed to be an important risk factor for cardiovascular and cerebrovascular diseases[24].

Subjects with metabolic syndrome and diabetes have more than 1.5-2.5 times higher risk of coronary heart disease than those with metabolic syndrome or diabetic heart disease alone[7]. The

prevention and treatment of metabolic syndrome with diabetes is of urgent importance. Therefore, preventing or delaying the progression of abnormal glucose metabolism into diabetes should effectively reduce the incidence and thus mortality rate of cardiovascular and cerebrovascular diseases as well as diabetes.

2.3 Current status of treatment

The pathogenesis of metabolic syndrome and diabetes is not well understood[25-26]. Its occurrence and development may be related to genetic factors[24], unhealthy eating habits as well as lack of exercise[27-29]. Active long-term diet control and exercise interventions have been shown to be an important measure to achieve these goals[15]. However whether or not lifestyle interventions can be effectively be implemented in clinical practice remain a shortcoming of current therapy[30]. At present, targeted drugs for treating metabolic syndrome have not yet been developed. In clinical practice, Western medicine mainly focuses on MS components. Such drug targets are mainly suitable for subjects with a poor effectiveness of lifestyle interventions or subjects with high risk factors of cardiovascular and cerebrovascular diseases.

The main purpose of therapy is to reduce various risk factors and cardiovascular and cerebrovascular events as well as diabetes[31] through an intervention on MS components. However, drug efficacy and safety need to be further developed and assessed clinically, which is a heavy psychological and economical burden on both subjects and the medical establishment.

2.4 Traditional Chinese Medicine and Jinlida granules

Traditional Chinese medicine (TCM) emphasizes holistic health care. MS is conceptualized as a state of imbalance in human function by TCM, which change our understanding of the diseases from controlling of a single hypoglycemic, antihypertensive, and lipid-regulating to holistic regulation. This medical modality uses philosophical thinking such as mutual use, mutual transformation, homotherapy for heteropathy and treating the same disease with different methods. TCM has a rich history as part of long-term medical practice preventing and treating MS as well as diabetes. TCM has certain advantages and great potential for the prevention and treatment of MS[31]. However, its efficacy has not been objectively evaluated.

Thinking about the efficacy of Jinlida granules is guided by the theory of collateral disease, targeting the etiology and pathogenesis of diabetes by proposing treatment principles of supplementing collateral Oi, invigorating the spleen and nourishing Jin. Granules consist of *Ginseng, Huangjing, Atractylodes, Sophora flavescens, Ophiopogon japonicus, Radix rehmanni AE,* and *Polygonum multiflorum*. This treatment embodies the idea of "treating from the spleen" to reduce diabetes as part of the theory of collateral disease, balancing of the diet and the metabolic process to treat disease.

Basic research on Jinlida demonstrated its function protecting islet β-cell[32], anti-oxidative stress[33-34], regulating hormones related with blood glucose[35], and protecting vascular endothelial cells[36]. Additionally Jinlida can reduce insulin resistance by regulating lipid metabolism [37-38], promote skeletal muscle gene and protein expression [39-42]as well as the liver signaling pathway [43].

Based on the unique advantages of Jinlida granules in clinical practice, Chinese experts and scholars have performed a series of clinical research. Lian Fengmei et al[44] conducted a randomized controlled trial to evaluate the efficacy and safety of Jinlida Granules in combination with metformin in the treatment of type 2 diabetes. This study showed that, based on dietary control, exercise therapy and metformin intervention, Jinlida Granules could control glycosylated hemoglobin in type 2 diabetes mellitus better, at the same time it placed an advantage in regulating lipid metabolism disorder, reducing waist circumference and body weight of subjects. Tian Jiaxing et al.[45] explored the advantages of Jinlida granules in the treatment of type 2 diabetes subjects through stratification analysis analysis. The results of the trial showed that when confirming the efficacy of Jinlida granules with extensive hypoglycemic effect, it was clear that there were hypoglycemic advantages to subjects with poor glycemic control, male, elderly, long-term, and with lean body shape. In addition, the study also found that Jinlida granules had an effect reducing the insulin resistance in subjects with hyperinsulinemia and promoting insulin secretion. In November 2014, Ning Guang et al.[46] conducted a randomized, controlled clinical study of subjects with type 2 diabetes mellitus complicated with dyslipidemia who had poor dietary glycemic control and exercise. The results of these clinical research studies has elevated Jinlida granule treatment into the "Guidelines for the Clinical Practice of Diabetes in Traditional Chinese Medicine 2016" and "Guidelines for the Prevention and Treatment of Type 2 Diabetes in China 2017".

The previous clinical subjects in studies of Jinlida granules are mainly type 2 diabetes subjects. A clinical trial on pre-diabetes subjects has not been performed. By examining the effect of multiple targets (improving insulin sensitivity index and insulin resistance, reducing glycosylated hemoglobin, regulating abnormal lipid metabolism, reducing body mass index) in Jinlida granules, our research team will implement a randomized, double-blind, placebo-controlled and multi-center clinical trial to investigate the efficacy and safety of intervention with Jinlida granules in metabolic syndrome with abnormal glucose metabolism.

3 Trial objectives

To evaluate the effectiveness of Jinlida in delaying or inhibiting the development of diabetes mellitus in metabolic syndrome subjects with impaired glucose tolerance. To provide clinical evidence for high-risk factors requiring intervention to prevent arteriosclerosis and inhibit the development of the cardiovascular pathology.

4 Study design

This is a randomized, double-blind, placebo-controlled, multicenter clinical trial.

4.1 Sample size calculation

According to 'Fengmei et al. (2014)', after 1 year of follow-up, the average annual incidence of diabetes in the placebo group was 29.32%. 'Gao Y et al. (2013)' demonstrated an cumulative incidence of diabetes in placebo group after 3 years of follow-up was 43.86%.

Our hypothesis assumes that the incidence of diabetes in the control group would be 40.0% after 2 years of intervention and the HR would be expected to be 0.75------the study drug is predicted to reduce the risk by 25%------employing α=0.05, β=0.20. The study group and the control group will be allocated in a 1:1 ratio. The study period will last 4 years, including 2 years of medication treatment and 2 years of follow-up. PASS16 software will be employed to calculate the sample size, each group should contain 395 subjects. Considering a 10% the violation rate and issues of inter-center case allocation, a total of 880 cases and evaluation of number of endpoint events 209:171 will be required to reach statistical significance.

4.2 Randomization

Subjects who sign an informed consent form will receive a screening number consisting of two parts. The first two digits are the center number, and the last three digits are sequentially added according to the order in which the subjects are screened. For example, at the 01 center. The first, second, and third patient screening numbers are 01001, 01002, and 01003, and so on.

Subjects will be randomized using a central randomized system. When the subjects meet the enrollment criteria, the clinical study coordinator will be required to enter the subject's general information (name, age, gender) into an interactive voice/network response system (IWRS/IVRS). There they will obtain a random number and a drug number for a corresponding visit. Drugs will be distributed randomized subjects according to the drug number.

5 Subjects

5.1 Prediabetes diagnostic criteria

Reference "China Type 2 Diabetes Prevention Guide (2017 Edition)"

Sugar metabolism classification	Intravenous plasma glucose (mmol/L)	
	Fasting blood glucose	Two hr after glucose load
Impaired fasting glucose (IGT)	≥6.1，<7.0	<7.8

Impaired glucose tolerance (IFG)	<7.0	≥7.8，<11.1

Note: IFG and IGT are collectively referred to as impaired glucose regulation and are diagnosed as pre-diabetes.

5.2 Diagnostic criteria for type 2 diabetes

Reference "China Type 2 Diabetes Prevention Guide (2017 Edition)"

Diagnostic criteria	Intravenous plasma glucose (mmol/L)
(1) Typical symptoms of diabetes (polydipsia, polyuria, polyphagia, idiopathic weight loss) plus random blood glucose level	≥11.1
(2) Fasting blood glucose	≥7.0
(3) Subjects two hours after glucose load without typical diabetes symptoms should be confirmed in other day.	≥11.1

Note: Fasting status is defined as intake no calories for at least 8 hours; random blood glucose is defined as testing without consideration of the last meal time. Blood glucose taken at any time of the day cannot be used to diagnose as abnormal fasting blood glucose.

5.3 Diagnostic criteria for metabolic syndrome

Refer to "China 2 Diabetes Prevention and Treatment Guide (2017 Edition)", International Diabetes Federation (IDF) 2005 for the diagnostic criteria of metabolic syndrome:

Indispensable indicators:

Abdominal obesity (central obesity): waist circumference in male ≥ 90cm, female ≥ 85cm;

Other indicators: At least two of the following four indicators can diagnose metabolic syndrome

1. Hyperglycemia: Fasting blood glucose ≥ 6.1 mmol/L or 2h after glucose load ≥ 7.8 mmol / L and / or have been diagnosed as diabetes and received treatments.
2. Hypertension: blood pressure ≥ 130/85 mmHg and/or have been confirmed to be hypertension and received related treatments..
3. Fasting TG ≥ 1.70 mmol/L.
4. Fasting HDL-C < 1.04 mmol/L.

5.4 Inclusion criteria

1 Subjects aged between 18 and 70 years old;

2 After the run-in period, subjects should meet the diagnostic criteria for metabolic syndrome;

3 After the run-in period, subjects should meet the diagnostic criteria for impaired glucose tolerance;

4 Subjects who have signed the informed consent form.

4.1 Exclusion criteria

1. Subjects who have used hypoglycemic drugs in the past 3 months;
2. Subjects with type 1 or type 2 diabetes (T2D), gestational diabetes, secondary diabetes and other special types of diabetes;
3. Hyperthyroidism or hypothyroidism caused by endocrine disorders;
4. Uncontrollable hypertension/hypotension: systolic blood pressure ≥200mmHg or diastolic blood pressure ≥110mmHg; or systolic blood pressure ≤90mmHg or diastolic blood pressure ≤60mmHg;
5. Comorbidities such as various acute infections, or severe infections, severe anemia and neutropenia;
6. Major disease comorbidities, such as active or untreated malignant tumors, or clinical remission of malignant tumors less than 5 years ago;
7. Natural heart disease, such as congenital heart disease, rheumatic heart disease, hypertrophic or dilated cardiomyopathy, NYHA cardiac function classification > III level;
8. Subjects who had any of the following conditions within the past 6 months: coronary intervention (eg: CABG or PTCA), stroke (including ischemic and hemorrhagic stroke);
9. Severe liver and kidney dysfunction (ALT three times greater than the upper limit of the normal and creatinine greater than 132 μmol / l);
10. After the run-in period, fasting TG is less than 5.6mmol/L;
11. Pregnant or lactating women, and women of childbearing age who have not taken effective contraceptive measures;
12. Participation in any other clinical trials;
13. For any reason, the investigator may consider a subjects inappropriate for participation in this study.

4.2 Elimination criteria

Subjects that have been enrolled but meet one of the following criteria should be excluded:

1. False acceptance;

2. During the trial, other Chinese medicines similar to the ingredients of the study drug are taken at the same time;

3. Subjects with poor compliance (medication compliance rate is less than 80% or greater than 120%);

4. The principle investigator will consider it unsuitable for the patient to continue to participate in the trial;

Note: A reason should be noted for elimination of any subject. Their original medical records should be recorded but no statistical analysis is necessary at this point.

4.3 Drop-out criteria

All subjects who sign the informed consent and are screened for entry into the trial, are allowed to drop-out at any time or for any reason, as long as they do not complete the study are called drop-out cases. The investigator also has the right to withdraw the patient from the trial in the event of disease, adverse event, violation of the study protocol, management reasons, or other reasons. Unnecessary patient withdrawal should be avoided as much as possible, and active measures should be taken to complete the final follow-up as much as possible in order to assess study drug efficacy and safety.

If a patient decides to withdraw, the investigator should contact the patient or his responsible relatives by telephone or personal interview to confirm the reason for withdrawal. The investigator collect any remaining drug from the patient at the time of withdrawal, completing the final assessment as well as explaining the reason for withdrawal. If the reason for withdrawal was an adverse event, the primary event should be recorded in the case report form (CRF).

Reasons for drop-out in general: adverse events, lack of efficacy, violation of the protocol (including poor compliance), loss to follow-up (including patient withdrawal), suspension by the sponsor and others.

4.4 Termination criteria for subjects

1. An allergic reaction that is clearly associated with the study drug;
2. Adverse symptoms or signs and abnormal examination results that are clearly related to intake of the study drug;
3. Women who develop pregnancy during the study;
4. Subjects requests to withdraw from the study;
5. Signs of severe or frequent hypoglycemia: hypoglycemic events that require help from others (eg: increased physical activity or no meals), or weekly hypoglycemia events (plasma or hand-pricked blood glucose measurements≤70mg/dL [≤3.9mmol/L] any episode with or without symptoms) occurred more than 3 times;
6. According to the results of OGTT screening, the patient was diagnosed as type 2 diabetes, and retesting one week later, OGTT was still diagnosed type 2 diabetic;
7. Cardiovascular or cerebrovascular events that occur during the study including: acute myocardial infarction, stroke, hospitalization for heart failure, etc.

4.5 Termination criteria for overall-trial

1) Serious safety problems occur during the trial;
2) Curative effect of the drug is too poor and it is ineffective to continue the trial;
3) During the course of research, it is found that the clinical protocol has major errors or significant deviations occur in the implementation so that it is difficult to evaluate the effectiveness of drug;
4) The sponsor requests to suspend the study (such as funding or management issues, etc.);
5) The administrative department cancels the trial.

5 Intervention

5.1 Treatment

Grouping

Study group: general lifestyle intervention + Jinlida granules 1 bag/time, 3 times / day; drug consumed with boiled warm water;

Control group: general lifestyle intervention + placebo 1 bag/time, 3 times / day; drug consumed with boiled warm water;

The research drugs are recommended to taken after meals. If the patient has an intolerable adverse event that considered as relevant to the study drug based on the investigator's consideration, the patient should discontinue the medication.

5.2 General lifestyle intervention

The Guidelines for the prevention and treatment of type 2 diabetes (2017 edition) suggest that subjects with abnormal glucose metabolism should reduce their risk of diabetes through diet control and exercise. Regular follow-up and psychosocial support to ensure that lifestyle changes can persist for a long time are needed; as well as checking blood glucose regularly and paying close attention to other cardiovascular risk factors (such as smoking, hypertension, dyslipidemia, etc.) including giving appropriate intervention measures. The primary objectives of the intervention are as follows:

1. Maintaining a healthy weight: the weight loss goal for overweight/obese subjects is 5% to 10% of weight loss in 3-6 months. A lean patient should maintain an ideal body weight through a reasonable nutrition plan.
2. Providing a balanced diet to meet the physical demand for micronutrients.
3. Achieving and maintaining ideal blood glucose levels as well as reducing HbA1c levels.
4. Reducing risk factors for cardiovascular disease including control of dyslipidemia and high blood pressure.

5.3 Dietary intervention

(A) Energy

1. Pre-diabetes or diabetic subjects should receive a nutrition plan focusing on individualized energy balance to achieve and maintain an ideal body weight. Nutritional requirements should be formulated according to the subject’s situation.
2. People with diabetes who are overweight or obese should be recommended to lose weight. However, it is not recommended that subjects with type 2 diabetes receive very low-energy (<800kcal/d) nutritional therapy long-term.

(B) Adipose

1. The energy provided by fat in any diet should account for 20-30% of total energy consumption.
2. The intake of saturated fatty acids should not exceed 7% of total diet energy, and the intake of trans fatty acids should be reduced. Mono-unsaturated fatty acids are a good source of dietary fatty acids, and the energy supply ratio of total fat intake should be 10-20%. Polyunsaturated fatty acid intake should not exceed 10% of total energy intake, and the proportion of n-3 fatty acid-rich should be appropriately increased in a subject’s diet.
3. Refer to the Chinese Dietary Guidelines (2016) for recommendations about controlling excessive intake of cholesterol in the diet.

(C) Carbohydrates

1. The energy provided by carbohydrates in the diet should account for 50-65% of total energy intake. Additionally, the quantity and quality of carbohydrates is a vital part of glycemic control.
2. Low glycemic index food is beneficial for blood glucose control, but blood glucose load should also be considered.
3. It is safe for diabetics to take moderate amounts of sugar alcohols and non-nutritive sweeteners. Excessive sucrose decomposition of fructose or excessive addition of fructose may lead to an increase in TG synthesis, which is not conducive to fat metabolism.
4. Regularly rationed meals while trying to keep the carbohydrates evenly distributed.
5. Control the intake of additive sugar; do not allow consumption of sugary drinks.

(D) Protein

1. In diabetic subjects with normal renal function, protein intake can account for 15-20% of the energy supply ratio, ensuring that the proportion of high-quality protein exceeds one-third of energy supply.
2. Recommended protein intake of about $0.8g\cdot kg^{-1}\cdot d^{-1}$. Excessive protein intake (such as $>1.3g\cdot kg^{-1}\cdot d^{-1}$) is related with proteinuria and kidney decreased function, increasing risk of cardiovascular and death. Protein intake below $0.8g\cdot kg^{-1}\cdot d^{-1}$ does not delay the progression of diabetic nephropathy, and protein intake in dialysis subjects has been increased appropriately. The source of protein should be based on high-quality animal protein, supplemented with compound α-keto acid preparation if necessary.
3. Within the recommended intake range, simply increasing protein consumption does not cause

an increase in blood glucose, but may tend to increase the insulin secretion response.

(E) Drinking alcohol

1. Drinking alcohol is not recommended for diabetic subjects. If the subject does drink alcohol, this should be included in the total energy calculation.
2. The amount of alcohol of a female's daily drinking should be no more than 15g, and the male intake should not exceed 25g (15g alcohol is equivalent to 350ml beer, 150ml wine or 45ml of distilled liquor), no more than two times a week.
3. Please be aware hypoglycemia is induced by alcohol consumption so drinking on an empty stomach should be avoided.

(F) Dietary fiber

Beans, fiber-rich cereals (≥5g fiber per serving), fruits, vegetables and whole grain foods are good sources of dietary fiber. Increasing dietary fiber intake is good for health. It is recommended that diabetic subjects achieve a daily recommended intake of dietary fiber: 10-14 g/1000 kcal.

(G) Sodium

1. The intake of salt should be limited to 6g per day, and daily intake of sodium should not exceed 2000mg. Subjects with hypertension should strictly limit such intake.
2. It is also necessary to limit the intake of seasonings or foods with high sodium content, such as processed foods such as monosodium glutamate, soy sauce, sauces, preserved products, and salt soaking.

(H) Micronutrients

Diabetic subjects are prone to a lack of B vitamins, vitamin C, vitamin D and chromium, zinc, selenium, magnesium, iron, manganese and other micronutrients, which can be supplemented according to nutritional assessment results. Long-term use of metformin should prevent vitamin B12 deficiency. Long-term, robust supplementation of vitamin E, vitamin C, carotene and other anti-oxidant preparations is not recommended as the long-term safety of such a practice remains to be verified.

(J) Dietary patterns

Different dietary intervention models require individualized dietary treatments to be designed under the guidance of a professional, combined with the patient's metabolic goals and personal preferences (eg: customs, culture, religion, health concepts, economic status, etc.). A reasonable diet model refers to a diversified dietary pattern based on cereals and high dietary fiber intake, with low salt, sugar and fat intake. A reasonable diet cannot reduce the risk of type 2 diabetes by 20%. Meta-analysis of six large cohort studies and 21 randomized controlled trials demonstrate that the intake of 48-80 g whole grains per day can have a 26% reduction in risk of developing type 2 diabetes. In addition, a meta-analysis of 430,000 people from several countries showed that high meat intake may increase the risk of type 2 diabetes by 20%. Therefore, it is recommended to control the intake of meat. Simultaneous monitoring of changes in blood lipids, renal function, and nutritional status is required.

5.4 Exercise intervention

The following principles should be followed when subjects exercise:
1. Exercise therapy should be performed under the guidance of a physician. Necessary assessments should be performed before exercise, especially in medical assessment of cardiopulmonary function and motor function (eg; exercise load test, etc.).
2. Adult type 2 diabetes subjects should exercise at least 150 minutes per week (eg, 5 days per week, 30 minutes each time) with moderate intensity (50% to 70% maximum heart rate with rapid heartbeat and breathing but not shortness) aerobic exercise. It found that even short-term physical exercise (such as 10min) or a cumulative average 30min/d, is also beneficial.
3. Medium-intensity sports include: brisk walking, Tai chi, cycling, table tennis, badminton and golf. Greater intensity exercises include fast-paced dance, aerobics, jogging, swimming, cycling uphill, football and basketball.
4. If there is no contraindication, it is best to do resistance exercises 2~3 times every week (two exercise intervals ≥48h) to improve muscle strength and endurance. This exercise should include the main muscle groups such as the upper limbs, lower limbs, and trunk, at medium training intensity. A combination of resistance exercise and aerobic exercise contributes to a greater degree of metabolic improvement.
5. The exercise program should be adapted to the patient's age, condition and physical endurance, and should be regularly evaluated to adjust the exercise plan in a timely manner. Maintaining a sports diary may help to improve athletic compliance. Blood glucose monitoring should be strengthened before and after exercise. Subjects should be advised to temporarily adjust their diet and medication regimen so as to avoid hypoglycemia when exercising or exercising intensely.
6. Developing healthy living habits and cultivating active lifestyles, such as increasing daily physical activity, reducing sedentary time, and integrating beneficial sports into daily life, should be a patient goals.
7. Fasting blood glucose >16.7mmol / L, repeated hypoglycemia or blood sugar fluctuations, acute metabolic complications such as DKA, acute infection, proliferative retinopathy, severe kidney disease, severe cardiovascular and cerebrovascular disease (unstable angina, severe arrhythmia, transient ischemic attack, etc.) ban exercise. Exercise can be gradually restored when the disease is stable.

5.5 Smoking cessation

All subjects who smoke should be recommended to stop smoking or to stop using tobacco products. Additionally, steps should be taken to reduce passive smoking. If necessary, assess the subject's smoking status and the degree of nicotine dependence and provide counseling options or a smoking cessation hotline.

5.6 Intervention method and follow-up

The control group will receive orally administered Jinlida granule’s placebo on the basis of general lifestyle intervention. The study group will receive the Jinlida granules orally with general lifestyle interventions.

Additional general lifestyle interventions will consist of collective education, card issuance, and family members' permission to intervene.

The design and implementation of the entire study procedure will be performed under the guidance of a reference and metabolic syndrome or endocrinologist. The performance of the general lifestyle intervention will be also assessed in the study medical records.

5.7 Combined medication and combined treatment

During the study period,

1) The use of oral and/or injectable drugs is prohibited as well as health supplements that have hypoglycemic effects.
2) Lipid-lowering and antihypertensive drugs are allowed, but they must be recorded in the study medical record and described in detail.
3) Use weight-loss drugs or metabolic surgery are prohibited.
4) If a pharmacological treatment for other systemic diseases is to be applied, it must be recorded in the research medical record in detail and explained.
5) All concurrently employed drugs and other treatments should be documented in detail in the study medical record and described in detail.

5.8 Research drug

Research drug name: Jinlida granules

Ingredients: *ginseng, Huangjing, wheat bran, Atractylodes lancea, Sophora flavescens, Ophiopogon japonicus, Rehmannia glutinosa, Polygonum multiflorum, Hawthorn, Poria, Perrin, Coptis, AnemarrhenAE, Astragalus, Salvia miltiorrhiza, P. chinensis, Litchi nucleus.*

Traits: This product consists of light yellow to brownish yellow particles, its smell is slightly fragrant and its taste is slightly bitter.

Specifications: 9g per bag

Production batch number: A1810001, A1810002

Approval number: National Drug Standard z20050845

Production unit: Shijiazhuang Yiling Pharmaceutical Co., Ltd.

The drug is provided free of charge by Shijiazhuang Yiling Pharmaceutical Co., Ltd. and a qualified drug test report is also issued.

5.8.1 Preparation, packaging and labeling

Jinlida and Jinlida placebo will be provided in the form of granules with the printed label “Clinical Research Drugs”, and nine bags of either substance in each small box.

Placebo: Jinlida Granule Simulator (placebo and tested drug are identical in color, size, packaging, label, shape of the contents, etc.)

(Basis of placebo design: treatment and application of medication in this study are consistent with Helsinki Declaration and its Supplementary note, “the prevention, diagnosis, or treatment of a study is used for a milder condition, and the patient's acceptance of a placebo does not present any increasing risk of serious or irreversible injury.”

Considering that any drug may have a placebo effect, only after removing the effect the placebo can it be determined whether the test drug is effective. Therefore, a placebo control will be administered to a set of subjects in order to objectively and accurately evaluate Jinlida as treatment for metabolic disease.

All research drugs and simulants have been provided free of charge by Shijiazhuang Yiling Pharmaceutical Co., Ltd. Issued with a qualified drug test report.

5.8.2 Preservation method

The study drug should be stored in a safe and temperature-controlled room (room temperature should not exceed 20 °C) while paying attention to moisture exposure. Each research center must be designated a person to be in charge of research drug management.

5.8.3 Drug dispensing and recycling

A random number generated by central randomized system will be used . After the subject's run-in period and after meeting the inclusion criteria, the investigator will log the subject into the central random system by entering the subject's initials, gender, age, stratification factors, etc. and will recieve a random number. The subject's random number will also be entered into the "random number" column of the study medical record. At each follow-up, the system will show the pack number of the drug that the subject should issue. The drug number for each dose is different. The sender must dispense the drug in accordance with the drug package number displayed on the system.

5.8.4 Drug inventory

At each visit, subjects should return all remaining medications. Then the investigator should count the remaining medications and record them in a timely fashion to determine compliance, in the medical record.

Compliance with medication = total amount of medication used ÷ total amount of medication required by the protocol × 100%.

After the study, the research drug administrator is responsible for returning the remaining drugs to the drug providing unit: Shijiazhuang Yiling Pharmaceutical Co., Ltd.

5.8.5 Drug management

This study will use central randomization. According to the estimated progress of the trial, the appropriate amount of drugs will be delivered to the research centers. During the study period, drugs will be distributed according to the actual study progress and distributed to the subjects by the drug management personnel of the research center. The distribution process of the drug should be recorded accordingly. In order to ensure the timely supply of drugs, the central random system will pre-set the amount of drugs allocated. Once a stock becomes insufficient, monitors will deliver the drugs in time according to the quantity indicated by the system. After confirming that the delivered drug arrives at the research unit, the system will account for drug to the applicant. The drug is administered in the research centers with same operation. After the trial, the test drug administrator is responsible for returning the remaining drugs to the sponsor or destroying them according to procedures. The test drug counter will be locked and stored at room temperature.

6 Study program

The day of a subject's enrollment is defined as day 0.

Screening period (Day -37 days ~ -1 month)

Subjects will voluntarily participate in this clinical study, in conjunction with the treatment and related testing provisions of this program, and voluntarily accept general lifestyle interventions.

Run-in period (-1month to day 0)

Subjects who are fully eligible for inclusion after the one-month run-in period and who do not meet the exclusion criteria will be randomly assigned.

Randomized treatment period (1st to 48th months)

The investigator provides the patient with dietary and exercise guidance. Subjects will be asked to adjust their lifestyle habits according to the investigator's guidelines and take the study drug orally. The study drug is recommended taking about 30 minutes after meals a day. The medication should not be taken on the morning of the visit. If the patient does not take the medicine one day, the dose of the next day may not exceed the daily dose. The study does not allow for dose adjustment. If the patient has an intolerable adverse event that is considered relevant to the study drug based on the investigator's consideration, the patient should discontinue the study medication.

Tracking visits

SubjectsSubjects will be followed up at the end of the randomized treatment period to assess relevant secondary outcomes.

7 Treatment period

The run-in period is 1 month and the treatment period is 24 to 48 months.

8 Compliance evaluation

Subjects will be evaluated for treatment adherence by recording the complete distribution and recovery of the drug. The actual dose of the drug should be within the range of 80% to 120% of the applied drug amount. It should be determined that drug compliance is in accordance with the trial requirements.

9 Efficacy outcome

9.1 Primary efficacy indicator

The incidence of diabetes, diabetes is defined as OGTT results that describe as type 2 diabetes, with a weekly review of subjects whose OGTT is still diagnosed with type 2 diabetes, which mean diabetes developed.

The time of diagnosis at the first OGTT is the time of diabetes onset. If the diabetes does not occur, this case is defined as censored.

9.2 Secondary efficacy indicators

1. Changes in the number of components of the metabolic syndrome
2. Changes in single outcome (waist circumference, blood pressure, TG, HDL-C) in metabolic syndrome
3. Carotid intima-media thickness (IMT)
4. Fasting insulin
5. Ankle brachial index
6. Glycated hemoglobin (HbA1c)
7. Endothelial function test: Serum NO, ET-1
8. C-reactive protein
9. Urinary microalbumin to creatinine ratio (ACR)

9.3 Safety indicators

Routine blood and urine examination, liver and kidney function, electrocardiogram, adverse events, physical examination, etc.

10 Research process

10.1 Screening period (-37 days - 1 month)

Subjects who are diagnosed with impaired glucose tolerance syndrome will be given a one-month general lifestyle intervention.

This visit should include the following:

（1） Signing informed consent

（2） Physical examination

（3） Detection of fasting blood glucose and two hr postprandial blood glucose by capillaries

（4） Guidance for subjects on general lifestyle interventions

10.2 Run-in period (-1 month - day 0)

Conduct a one-month general lifestyle intervention.

10.3 Entry period

(The day of random enrollment is defined as day 0, and the following content is completed within 7 days before random enrollment)

- Collect general information: name, gender, date of birth, height, weight, waist circumference, etc.
- Related tests (including: OGTT test, 4 blood lipids, glycosylated hemoglobin, fasting insulin, blood routine, urine routine, liver and kidney function, 12-lead ECG, C-reactive protein, carotid ultrasound, urinary microalbumin, serum NO, ET-1, thyroid function, NYHA classification, brachial index)
- Vital signs
- Record combined disease and combined medication
- Review inclusion criteria and exclusion criteria
- IWRS will be randomly grouped; research drugs distributed, and drug release records completed.
- Schedule the subject's next follow-up date and inform the patient of the appointment. Additionally, they should be made aware that they should not take the study drug and maintain an empty stomach on that day.

Special instructions:

1. Liver and kidney function testing mentioned in this program contains the following items: glutamate aminotransferase, aspartate aminotransferase, alkaline phosphatase, total bilirubin, γ-glutamate peptidase, urea nitrogen or urea, creatinine, uric acid.

2. Carotid ultrasound must measure the bilateral carotid intima-media thickness (IMT); include the largest IMT record to the study medical record, and record the corresponding position and its fixed measurement during follow-up.

10.4 Randomized treatment period 1

(1, 2, 4, 5, 7, 8, 10, 11, 13, 14, 16, 17, 19, 20, 22, 23, 25, 26, 28, 29, 31, 32, 34, 35, 37, 38, 40, 41, 43, 44, 46, and 47 months) following items will be completed at the end of each month.

- Adverse event record
- Record combined medication
- Vital signs (including weight, waist circumference)
- Laboratory tests (including: detection of fasting blood glucose through capillaries and 2 h postprandial blood glucose)
- The subject should be informed about their next follow-up appointment date. Further, they should understand that they should not take the study drug on the follow-up day and maintain an empty stomach before the appointment.

Remarks: If diabetes is diagnosed based on capillary blood glucose test results, fasting blood glucose is detected by venous blood or OGTT test will be performed. If diabetes is still diagnosed, the subject will be asked to complete the OGTT test again one week later. If diabetes is still diagnosed, the subject must withdraw from the study and completes all of the same items as when enrolled, and if not diagnosed with diabetes, continues this clinical study.

10.5 Randomized treatment period 2

(The following items will be completed at the end of the 3rd, 9th, 15th, 21st, 27th, 33th, 39th, and 45th months)

- Adverse event record
- Record combined medication
- Collection of remaining drugs and evaluate medication compliance
- Vital signs (including weight, waist circumference)
- Laboratory tests (including: OGTT test)
- Distribute research drugs and complete drug release records
- Schedule the subject's next follow-up date and inform them of the follow-up day so the subject does not take the study drug and maintains an empty stomach.

Remarks: If diagnosed with diabetes, a one-week (3 boxes) study drug should be given for follow-up, and the subject should be asked to perform the OGTT test again one week later. If the patient is still diagnosed with diabetes, the subject must withdraw from the study. All the same examination items as those at the time of enrollment will be completed, and if the diagnosis does not stand, the remaining study medications for this follow-up will be issued.

10.6 Randomized treatment period 3

(The following items will be completed at the end of the sixth, 18th, 30th, and 42nd months)

- Adverse event record
- Record combined medication
- Recover remaining drugs and evaluate medication compliance
- Vital signs (including weight, waist circumference)
- Related tests (including:OGTT test, 4 lipids, glycosylated hemoglobin, fasting insulin, blood routine, urine routine, liver and kidney function, 12-lead ECG, c-reactive protein, urine microalbumin)
- Distribute research drugs and complete drug release records
- Schedule the subject's next follow-up date and inform the subject not to take the study drug and keep an empty stomach on the day of follow-up.

Remarks: If diagnosed with diabetes, a one-week (3 boxes) study drug should be given for this follow-up, and the subject should be asked to perform the OGTT test again one week later. If the patient is still diagnosed with diabetes, the subject must withdraw from the study. All the same examination items as those at the time of enrollment will be completed. If the diagnosis does not stand, the remaining study medications for this follow-up will be issued.

10.7 Randomized treatment period 4

(The following items were completed at the end of the 12th, 24th, and 36th months)

- Adverse event record
- Record combined medication
- Recover remaining drugs and evaluate medication compliance
- Vital signs (including weight, waist circumference)
- Related tests (including: OGTT test, four lipids, glycosylated hemoglobin, fasting insulin, blood routine, urine routine, liver and kidney function, 12-lead ECG, C-reactive protein, carotid ultrasound, urinary microalbumin, serum NO, ET-1, brachial index, urine routine, liver and kidney function, 12- lead ECG, c-reactive protein, urine microalbumin)
- Distribute research drugs and complete drug release records
- Schedule the subject's next follow-up date and inform the follow-up day that subject do not take the study drug and keep an empty stomach

Remarks: If diagnosed with diabetes, a one-week (3 boxes) study drug should be given for this follow-up, and the subject should be notified to perform the OGTT test again one week later. If the patient is still diagnosed with diabetes, the subject withdraws from the study. All the same examination items as those at the time of enrollment will be completed, and if the diagnosis do not stand, the remaining study medications for this follow-up will be issued.

10.8 Last follow-up

(At the end of the 48th month or at time of event)

- Adverse event record
- Record combined medication
- Recover remaining drugs and evaluate medication compliance
- Vital signs (including weight, waist circumference)
- Related tests (OGTT test, four lipids, glycosylated hemoglobin, fasting insulin, blood routine, urine routine, liver and kidney function, 12-lead ECG, C-reactive protein, carotid ultrasound, urinary microalbumin, serum NO, ET-1, brachial index)
- Complete the study completion summary form

10.9 Tracking visits: (required)*

* If the subject has persistent adverse events at the end of treatment or clinically significant laboratory and vital signs abnormalities, researchers are required to conduct visits to obtain results of follow-up measurements. The follow-up observation by the researcher according to the specific situation can also be carried out by telephone.

11 The meaning of an endpoint event and the judgment charter

During the study, subjects diagnosed with type 2 diabetes according to the OGTT results, and one week later, the subjects whose OGTT are still diagnosed as type 2 diabetes are considered to have an endpoint event.

This trial has an Endpoint Events Review Committee composed of two members and a chairman. The chairman of the committee will convene the meeting in due course according to the cumulative number of events, or the sponsor investigator may propose to hold a meeting. Chaired by the chairman of the committee, all members of the committee must attend.

All the events collected will be discussed one by one, and all members will vote separately (without abstention) and adopt the principle of majority, forming a final judgment conclusion.

Within five working days after the meeting, the committee members (two members may take turns in charge) write the minutes of the meeting and the conclusions of the meeting, and submit them to the chairman of the committee as well as the person in charge of the study by mail.

12 Observation of adverse events

12.1 Definition

- Adverse events (AEs): Any adverse medical event that occurred between the time the subject signed the informed consent and the last follow-up, regardless of whether or not there was a causal relationship with the study drug.

- Significant adverse events: Any adverse events and hematology and/or other laboratory abnormalities that result in targeted medical measures (eg, withdrawal, dose reduction, and symptomatic treatment) in addition to serious adverse events.

12.2 Classification standards of adverse events

All clinical adverse events that occur in this study will be recorded on the Study Medical Records Adverse Events page of the CRF where the severity of adverse events will be graded.

The classification standards of adverse event are ranked as follows:

Level 1: Mild, no clinical symptoms or mild clinical symptoms; only clinical or laboratory abnormalities; no treatment required.

Level 2: Moderate, requiring minimal and partial or non-invasive treatment; age-appropriate activities of daily life with using tools are limited in A.eg: cooking, shopping, and telephone calls.

Level 3: Severe conditions or medically severe symptoms that not life-threatening for the time being but may result in prolonged hospitalization or hospitalization as well as those leading to disability or limitations in daily life. Self-care in daily life is defined as: bathing, dressing, undressing, eating, going to the bathroom, taking medicine, etc., not bedridden.

Level 4: Life-threatening, requiring urgent treatment.

Level 5: Death due to adverse events.

Note :

A: Instrumental activities of daily life refer to cooking, purchasing daily necessities or clothes, using telephones, and managing money.

B: Self-rational activities in daily life refer to bathing, wearing/undressing, eating, washing, taking medicine, and not being bedridden.

Attention should be paid to the severity and intensity of adverse events. Severity is used to describe intensity, not necessarily a serious adverse event (SAE). For example, a headache may be severe in intensity, but it cannot be classified as a serious adverse event unless it meets the criteria of a SAE.

12.3 Judging criteria for the relationship between adverse events and research drugs

Causal analysis of the relationship between all adverse events and the study drug will be judged into five level: affirmation, possible, possibly irrelevant, unrelated, and undecidable.

Affirmation: the occurrence of AE and the use of study drugs have a reasonable time sequence; AE is a known adverse reaction to the study drug. However, after the drug is stopped and the AE is reduced or disappears. Or the drug is repeated to use again without explained by the disease of the subject itself.

Possible: the occurrence of AE and the use of test drugs have a reasonable time sequence, AE is a known or suspected adverse reaction of the test drug, but there are other factors that may cause the

event, such as disease, combined medication, etc. After the reaction is alleviated or disappears, or the effect after drug withdrawal is unclear or lack of decisive information.

Possibly irrelevant: the occurrence of AE and the use of the test drug are in a reasonable chronological order, but the event is not a known type of adverse drug reaction and is most likely caused by pre-existing disease or other treatments.

Unrelated: A situation where there is no reasonable time sequence for the occurrence of AE and exposure to test drugs. Such events occurred before the test drug is used; it is not a known adverse drug reaction; or AE is caused by other factors, such as: subject disease, other treatments or combined medications.

Undecidable: There is no clear relationship between the time when AE appears and the time sequence of medication. Adverse events are similar to the type of reaction known to the test drug, and other drugs used may cause the same response, and there is not enough evidence.

The total number of "Affirmation", "Possible", and "Undecidable" adverse reactions of the research drug, and the incidence of adverse reactions will be calculated accordingly.

12.4 Judgment of serious adverse events

12.4.1 Definition of general serious adverse events

A serious adverse event is any clinical event that represents significant harm, contraindications, side effects. Adverse events are classified as serious adverse events when they meet one or more of the following criteria:

- Death
- Life-threatening (risk of immediate death due to the event; do not include events that would lead to death if the patient became worse)
- Requires hospitalization or extended hospital stay
- Causes permanent or significant disability, or severely affect daily living ability
- Congenital malformation or birth defects
- Important medical event

Medical events that do not result in death, danger to life, or hospitalization, should be treated with appropriate medical judgments so as not to cause harm to the patient or subject and may require medication or surgery treatment.

12.4.2 Operating procedures

(1) During drug clinical research, all types of personnel are responsible for their duties, clear responsibilities in accordance with the regulations.

(2) Standard operating procedures should be followed during all aspects of drug clinical research.

(3) A researcher should observe or follow up the various reactions in detail that occur after the drug is administered, so as to timely detect adverse events or serious adverse events, promptly treating them.

(4) In the course of drug clinical research, once the subject has an adverse reaction, regardless of whether or not there is a causal relationship with the study drug, the investigator should record and sign all adverse events in the original record. In the case of adverse drug reactions, the adverse reactions should be initially judged based on the criteria for adverse drug reactions and drug causality.

(5) For subjects with common adverse reactions to research drugs, the researcher should immediately report PI and determine the necessary diagnosis and treatment according to the condition, as well as decide whether to suspend the clinical study. All adverse events should be tracked, detailed records of treatment and results, until properly resolved or stable, if the abnormalities should be traced back to normal. The follow-up method can select hospitalization, outpatient, home visit, telephone, communication, etc. according to the severity of adverse reaction time.

(6) If a serious adverse event occurs, a doctor should immediately provide treatment and proceed according to the SOP of the first-aid, as well as ask the relevant professional department to consult. If the situation is urgent, subjects should be urgently sent to the ICU for treatment accompanied by the medical staff. At the same time, PI should be reported immediately

Measures of adverse event management

When an adverse event is discovered, the investigator should immediately report to the department head and determine the necessary diagnosis and treatment according to the condition, and decide whether to suspend clinical research observation. All adverse events should be tracked, detailed records of treatment and results, until properly resolved or stable, if the abnormalities should be followed-up to normal. The follow-up method can select hospitalization, outpatient, home visit, telephone, communication, etc. according to the severity of adverse reactions.

12.4.3 Measures of serious adverse event management

The researcher should report to the person in charge of the department and promptly relevant personnel of the research team according to the nature of the adverse event. For example, on holidays or at night, the medical staff on duty should immediately notify the hospital medical administrative staff on duty and notify the office of the drug clinical trial agency. The office of the drug clinical trial institution shall report to the medical ethics committee, research sponsor, and Shijiazhuang Yiling Pharmaceutical Co., Ltd. within 24 hours; if emergency including serious, especially fatal or other serious adverse events, the regulation departments should be reported in the fastest way (including telephone, fax, express mail, E-mail, etc.).

Unit	Contact	Contact number	Fax

(Several adverse events need to be reported to the relevant units within 24 hours)

12.5 AE/SAE ending/transition

AE/SAE can be described as: recovered/resolved, recovering/resolving, unrecovered/unresolved, recovered/resolved with sequela, dead or unknown, as follows:

Recovered/Resolved: "(Severe) Adverse Event End-Date" should be noted.

Recovering/Resolving: An event is not fully resolved, but the patient is in recovery. Requires follow-up.

Unrecovered/unresolved: An event is in progress.

Recovered/resolved with sequela: Only when the patient has persistent or lifelong sequela, such as blindness caused by diabetes or hemiplegia caused by stroke. The "(Severe) Adverse Event Termination Date" should be noted.

Death: "(Severe) Adverse Event Termination Date" should be noted. When an AE leads to death, the time of death should also be recorded.

Unknown: The researcher does not know the AE status, for example, if the patient is lost to follow-up.

If the AE outcome is rated as "recovering/solving", or "unrecovered/unresolved", or "unknown", the AE termination date may not be recorded temporarily.

If the AE outcome is assessed as "recovered/resolved" or "recovered/resolved with sequela", the AE termination date must be recorded.

All AE must be followed to determine the final outcome.

When the patient completes the clinical study, the investigator should follow up on the outcome of AE related or unrelated the research drug.

12.6 Abnormal laboratory results

The investigator should judge whether the abnormality of the laboratory results is clinically meaningful and give a possible explanation. Laboratory abnormalities resulting from adverse events that have been reported should also be recorded as adverse events in the adverse events table. A clinically significant laboratory test abnormality that satisfies one or more of the following conditions should be recorded as an independent diagnosis in the adverse event page of the study medical record (excluding abnormal laboratory results due to previous adverse events):

- Clinical symptoms
- Lead to research drug changes

Need to change the combination of medications and/or other treatments

13 Blind method

The drug packaging shall be prepared by the blinding personnel of the statistical unit which is the unrelated the trial. According to the drug packaging number generated by the software and the corresponding intervention group, the drug packaging number of the research drug and the control drug shall be filled in (or pasted on) the label. The second-level blind bottom will be produced during on-the-spot blinding, which is reserved in sponsor unit.

This trial will use a central randomized system to dispense medications during the follow-up period, so the medications are packaged at follow-up intervals. The random number assigned to each subject will be unique, except that the package number of the drug received during different follow-up period will differ, but the corresponding treatment plan will remain consistent. All blinded personnel must sign the blinding record and make a document for the clinical trial

The drug package number and confirmation code information will be imported into the DAS for IWRS system after blinding, and are used when applying for random numbers, dispensing drugs, and unblinding online.

13.1 Emergency letter

Each random number will be set up with an emergency letter (electronic), and the electronic emergency letter will be recorded the intervention group corresponding to the drug package number received by the center random system. Emergency letters are used for disclosure by strict authorization. Only unblinded personnel can open the electronic emergency letter, and the operation will be recorded (eg: the electronic signature of the reader, the date and reasons for the deletion).

13.2 Emergency unblinding

In the occurrence of an adverse event, an emergency unblinding may only be carried out in a situation where the study drug must be known in order to treat the patient. Once the decision is made, the investigator must record the date, time, and reason for the unblinding.

Researchers are required to log into RTSM to fill in the unblinding application, which will be reviewed by the principle investigator and then broken by the blind person. Once blinded, the case will be discontinued as a drop-out count.

13.3 Unblinding regulations

In this study, the two unblinding method can be used. When the data is entered into the database in duplicate, the review is confirmed and the final statistical plan is confirmed. The database would be locked. At this time, the first time unblinding would be carried out by the principle investigators together with the statisticians, which the blind bottom of the group corresponding to the random number (group A or group B) is revealed. After the first analysis is completed, the principle

investigator will conduct a second unblinding at the clinical research summary meeting. All unblinding procedures should be documented.

14 Data management

14.1 Fill in and transfer the case report form

The investigator should ensure that the data is correctly, completely, clearly, and timely entered into the case report form based on the original observations of the subject. The auditor shall monitor whether the research is conducted in accordance with the research plan and confirm that whether all case report forms are completed correctly and consistent with the original data. If errors and omissions are made, the researcher shall be promptly corrected. The case report form after the inspection by the auditor needs to be transmitted to the data administrator of the clinical research in time.

14.2 Data entry and modification

The data administrator should compile the data entry-program for data management. In order to ensure the accuracy of the data, two data entry personnel should independently perform double entry and proofreading.

For questions in the case report form, the data administrator will generate a question-answerform and send an inquiry to the researcher through the clinical monitor. The researcher should answer the question as soon as possible, and the data administrator will modify the data according to the researcher's answer. If necessary, a question answer form can be issued again

14.3 Blindness review and unblinding

A blinding audit is the verification and evaluation of the database data until the last medical case report form is entered into the database until the first unblinding.

When all the case report forms are double-entered and verified, the data manager will write a database check report, including the completion of the study (including the list of drop-out subjects), the inclusion/exclusion criteria check, the integrity check, and the study logic (consistency check, outlier data check, time window check, combined drug check, adverse event check, etc.).

At the blinding review meeting, the principle investigators, sponsors, monitors, data administrators, and bio-statisticians reviewed the blind state of the research process and the emergency unblinding of the research process, reviewing the issues raised in the report and making a blind audit report. The database will be locked at the same time. The locked data file can then no longer changed.

15 Statistical Analysis

After the research trial is finalized, a statistical professional is responsible for developing a statistical analysis plan in consultation with the principle investigator. The statistical analysis software (SAS® 9.2 software) will be used. The sample size calculation software PASS16 will be used

15.1 Analysis subjects

The research subjects will be divided into the following categories:

- 1. Full Analysis Set (FAS)
- Full analysis set (FAS) refers to the data set obtained from all randomized subjects with the least amount and reasonable method of eliminating subjects. Exclusions usually include violations of important inclusion criteria; subjects do not receive treatment with the test medication; no observational data is obtained after randomization.

2. Per Protocol Set (PPS)

- Per Protocol Set (PPS): A subset of the full analysis set, and these subjects are more compliant with the trial. Subjects included in the PPS generally have the following characteristics: (1) the minimum exposure of the research drug in advance-completion, that is, the compliance with the drug is 80%; (2) the data of the primary outcomes in the test are available; (3) no major violation of the protocol.

3. Security data set (Safety Set, SS)

- Safety Data Set (SS): A subject who received at least one-time treatment after randomization and has a safety assessment.

15.2 Statistical analysis method

- All statistical tests will be performed using a two-sided test, and a p-value less than or equal to 0.05 (two-sided test) will be considered to be statistically significant results (except for special instructions).
- Descriptive analysis: This data is described by the number of cases and the composition ratio. The measurement data are described by means of mean, standard deviation, maximum value and minimum value. The non-normal distribution data are described by median, 25th and 75th quantiles.
- A comparison between the two groups of general conditions will be based on outcomes. Quantitative data will be compared between groups using group t test or Wilcoxon rank sum test, classification data using chi-square test or exact probability method, grade data used Wilcoxon rank sum test or CMH test, as appropriate.

15.3 Enrollment and completion

The number of completions and completions of each center as well as list the cases of drop-out cases will be summarized. The different database sizes of each group, the distribution of each central case, the total drop-out rate, and a detailed list of termination reasons should be describable. The demographic characteristics (age, height, weight, vital signs, etc.), medical history and medication history of the subjects are described, and the age, height and weight of the two groups will be compared to measure the comparability of the two groups.

15.4 Compliance analysis

- Drug Compliance Analysis: Evaluate whether the study and control subjectssubjects took their drug on time and at the correct dosage.
- Prohibited drug compliance analysis: Evaluate whether a subject has taken medications and foods that are banned in the protocol.
- Combination drug analysis: The number subjectsof subjects taking different drug combinations should be counted and listed in detail.

15.5 Efficacy evaluation

The incidence of diabetes will be estimated by using the Kaplan-Meier method and Logrank test will be performed between the two groups. Cox proportional hazards model and the covariates between the indicated groups (the factors defined in the center or other SAP implementations) will be used to calculate the hazard ratio between treatment groups and their 95% confidence interval (both sides) as well as the Kaplan-Meier curve will be plotted.

Secondary efficacy outcomes will be selected according to the different types of data.

15.6 Safety evaluation

The subjects will be described as the normal before treatment and the abnormal cases after treatment, rating the proportion of the number of cases. Adverse events will be described by the number of occurrences, the number of cases, and the incidence of adverse events. The incidence will be tested for significance between groups. At the same time, the specific performance and extent of all adverse events in each group of cases and their relationship with drugs should be described in detail.

15.7 Interim analysis

In this study, an interim analysis will be conducted. In order to control for Class I error n the trial, the O'Brien-Fleming statistical method will be used for correction. A nominal test level of the

mid-term analysis should be 0.005, and the nominal test level of statistical analysis at the end of the test should be 0.048.

16 Quality Control

16.1 Improvement measures of observation consistency

(1) Personnel participating in the observation and collection of clinical data should have a high degree of professional knowledge and skills.

(2) Through pre-clinical training, researchers will have a full understanding and understanding of the clinical trial plan and its specific outcomes. The description of the subjective symptoms should be objective, do not induce or prompt; for the specified objective outcomes, the time and method specified in the plan should be checked. Care should be taken to observe adverse reactions and unforeseen toxic side effects and to follow up. The case record form cannot be arbitrarily altered (if it needs to be modified, it should be modified according to the required standard method).

(3) Information that is significantly deviated or outside the acceptable range shall be verified by the investigator with the necessary instructions.

(4) Each test item must indicate the unit of measurement used.

(5) Each clinical research unit needs to organize a research team. In addition to the need for a unit PI to bear the overall responsibility, it should also be designated as the general coordinator of the trial, to help the unit PI specifically coordinate the coordination of clinical and imaging examination departments, regularly checking the progress of clinical trials, carefully supervising and verifying the timely recording of information.

(6) When necessary, the responsible unit for the study will organize a clinical coordination telephone or video conference for timely check of the preliminary work, analysis of the problems identified and the clinical trial process. Then they will propose rectification plans and specific measures to ensure the smooth implementation of the subject.

(7) The clinical monitor should follow the standard operating procedures, supervising the implementation of the research program, confirming that all data records and reports are correct and complete, and all research medical records are correctly reported and consistent with the original data.

(8) The responsible unit of the research may entrust the auditor to systematically check the activities and documents related to clinical research to evaluate whether the research is carried out in accordance with the program, standard operating procedures and relevant regulations.

16.2 Laboratory quality control requirements

Each participating hospital laboratory (or laboratory) must establish standard operating procedures and quality control systems for research and measurement outcomes in strict accordance with national regulations and standards. When the main outcomes may be subjectively affected, a consistency test is required. When the test results of the central laboratories have large differences or the normal reference value ranges are different, effective measures should be taken to verify or correct them in time to avoid test deviation.

16.3 Measures to ensure subject compliance

1) It is necessary to make sure the subject understands the meaning of the test, and to urge the family to help with for drug compliance. The investigator will issue a lifestyle intervention manual for each subject to regulate their lifestyle.

2) For subjects with poor adherence, it is necessary to understand and analyze the reason for deviance and strengthen follow-up.

This study requires the relevant responsible unit to dispatch or entrust a clinical research monitor to ensure that the rights and interests of the subjects in the clinical trial are protected, the test records and reported data are accurate and complete, and testing follows the approved program and the quality of the drug clinical trial, management practices and related regulations.

17 Ethical considerations

1) In the course of clinical research, individual rights of the subjects must be fully protected and the scientific and reliability of the research must be ensured. Subjects' rights, safety, and health are of higher than concern that scientific and social interests.

2) This research trial needs to be reviewed and approved by the ethics committee at each participating institution. Any adjustment of the study protocol should be approved by the Ethics Committee during the study. Serious adverse events in the trial should be reported to the Ethics Committee in a timely manner.

3) Before each patient is enrolled in the study, the researcher will provide a complete and comprehensive presentation of the purpose, procedures, and benefits and possible risks of the study in writing (or its designated representative). Subjects should be made aware that they have the right to withdraw from the study at any time. Before selection, each subject must provide informed consent by filling out the required form. A subject may be enrolled in this clinical trial after he or she has voluntarily signed this consent form by themselves or an immediate family member. This informed consent form should be used as one of the original data for clinical trials.

18 Research progress

April-August 2018	Complete the research protocol and hold a seminar
September-December 2018	Final research trial
January to April 2019	Ethical review, research drug, material preparation
May 2019	Registration
May 2019	Start of the trial
June 2019	Screen the first subject
December 2019	Complete random enrollment of 50% of cases
December 2020	Complete random enrollment of all subjects
June 2021	Complete follow-up of all subjects
August 2021	Complete the collection and entry of all clinical research data
December 2021	Statistical analysis and data management
February 2022	Complete statistical research report

19 Data reservation

The research hospital shall maintain the original data for 5 years after the termination of the clinical study. This includes confirming that all subjects (effectively checking different records, such as study medical records and original hospital records), and all original informed consent of subjects, research medical records and detailed records of drug distribution, etc.

20 References

[1] Wang Wei, clinical observation of lifestyle interventions related to metabolic syndrome [d], Beijing: Beijing University of Chinese Medicine, 2014

[2] "Guidelines for the Prevention and Treatment of Type 2 Diabetes in China (2017 Edition)"

[3] Eckle RH,Grundy SM,ZIMMET PZ.The metabolism syn-drome [J].Lancet,2005,365(9468):1415-1428

[4] Wu Guixian, Wu Zhaosu, Liu Jing, et al. Cohort study of central cerebrovascular morbidity in subjects with metabolic syndrome in 11 provinces [j], Chinese Journal of Epidemiology, 2003, 24(7): 551-553

[5] International Diabetes Federation.The IDF consensus world-wide defintion of the metabolic syndrome [J].Diabetes Res Clin Pract,2005(67):251-257.

[6] Alberti KG,Shaw ZP.IDF Epidemiology task force consensus group,the metabolic syndrome a new worldwide definition [J].Lancet,2005,366(9491):1059-1062.

[7] Charles M. NCEP-Defined Metabolic Syndrome, Diabetes, and Prevalence of Coronary Heart Disease Among NHANES III Subjects Age 50 Years and Older[J]. DIABETES, 2003, VOL. 52(MAY):1210-1214.

[8] Yudkin JS. To: Gerstein HC, Pogue J, Mann JFE, Lonn E,Dagenais GR, McQueen M, Yusuf S, for the HOPE investigators (2005) The relationship between dysglycAEmia and cardiovascular and renal risk in diabetic and non-diabetic subjects in the HOPE study: a prospective epidemiological analysis. Diabetologia 48:1749-1755[J]. Diabetologia, 2006,49(3):611-612; author reply 613-614. DOI: 10.1007/s00125-005-0115-1.

[9] Punthakee Z, Werstuck GH, Gerstein HC. Diabetes and cardiovascular disease: explaining the relationship[J]. Rev Cardiovasc Med, 2007, 8(3):145-153.

[10] Xu Y, Bi Y, Li M, et al. Significant coronary stenosis in asymptomatic Chinese with different glycemic status[J]. Diabetes Care, 2013, 36(6):1687-1694. DOI: 10.2337/ dc12-0977.

[11] Lindström J, Louheranta A, Mannelin M, Rastas M, Salminen V et al. (2003) The Finnish Diabetes Prevention Study (DPS): Lifestyle intervention and 3-year results on diet and physical activity. Diabetes Care 26: 3230-3236.

[12] Ambady R, Chamukuttan S (2008) Early diagnosis and prevention of diabetes in developing countries. Rev Endocr Metab Disord 9: 193-201.

[13] Singleton JR, Smith AG, Russell JW, Feldman EL. Microvascular Complications of Impaired Glucose Tolerance. Diabetes. 2003 Nov 21;52(12):2867–73.

[14] Scott, M, Grundy, MD, PHD. Pre-Diabetes, Metabolic Syndrome, and Cardiovascular Risk[J]. Journal of the American College of Cardiology, 2012, 59(7): 635-43.

[15] Adam, G, Tabák, MD, PhD. Prediabetes: A high-risk state for developing diabetes[J]. Lancet, 2012, 379(9833): 2279-2290.

[16] Wenying, Yang, M, D, Juming, Lu, M, D. Prevalence of Diabetes among Men and Women in China[J]. N Engl J Med j o u r na l o f m e dic i n e, 2010, 362(12): 1090-1101.

[17] Knowler WC, Fowler SE, Hamman RF, et al. 10-year follow-up of diabetes incidence and weight loss in the Diabetes Prevention Program Outcomes Study. Lancet. 2009; 374:1677–86.

[18] Gerstein HC, Yusuf S, Bosch J, et al. Effect of rosiglitazone on the frequency of diabetes in subjects with impaired glucose tolerance or impaired fasting glucose: a randomised controlled trial. Lancet. 2006; 368:1096–105.

[19] Knowler WC, Barrett-Connor E, Fowler SE, et al. Reduction in the incidence of type 2 diabeteswith lifestyle intervention or metformin. N Engl J Med. 2002; 346:393–403.

[20] Ramachandran A, Snehalatha C, Mary S, et al. The Indian Diabetes Prevention Programme shows that lifestyle modification and metformin prevent type 2 diabetes in Asian Indian subjects with impaired glucose tolerance (IDPP-1). Diabetologia. 2006; 49:289–97.

[21] Tuomilehto J, Lindstrom J, Eriksson JG, et al. Prevention of type 2 diabetes mellitus by changes in lifestyle among subjects with impaired glucose tolerance. N Engl J Med. 2001; 344:1343–50.

[22] Yang W, Lu J, Weng J, et al. Prevalence of diabetes among men and women in China. N Engl J Med. 2010;362:1090–1101.

[23] Li G, Zhang P, Wang J, et al. The long-term effect of lifestyle interventions to prevent diabetes in the China Da Qing Diabetes Prevention Study: a 20-year follow-up study. Lancet. 2008;371:1783–1789.

[24] Ji L, Hu D, Pan C, et al. Primacy of the 3B approach to control risk factors for cardiovascular disease in type 2 diabetes subjects[J]. Am J Med, 2013, 126(10):925. .e11-22. DOI: 10.1016/j.amjmed.2013.02.035.

[25] R, K, Simmons. The metabolic syndrome: useful concept or clinical tool?[R]. Diabetologia:WHO, 2010. 600-605.

[26] Yi-Lien, Liu. Low Intensive Lifestyle Modification in Young Adults With Metabolic Syndrome A Community-Based Interventional Study in Taiwan[J]. Medicine Volume 94, 2015, (22): 1-8.

[27] Kirby MG: Cardiovascular disease prevention, screening and early detection in Turkey. Cardiology 2010, 115:294–296.

[28] Hoekstra T, Beulens JW, van der Schouw YT: Cardiovascular disease prevention in women: impact of dietary interventions. Maturitas 2009, 63:20–27.

[29] Sarrafzadegan N, Kelishadi R, Esmaillzadeh A, Mohammadifard N, Rabiei K,Roohafza H, Azadbakht L, Bahonar A, Sadri G, Amani A: Do lifestyle interventions work in developing countries? Findings from the Isfahan Healthy Heart Program in the Islamic Republic of Iran. Bull World Health Organ 2009, 87:39–50.

[30] Wadden TA, Foster GD: Behavioral treatment of obesity. Am Soc Clin Nutr 2005, 82(Suppl):230–235.

[31] Jin Lei. Research on TCM Syndrome of Metabolic Syndrome [d]. Beijing: China Academy of Chinese Medical Sciences, 2013. 1-119.

[32] Shi Yuli, Wu Ying, Song Yuping, et al. Protective effect of Jinlida Granule on islet β cells in diabetic rats[J].Journal of Second Military Medical University,2012,33(4):385-389.

[33] Yixuan Liu,An Song,Shasha Zang,et al.Jinlida reduces insulin resistance and ameliorates liver oxidative stress in high-fat fed rats[J].Journal of Ethnopharmacology,2015,(162):244-252.

[34] Liu Yuxuan, Yan Shasha, Song Guangyao, et al. Effects of oxidative stress and JNK, p38MAPK pathway in liver of rats fed with high fat diet[J]. Zhonghua Journal of Traditional Chinese Medicine, 2015, 30(6): 2156- 2159.

[35] Pang Jie, Gao Huailin, Wang Hongtao, et al. Study on the intervention effect of Jinlida on blood glucose regulating hormone and islet cell function in diabetic rats [j]. Chinese Journal of Basic Medicine in Traditional Chinese Medicine, 2014, 20 (5): 605-648.

[36] Pang Jie, Liang Junqing, Wang Zhixin, et al. Intervention and mechanism of Jinlida combined with Tongxinluo on high glucose-induced islet microvascular endothelial cell injury. Chinese Pharmacological Bulletin [j], 2015, 31(3): 430 -434.

[37] Duan Liyuan, Zhao Jing, Liu Yuxuan, et al. Effects of Jinlida on serum leptin and adiponectin in rats with insulin resistance induced by high fat diet [j]. Chinese Journal of Medicine, 2014, 26 (12): 8 -11.

[38] Dingkun Wang,Min Tian,Yuan Qi,et al.Jinlida granule inhibits palmitic acid induced - intracellular lipid accumulation and enhances autophagy in NIT-1 pancreatic β cells through AMPK activation[J].Journal of Ethnopharmacology,2015,(161)99-107.

[39] Shasha Zang,An Song,Yixuan Liu,et al.Chinese medicine Jinlida（JLD）ameliorates high-fat-diet induced insulin resistance in rats by reducing lipid accumulation in skeletal muscle [J].Int J Clin Exp Med,2015,8(3):4620-4634.

[40] Jin Xin, Zhang Huixin, Cui Wenwen, et al. Effects of Jinlida on MAPK signaling pathway in high fat-induced insulin resistance ApoE-/- mice[J].中国新药杂志,2015,24(14):1648-1653 .

[41] Jin Xin, Zhang Yanfen, et al. Effects of Jinlida on cholesterol-related genes in skeletal muscle of insulin-resistant ApoE-/- mice induced by high fat[J]. Chinese Journal of Pharmacology, 2014, 30(11) :1600-1604.

[42] Jin Xin, Zhang Huixin, Zhang Yanfen, et al. Effects of Jinlida on high-fat-induced insulin resistance in bone auricular triglyceride-related enzymes in ApoE-/- mice[J].Chinese Journal of Experimental Animals,2015,23(1) :69-74.

[43] Liu Yuxuan, Pei Shasha, Song Guangyao, et al. Effects of Jinlida on pi3k/akt signaling pathway in liver of insulin resistant rats [j]. Chinese Journal of Experimental Traditional Chinese Medicine, 2015, 21(12): 72-76.

[44] Fengmei Lian,Jiaxing Tian, Xinyan Chen, et al. The Efficacy and Safety of Chinese Herbal Medicine Jinlida as Add-On Medication in Type 2 Diabetes Subjects Ineffectively Managed by Metformin Monotherapy: A Double-Blind, Randomized, Placebo-Controlled, Multi-center Trial [J]. PLOS One, 2015, 10: e0130550.

[45] Jinlida in type 2 diabetes subjects based on stratification: Results of subgroup analysis from a 12-week trial[J].Journal of Diabetes, 2017, (5):1-9.

[46] Guang Ning.Study on the efficacy and safety of Jinlida in subjects with inadequately controlled type-2 diabetes and dyslipidemia under life style intervention (ENJOY LIFE Study)[J].Journal of Diabetes, 2015 (7): 268～269.

Appendix 1: Standardized operating procedures for blood pressure measurement

The specific methods and requirements are as follows:

1) Choose a mercury column sphygmomanometer that meets the measurement criteria, or an electronic sphygmomanometer that has been validated (BHS and AMI, ESH).

2) Use a properly sized air cuff that covers at least 80% of the upper arm. For most adults, the arm circumference is 25cm-35cm, and the standard size cuff with a balloon length of 22cm-26cm and a width of 12cm can be used (the current specifications of the domestic commercial mercury column sphygmomanometer: length 22cm, width 12cm). Large size airbag cuffs should be used for fat or large arms: children should use a small size airbag cuff.

3) Before measuring blood pressure, the subject should sit at least for 5 minutes, and smoking or drinking coffee for 30 minutes is not allowed to empty the bladder.

4) The subject should sit, preferably on a chair, bare their upper arm, with the upper arm and heart at the same level. If peripheral vascular disease is suspected, the left and right upper arm blood pressure should be measured at the first visit, and the highest reading side is usually measured later. Upper arm blood pressure. In special cases, the patient can take a lying position or a standing position. Elderly, diabetic subjects and those with orthostatic hypotension should be tested for standing blood pressure. The standing blood pressure should be measured 1 minute and 5 minutes after the lying position is changed to the standing position.

5) Attach the cuff to the upper arm of the subject, and the lower edge of the cuff should be 2.5 cm above the elbow. Place the stethoscope probe in the radial artery beat.

6) When using a mercury column sphygmomanometer to measure pressure, the cuff is rapidly inflated, and after the pressure in the balloon reaches the radial artery pulsation disappears, it is increased by 30 mHg, and then slowly deflated at a constant rate (2-6 ml Hg/sec). If the heart rate is slow, the rate of deflation should be slower. After obtaining a diastolic pressure reading, quickly deflate to zero.

7) Listen carefully to the Korotkoff sound during deflation, and observe the vertical height of the convex phase of the mercury column in the first phase (first tone) and the vth phase (disappearance) of the Korotkoff sound. The contraction and compression readings take the Koi's tone phase i, and the diastolic pressure reading takes the Korotkoff sound v phase. Children under 12 years of age, pregnant women, severe anemia, hyperthyroidism, aortic regurgitation, and Korotkoff sounds do not disappear. Kobe's tone i-phase (accent) is diastolic.

8) The blood pressure measurement unit is millimeters of mercury (mmHg) for clinical use. The conversion relationship between millimeter mercury and kilopascal (kPa) is indicated in the official publication in China as 1mmHg= 0.133 kPa.

9) The measurement should be repeated 1-2 minutes apart and an average of two readings should be taken. If the two readings of systolic or diastolic pressure differ by more than 5 mmHg, measure again and take the average of three readings.

10) When reading blood pressure values by using a mercury column sphygmomanometer, the last value can only be 0, 2, 4, 6, or 8; 1, 3, 5, 7, and 9 cannot be recorded, and care should be taken to avoid the last digit preference.

Appendix 2: Common Physical Fitness outcomes

1. Body mass index (BMI) = weight / height^2 (kg / m^2)
2. Waist circumference: the subject should be measured in a vertical standing position, and their feet separated by 25-30 cm, so that their weight is evenly distributed and their breathing is smooth. A soft ruler with no elasticity and a minimum scale of 1 mm should be placed on the right side of the middle line and the upper front. The midpoint of the line connecting the lower edge of the 12th rib (usually the thinnest part of the waist) is horizontally wrapped around the abdomen for a week, and is pressed against the skin for measurement. The measured value is accurate to 0.1 cm.
3. Hip circumference: the horizontal circumference of the body measured at the most raised part of the buttocks.
4. Waist-to-hip ratio (WHR): waist/hip circumference (CM).

Research flow chart

Research phase	Run-in period	Random enrollment	Treatment period													
Time	-1 month	-7-0 days	1m ± 7 days	2M±7	3M±7	4m ± 7 days	5M±7	6M±7	7m ± 7 days	8M± 7	9M±7	10m ± 7 days	11M± 7	12M±7	Until the 48th month#	When an end event occurs
**Basic Information**																
Informed consent form	●															
General information and medical history		●														
Vital signs	●	●	●	●	●	●	●	●	●	●	●	●	●	●	●	●
**treatment**																
Inclusion/exclusion criteria	●	●														
Assign random number		●														
Distributing research drugs		●			●			●			●			●	※	
Record combined medication		●			●			●			●			●	※	●
Drug recovery					●			●			●			●	※	●
**Safety indicators**																
Blood/urine routine		●						●						●	※	●
Liver and kidney function		●						●						●	※	●
12-lead ECG		●						●						●	※	●
Serum NO, E-1		●												●	※	●
Thyroid function		●														
NYHA classification		●														
Adverse event assessment	●	●	●	●	●	●	●	●	●	●	●	●	●	●	●	●
**Efficacy index**																
Fasting blood glucose (capillary)	●	●	●	●		●	●		●	●		●	●		※	
Two hours postprandial blood glucose (capillary)	●	●	●	●		●	●		●	●		●	●		※	
OGTT test		●	◎	◎	●	◎	◎	●	◎	◎	●	◎	◎	●	※	●
Four blood lipids		●						●						●	※	●
Weight, BMI, waist circumference	●	●	●	●	●	●	●	●	●	●	●	●	●	●	●	●
Fasting insulin		●						●						●	※	●
G lycated hemoglobin		●						●						●	※	●
C-reactive protein		●						●						●	※	●
Urinary microalbumin		●						●						●	※	●
Carotid ultrasound		●												●	※	●
Brachial index		●												●	※	●

#: 13th to 48th month of follow-up, please continue the first 12 months of the visit; ◎: If the blood glucose measured by the capillaries is diagnosed as diabetes, please perform the OGTT test.※: Please check the first to 12 months of follow-up at the end of the 13th to 48th month.

1

Statistical Analysis Plan (SAP)

Intervention study of Jinlida on abnormal glucose metabolism in metabolic syndrome (JLD-FOCUS): Rationale and design of a randomized, double-blind, placebo-controlled, multicenter clinical trial

Statistical Analysis Plan

Principal Investigator: Zhen-hua Jia, Prof.

Version No.: V1.0

Version Date: 2023-02-15

Confidentiality Statement:
All relevant study information is confidential and can be neither copied nor disclosed to anyone or any institution except the study personnel, ethics committee, or relevant regulatory departments of the study institution involved in this clinical study.

Table of Contents

STATISTICAL ANALYSIS PLAN (SAP) **1**

1. INTRODUCTION **3**

1.1. Study Title 3
1.2. Study Objectives 3
1.3. Study Design 3
1.4. Sample Size 3
1.5. Randomization and Blinding 4
1.6. Study Subject 5
1.6.1. Diagnostic Criteria for Prediabetes: *5*
1.6.2. Diagnostic Criteria for Type 2 Diabetes: *5*
1.6.3. Diagnostic Criteria for Metabolic Syndrome: *6*
1.7. Inclusion and Exclusion Criteria 7
1.8. Dosage Regimen 8
1.9. Study Drug 8

2. ENDPOINTS **9**

2.1. Efficacy Endpoints 9
2.1.1. Primary Endpoint: *9*
2.1.2. Secondary Endpoints: *9*
2.2. Safety Endpoints 10

3. ANALYSIS SET **10**

3.1. Statistical Analysis Set 10

4. STATISTICAL ANALYSIS METHOD **11**

4.1. General Statistical Considerations 11
4.2. Missing Data Treatment 12
4.3. Enrollment Analysis 12
4.4. Demographic and Baseline Characteristics 13
4.5. Evaluation of Medication Compliance 13
4.6. Efficacy Analysis 14
4.6.1. Primary Efficacy Endpoint *14*
4.6.2. Secondary Efficacy Endpoint *14*
4.7. Data Carryover Rules 18
4.8. Subgroup Analysis 18
4.9. Safety Analysis 20

1. Introduction

1.1. Study Title

Intervention Study of Jinlida on Impaired Glucose Tolerance in Metabolic Syndrome.

1.2. Study Objectives

To evaluate the effect of Jinlida in delaying or reducing the progression of impaired glucose tolerance to diabetes in patients with metabolic syndrome and to provide clinical evidence for intervention of high-risk factors in preventing arterial sclerosis development and inhibiting the progression of the cardiovascular event chain.

1.3. Study Design

This study is a randomized, double-blind, placebo-controlled, multicenter clinical trial.

1.4. Sample Size

According to "*Chinese Herbal Medicine Tianqi Reduces Progression from Impaired Glucose Tolerance to Diabetes: A Double-Blind, Randomized, Placebo-Controlled, Multicenter Trial[M]*" (J Clin Endocrinol Metab: 99（2）, 2014, 648-655), after 1 year follow-up, the mean diabetes incidence rate in placebo group is 29.23% each year. Besides, "*Clinical research of traditional Chinese medical intervention on impaired glucose tolerance*" (Am J Chin Med,2013,41(1):21-32) shows that after 3 years follow-up, the accumulated diabetes incidence rate in placebo group is 43.86%.

In a 2-year intervention study, it is anticipated that the control group will have a diabetes incidence rate of 40.0% estimated hazard ratio (HR) is 0.75, which indicates a 25% reduction in the risk of diabetes in the intervention group, with a power of 80% (2-sided α =0.05 and β = 0.20), the estimated sample size is 790, 395 within each group. The study will follow a 1:1 allocation ratio

between the intervention and control groups over a 2-year inclusion period and a 2-year follow-up, resulting in a total study duration of 4 years. Accounting for a 10% dropout rate and considering the distribution of cases across centers, 880 subjects are planned to be enrolled. The study expects to observe 209 endpoint events in the intervention group and 171 in the control group.

1.5. Randomization and Blinding

Randomization

After signing informed consent, participants will be assigned a participant screening number, which is consisted by a 2-digits center number and a 3-digits screening ordering number(e.g., 01001, 01002, 01003 for the first, second, and third participants, respectively, at Center 01).

Centralized randomization system will be utilized in this trial for randomization. The clinical research coordinator will input the eligible participant's abbreviated name into the Interactive Web/Voice Response System (IWRS/IVRS) if meeting the inclusion criteria and not meeting the exclusive criteria. The IWRS/IVRS will generate a randomization number and provide the corresponding drug code for each visit, facilitating the appropriate dispensation of the designated study medication.

Blinding

The medication packaging procedure will be done by unblinded-statisticians that independent from the trial. Medication packaging numbers, generated by software, are assigned to specific treatment groups, and subsequently recorded or affixed onto labels for both the experimental and control drugs. The secondary blind covers, created during on-site blinding activities, are securely stored at the primarily responsible team for the clinical study.

In this trial, medication dispensation is based on follow-up periods, utilizing a central randomization system. Accordingly, medications are packaged following the specific follow-up cycles. Each participant is assigned a unique randomization number, ensuring distinction between packaging numbers across different follow-up periods, while the treatment assignments associated with these packaging numbers remain consistent. The process of medication blinding is meticulously

documented as blind records, endorsed by all personnel involved in the blinding process, and retained as integral components of the trial documentation. Upon completion of the blinding process, the medication packaging numbers, and corresponding confirmation code information are integrated into the DAS for IWRS system. These data serve for randomization, medication dispensation, and online unblinding procedures.

Two-times unblinding method will be applied in this study. The principal investigator, statistician, and data administrator conduct the level one unblinding on the locked data after the blind review meeting, and the group code (A/B) of each randomizing number will be released for statistical analysis. The level two unblinding will be conducted after statistical analysis, which unblinds the group code and releases the exact group name of each subject.

1.6. Study Subject

1.6.1. Diagnostic Criteria for Prediabetes:

Reference：

《Guidelines for the Prevention and Treatment of Type 2 Diabetes in China (2017 Edition)》

glycometabolism characterization	Venous Plasma Glucose Concentration（mmol/L）	
	While fasting	2h after an oral glucose load
Impaired Fasting Glucose（IFG）	≥6.1，<7.0	<7.8
Impaired Glucose Tolerance（IGT）	<7.0	≥7.8，<11.1

Note：IFG and IGT are collectively referred to as "impaired glucose regulation" or "prediabetes."

1.6.2. Diagnostic Criteria for Type 2 Diabetes:

Reference：

《Guidelines for the Prevention and Treatment of Type 2 Diabetes in China (2017 Edition)》

Diagnose Criteria	Venous Plasma Glucose Concentration（mmol/L）

(1) Classic symptoms of diabetes (polydipsia, polyuria, polyphagia, unexplained weight loss) combined with random blood glucose or	≥11.1
(2) Fasting blood glucose or	≥7.0
(3) 2-hour postprandial blood glucose in individuals without classic symptoms of diabetes should be retested on another day for confirmation.	≥11.1

Note：The fasting state refers to abstaining from calorie intake for at least 8 hours. Random blood glucose refers to blood glucose measured at any time of the day without considering the time of the last meal, and it cannot be used to diagnose fasting blood glucose abnormalities.

1.6.3. Diagnostic Criteria for Metabolic Syndrome:

Referring to the《Guidelines for the Prevention and Treatment of Type 2 Diabetes in China (2017 Edition)》and the International Diabetes Federation (IDF) 2005 Metabolic Syndrome Diagnostic Criteria：

Essential Indicators：

Abdominal obesity (also known as central obesity): Waist circumference ≥90cm for males, ≥85cm for females.

Other indicators: Diagnosis of metabolic syndrome can be made if at least 2 of the following four criteria are met:

(1) High blood glucose: Fasting blood glucose ≥6.1mmol/L or 2-hour postprandial blood glucose ≥7.8mmol/L, and/or diagnosed with diabetes and receiving treatment.

(2) High blood pressure: Blood pressure ≥130/85mmHg, and/or diagnosed with hypertension and receiving treatment.

(3) Fasting triglycerides (TG) ≥1.70mmol/L.

(4) Fasting HDL-C <1.04mmol/L.

1.7. Inclusion and Exclusion Criteria

Inclusion Criteria:

1. Age between 18 to 70
2. Individuals who meet the diagnostic criteria for metabolic syndrome after the screening period.
3. Individuals who meet the diagnostic criteria for impaired glucose tolerance after the screening period.
4. Informed consent form has been signed.

Exclusion Criteria:

1. Individuals who have been continuously using antidiabetic medications for the past 3 months.
2. Type 1 diabetes, type 2 diabetes (T2DM), gestational diabetes, secondary diabetes, and other specific types of diabetes.
3. Hyperthyroidism or hypothyroidism caused by endocrine disorders.
4. Difficult-to-control hypertension/hypotension: systolic blood pressure (SBP) ≥200mmHg or diastolic blood pressure (DBP) ≥110mmHg; or SBP ≤90mmHg or DBP ≤60mmHg.
5. Concurrent acute infections of various types or severe infections, severe anemia, or neutropenia.
6. Presence of other significant diseases such as active or untreated malignancies, or clinical remission of malignancy for less than 5 years.
7. Structural heart diseases, such as congenital heart disease, rheumatic heart disease, hypertrophic or dilated cardiomyopathy, NYHA functional class > III.
8. History of coronary artery intervention (e.g., CABG or PTCA), stroke (including ischemic and hemorrhagic stroke) within the past 6 months.
9. Severe liver or kidney dysfunction (ALT > 3 times the upper limit of normal condition, creatinine > 132μmol/L).
10. Fasting triglycerides (TG) > 5.6mmol/L after the screening period.

11. Pregnant or lactating women, and women of childbearing who age not practicing effective contraception.
12. Participation in any other clinical trials.
13. Any other reason deemed inappropriate for participation in this study by the investigator.

1.8. Dosage Regimen

JLD group: Standard lifestyle intervention + JLD 9g (1 bag), 3 times/day, dissolve in hot water and take orally.

Placebo Group: Standard lifestyle intervention + placebo 9g (1 bag), 3 times/day, dissolve in hot water and take orally.

During treatment, oral and/or injectable medications and supplements with blood glucose-lowering effects should be avoided.

The study drug is recommended to be taken after meals. If patients experience intolerable adverse events related to the study drug, according to the investigator's assessment, the patient should discontinue the treatment with the study drug.

1.9. Study Drug

Name: Jinlida Granules

Ingredients: Ginseng, Polygonati Rhizoma, Stir-fried Atractylodes Macrocephala with Wheat Bran(麦麸炒苍术), Sophora Flavescens, Ophiopogon Root, Rehmannia, Radix Polygonum Multiflorum(制何首乌), Cornus Officinalis, Poria, Eupatorii Herba, Coptis Chinensis, Anemarrhena, Epimedium Sagittatum（炙淫羊藿）, Salvia Miltiorrhiza, Puerariae Thomsonii Radix, Litchi Seed, Lycii Cortex.

Characteristics: This product is light yellow to brownish-yellow granules; it has a slight aroma and a slightly bitter taste.

Specification: Each bag contains 9g.

Batch Numbers: A1810001, A1810002

Approval Number: approval number Z20050845 （国药准字）

Manufacturer: Shijiazhuang Yiling Pharmaceutical Co, Ltd.

The investigational drug is provided free of charge by Shijiazhuang Yiling Pharmaceutical Co, Ltd. and comes with a qualified drug inspection report.

2. Endpoints

2.1. Efficacy Endpoints

2.1.1. Primary Endpoint:

The primary endpoint is the incidence rate of diabetes which is defined as the occurrence of a participant being diagnosed with type 2 diabetes based on the OGTT (Oral Glucose Tolerance Test) results, and the diagnosis is still determined as type 2 diabetes on a follow-up OGTT one week later. The date of the first confirmed OGTT diagnosis of diabetes will be considered the diabetes onset date. If diabetes does not occur by the end of the study, it will be regarded as a censoring event.

2.1.2. Secondary Endpoints:

- (1) Proportions of components of metabolic syndrome
- (2) Changes in individual indicators of metabolic syndrome, including waist circumference, fasting blood glucose,2-hour postprandial blood glucose,blood pressure, four blood lipids, and HDL-C.
- (3) Changes of Carotid intima-media thickness (IMT).
- (4) Changes of Fasting insulin levels.
- (5) Changes of Ankle-brachial index.
- (6) Changes of Glycated hemoglobin (HbA1c) levels.
- (7) Changes of C-reactive protein (CRP) levels.
- (8) Changes of Homeostatic Model Assessment of Insulin Resistance (HOMA-IR)，including all the subjects and subgroup analysis(baseline HOMA-IR⩾2.69 and baseline HOMA-IR＜2.69)

- (9) Changes of Homeostatic Model Assessment of Insulin Sensitivity (HOMA-IS)
- (10) Changes of Homeostatic Model Assessment of Beta-cell Function (HOMA-β (%))
- (11) Changes of Urinary microalbumin
- (12) Changes of Triglyceride-glucose index (TyG index)
- (13) Remission rate (waist circumference, blood pressure, BMI, fasting blood glucose, 2-hour postprandial blood glucose, insulin, glycated hemoglobin, triglycerides)

2.2. Safety Endpoints

Adverse events assessment: From the time of the participant's signing of the informed consent form and inclusion in the study until the last follow-up, any adverse medical events that occur, regardless of their causal relationship with the study drug, are considered adverse events. Among them, adverse events determined to be "definitely related," "possibly related," or "indeterminate" to study drug are considered adverse drug reactions (ADRs), and the incidence rate of ADRs is calculated.

3. Analysis set

3.1. Statistical Analysis Set

Full analysis set (FAS): FAS refers to a dataset obtained by excluding certain participants from the overall randomized subjects using minimal and reasonable criteria. The exclusions typically include individuals who violated important inclusion criteria, did not receive the investigational treatment, or had no observed data after randomization.

Per-Protocol set (PPS): PPS is a subset of FAS that includes subjects with better adherence. Subjects will be included in PPS if meet the following criteria:

1) Complete the pre-set minimum exposure dosage of the research drug (at least 80% compliance)

2) No missing values on the primary endpoint.

3) No major protocol deviation.

Safety set (SS): SS includes all subjects enrolled in the study who have received at least one dose of medication and have at least one safety evaluation.

The efficacy analysis will be based on FAS and PPS. The safety analysis will be based on SS.

4. Statistical Analysis Method

4.1. General Statistical Considerations

Statisticians are responsible for developing a statistical analysis plan (SAP) with the principal investigator after the protocol is confirmed. Statistical analysis will be performed with SAS 9.4 (or higher version; SAS Institute, Cary, NC). The sample size is calculated using PASS13 software (NCSS, Kaysville, UT).

A 2-tailed $P<0.05$ will be considered statistically significant in all statistical tests. (If no other specification)

The number of subjects, mean, median, standard deviation, the first quartile (Q1), the third quartile, minimum, and maximum will be included in the summary statistics of continuous variables. The number of subjects, frequency and percentages will be included in summary statistics of the categorical variables.

Continuous variables will be summarized with mean and SD and compared using paired t-test or Wilcoxon rank test, as appropriate. Categorical variables will be summarized with frequencies and percentages and compared using chi-square test or Fisher exact test, as appropriate. Ranked variables will be compared using Wilcoxon rank test or CMH test.

Survival analysis methods (log-rank test and Kaplan-Meier method) will be used to describe the incidence rates of diabetes in the two groups separately. The log-rank test will be employed to compare the incidence rates and occurrence times of diabetes between the two groups. In addition, the Cox proportional hazards model will be used to evaluate the influence of baseline of patient risk factors on the risk of developing diabetes.

Mixed-Effects Model Repeated Measures (MMRM) was used to analyze the changes in

repeated measurement indicators, such as waist circumference, fasting blood glucose,2-hour postprandial blood glucose, glycated hemoglobin, triglycerides, HDL-C and so on, from baseline to 36 months after treatment. The model included measurements of indicators measured on baseline, the 6th month, 12th month, 18th month, 24th month, 30th month and the 36th month as the dependent variable, and the baseline level of the such measurement indicators and treatment were included as independent variables, considering the interaction between time and treatment. The model estimated whether differences in changes in such measurement indicators over time were statistically significant after adjustment for baseline at a two-sided 0.05 level and whether differences between groups were statistically significant between the JLD and placebo groups.

4.2. Missing Data Treatment

In this study, the primary endpoint of diabetes incidence rate within four years will be analyzed using survival analysis methods. FAS and PPS will include the participants who drop out or are lost to follow-up before completing the 4-year follow-up period. Non-endpoint events will be defined as censoring events, and the censoring date will be set as the last follow-up date. No carry-forward or multiple imputations will be conducted for missing data.

Missing data of secondary endpoints or safety endpoints will not be imputed.

The following rules will be applied regarding the duration calculation when only partial dates are known. If the start date (the date of the first OGTT diagnosis of diabetes) is partially missing, with only the day missing, it will be imputed as the first day of the corresponding month. If the end date is partially missing, with only the day missing, it will be imputed as the last day of the corresponding month. If a date's day and month are missing, the date will be treated as missing.

4.3. Enrollment Analysis

Enrollment and Participants Distribution: summarize the number of enrollments and dropouts and the number of subjects who completed the trial of each center and list dropout subjects. The dataset size between groups, subjects' disposition between centers, dropout rate, and termination reasons will be listed in detail, and the flowchart will be plotted.

Dropout Analysis: include subjects who drop out due to personal intention or meeting the dropout criteria. Dropout rate and dropouts due to adverse events between groups will be compared by the chi-square test.

Study Duration: summarize the total number of participants enrolled in the study, the number of participants who completed the 1-year follow-up, and the number of participants who completed the 2-year follow-up. Additionally, the reasons for participant dropout at each annual interval and the corresponding number of dropouts for each group can be summarized.

Descriptive statistics can be used to summarize the study duration in terms of years and days. Numeric characteristics of variables can be analyzed using appropriate statistical tests to compare the groups.

4.4. Demographic and Baseline Characteristics

Demographic and baseline characteristics (age, gender, height, BMI, vital signs, etc.), medical history, etc. of the subjects will be summarized. T-test or Wilcoxon rank-sum test will be used in baseline comparison of continuous variables such as age, height, and BMI between the two groups. The chi-square test or Fisher's exact test will be used in the baseline comparison of categorical variables such as gender, and medical history.

4.5. Evaluation of Medication Compliance

Medication adherence analysis: Evaluate whether the patients in the control and study groups adhere to the study drug by taking it on time and in the prescribed dosage.

Prohibited medication adherence analysis: Assess whether patients have taken prohibited medications or foods according to the treatment.

Concomitant medication analysis: Record the number of patients in each group taking concomitant medications and provide a detailed list.

4.6. Efficacy Analysis

4.6.1. Primary Efficacy Endpoint

The incidence rate of diabetes will be estimated using the Kaplan-Meier method, and a Logrank test will be conducted to compare the incidence rates between the two groups. The Cox proportional hazards model will be utilized, incorporating the covariates (centers, age and sex), to calculate the hazard ratio (HR) between two groups with 95% CI (two-sided). Kaplan-Meier curves will be plotted.

If diabetes occurs, it will be defined as the endpoint event; diabetes occurrence time = diabetes date - randomization date + 1, while the unit is the number of days.

If participants are lost to follow-up, withdraw, or cannot complete the scheduled follow-ups for other reasons, it will be considered censoring; censoring time = last follow-up date - randomization date + 1, while the unit is the number of days.

If participants die during the trial and cannot complete the scheduled follow-ups, it will also be considered censoring, and the censoring time = death date – randomization date + 1.

If participants complete the scheduled follow-ups as per the protocol and diabetes has not occurred, it will be considered censoring, and the censoring time = last follow-up date - randomization date + 1.

In addition to calculating the overall incidence of diabetes, the cumulative incidence of diabetes in the first, second and third years will also be calculated

4.6.2. Secondary Efficacy Endpoint

(1) Proportions of components of metabolic syndrome

Descriptive analysis will be conducted to summarize the occurrence and incidence rate of components of metabolic syndrome in the study group and control group using qualitative data principles，as well as the proportion of 5, 4, and 3 components of metabolic syndrome. The incidence rates between the two groups will be compared using the chi-square test or Fisher's exact probability test. For quantitative variables, the mean, standard deviation, median, minimum value, maximum

value, lower quartile (Q1), and upper quartile (Q3) for components of metabolic syndrome (fasting blood glucose (mmol/L) and 2-hour postprandial blood glucose (mmol/L)) will be calculated. The scores of each dimension will be compared between the two groups using the t-test or Wilcoxon rank-sum test.

Frequency of statistical analysis: Every 6 months.

(2) Changes in individual indicators of metabolic syndrome, including waist circumference, fasting blood glucose,2-hour postprandial blood glucose, blood pressure, four blood lipids, and HDL-C

For each follow-up, the mean, standard deviation, median, minimum value, maximum value, lower quartile (Q1), and upper quartile (Q3) of individual quantitative indicators of metabolic syndrome (waist circumference, blood pressure, fasting blood glucose, 2-hour postprandial blood glucose, 4 blood lipid measurements, HDL-C) will be calculated. T-test or Wilcoxon rank-sum test will be employed to compare the differences between the two groups. Changes in individual indicators of metabolic syndrome relative to baseline will be statistically described, and the t-test or Wilcoxon rank-sum test will be used to compare the differences between the two groups.

Frequency of statistical analysis: Every 6 months.

(3) Changes of the Carotid intima-media thickness (IMT)

The mean, standard deviation, median, minimum value, maximum value, lower quartile (Q1), and upper quartile (Q3) of carotid intima-media thickness (IMT) will be calculated for each follow-up. The t-test or Wilcoxon rank-sum test will be used to compare the differences between the two groups.

Frequency of statistical analysis: Every 12 months.

(4) Changes of the Fasting insulin levels

The mean, standard deviation, median, minimum value, maximum value, lower quartile (Q1), and upper quartile (Q3) of fasting insulin levels between the two groups will be calculated for each follow-up. The t-test or Wilcoxon rank-sum test will be used to compare the differences between the two groups.

Frequency of statistical analysis: Every 12 months.

(5) Changes of the Ankle-brachial index

The mean, standard deviation, median, minimum value, maximum value, lower quartile (Q1), and upper quartile (Q3) of ankle-brachial index between the two groups will be calculated for each follow-up. The t-test or Wilcoxon rank-sum test will be used to compare the differences between the two groups.

Frequency of statistical analysis: Every 12 months.

(6) Changes of the Glycated hemoglobin (HbA1c) levels

The mean, standard deviation, median, minimum value, maximum value, lower quartile (Q1), and upper quartile (Q3) of HbA1c levels between the two groups will be calculated for each follow-up. The t-test or Wilcoxon rank-sum test will be used to compare the differences between the two groups.

Frequency of statistical analysis: Every 12 months.

(7) Changes of the Homeostatic Model Assessment of Insulin Resistance (HOMA-IR)

The mean, standard deviation, median, minimum value, maximum value, lower quartile (Q1), and upper quartile (Q3) of HOMA-IR between the two groups will be calculated for each follow-up. The t-test or Wilcoxon rank-sum test will be used to compare the differences between the two groups.

Frequency of statistical analysis: Every 6 months.

(8) Changes of the Homeostatic Model Assessment of Insulin Sensitivity (HOMA-IS)

The mean, standard deviation, median, minimum value, maximum value, lower quartile (Q1), and upper quartile (Q3) of HOMA-IS between the two groups will be calculated for each follow-up. The t-test or Wilcoxon rank-sum test will be used to compare the differences between the two groups.

Frequency of statistical analysis: Every 6 months.

(9) Changes of the Homeostatic Model Assessment of Beta Cell Function (HOMA-β (%))

The mean, standard deviation, median, minimum value, maximum value, lower quartile (Q1), and upper quartile (Q3) of HOMA-β (%) between the two groups will be calculated for each follow-up. The t-test or Wilcoxon rank-sum test will be used to compare the differences between the

two groups.

Frequency of statistical analysis: Every 6 months.

(10) Changes of the C-reactive protein (CRP) levels

The mean, standard deviation, median, minimum value, maximum value, lower quartile (Q1), and upper quartile (Q3) of CRP levels between the two groups will be calculated for each follow-up. The t-test or Wilcoxon rank-sum test will be used to compare the differences between the two groups.

Frequency of statistical analysis: Every 12 months.

(11) Changes of the Urinary microalbumin

The mean, standard deviation, median, minimum value, maximum value, lower quartile (Q1), and upper quartile (Q3) of changes in urinary microalbumin levels between the two groups will be calculated for each follow-up. The t-test or Wilcoxon rank-sum test will be used to compare the differences between the two groups.

Frequency of statistical analysis: Every 12 months.

(12) Changes of the Triglyceride-glucose (TyG) index

The mean, standard deviation, median, minimum value, maximum value, lower quartile (Q1), and upper quartile (Q3) of changes in the TyG index between the two groups will be calculated for each follow-up. The t-test or Wilcoxon rank-sum test will be used to compare the differences between the two groups.

Frequency of statistical analysis: Every 12 months.

(13) Remission rate (waist circumference, blood pressure, BMI, fasting blood glucose, 2-hour postprandial blood glucose, insulin, glycated hemoglobin, triglycerides)

Remission rate will be estimated using the Kaplan-Meier method, and the log-rank test will be performed between the two groups. The Cox proportional hazards model, adjusting for covariates (center), will be used to calculate the hazard ratio (HR) with 95% CI (two-sided). Kaplan-Meier curves will be plotted.

Frequency of statistical analysis: Every 12 months.

4.7. Data Carryover Rules

Primary efficacy endpoints:

1. Diabetes onset time (months) = (Analysis date - Randomization date + 1) / 365.25 * 12. Analysis date: the date of diabetes onset; for participants who discontinued the study due to reasons other than diabetes onset, it is the date of discontinuation; for participants who completed the study, it is the date of the last follow-up.

Secondary efficacy endpoints:

1. Remission time is the earliest follow-up date when the participant transitions to a normal state. If a participant experiences diabetes onset or discontinuation due to other reasons, the time is recorded as the date of diabetes onset or discontinuation. If the participant completes the study, it is the date of the last follow-up.
2. For participants who developed diabetes in other secondary efficacy endpoints, the data from V49 is imputed for the last follow-up that missing result.

4.8. Subgroup Analysis

Subgroup analysis will be conducted in the study to compare the efficacy between subgroups. 21 subgroups will be included in the subgroup analysis:

- Age: 44 yr, 45–59 yr, ≥60 yr
- Sex: Male, Female
- BMI: <25, 25–30, ≥30
- Waist circumference (male <100 or female<90, male≥100 or female≥90)
- Hyperlipidemia (yes, no)
- Hypertension (yes, no)
- Fasting blood glucose (<6.1, ≥6.1)
- 2-hour postprandial blood glucose (<9.4, ≥9.4)
- Glycated hemoglobin (<6.0, ≥6.0)
- Triglyceride-glucose (TyG) index (<median, ≥median)

- Fasting triglycerides (TG) (<1.7, ≥1.7)
- Fasting high-density lipoprotein cholesterol (HDL-C) (<1.04, ≥1.04)
- Number of metabolic syndrome components (<4, ≥4)
- Number of metabolic syndrome components (3, 4, 5)
- Intima-media thickness (IMT) (<1, ≥1)
- Subgroup with abnormal glucose tolerance and abdominal obesity, combined with baseline hypertension
- Subgroup with abnormal glucose tolerance and abdominal obesity, combined with baseline fasting triglycerides (TG) ≥1.7
- Subgroup with abnormal glucose tolerance and abdominal obesity, combined with baseline fasting high-density lipoprotein cholesterol (HDL-C) <1.04
- Subgroup with abnormal glucose tolerance and abdominal obesity, combined with baseline hypertension and fasting triglycerides (TG) ≥1.7
- Subgroup with abnormal glucose tolerance and abdominal obesity, combined with baseline hypertension and fasting high-density lipoprotein cholesterol (HDL-C) <1.04
- Subgroup with abnormal glucose tolerance and abdominal obesity, combined with baseline fasting triglycerides (TG) ≥1.7 and fasting high-density lipoprotein cholesterol (HDL-C) <1.04
- Subgroup with abnormal glucose tolerance and abdominal obesity, combined with baseline hypertension, fasting triglycerides (TG) ≥1.7 and fasting high-density lipoprotein cholesterol (HDL-C) <1.04
- Subgroup with baseline impaired fasting glucose(IFG)，IFG'defined when 6.1≤fasting glucose＜7.0 and plasma glucose of 2 hours post glucose-load＜7.8

Subgroup analysis for secondary efficacy endpoints will be performed using the same methods as for the secondary efficacy analysis, with subgroups defined by the occurrence of diabetes (yes, no).

4.9. Safety Analysis

Safety analysis will be based on SS. Adverse events (AEs) and adverse drug reactions (ADRs) will be summarized by tabulating the number of AEs and ADRs, the number of subjects with AEs and ADRs, the incidences rate of AEs and serious AEs (SAEs) , the incidences rate of ADRs and serious ADRs, while listing AEs and ADRs.

Summarize AEs, SAEs, and their respective incidences rate categorized by severity. In addition, summarize the number and incidence rate for both adverse events leading to discontinuation and serious adverse events leading to death.
