# Supplementary material for: Jinlida for Diabetes Prevention in Impaired Glucose Tolerance and Multiple Metabolic Abnormalities: The FOCUS Randomized Clinical Trial
Source: JAMA Intern Med. 2024 Jun 3;184(7):727–35. doi: 10.1001/jamainternmed.2024.1190 (PMC11148787; doi:10.1001/jamainternmed.2024.1190)
Supplement: Supplement 3. — FOCUS Trial Committees and Investigators [file jamainternmed-e241190-s003.pdf]

Supplemental Online Content: Nonauthor Collaborators
*First name, last name, and suffix (if applicable) are required and will appear in PubMed.

***Group Name(s):** FOCUS Trial Committees Investigators

***First Name and Middle Initial(s)**	***Last Name**	***Suffix (eg, Jr, III)**	Academic Degrees	Institution	Location (city, state/province, country)	Role or Contribution, eg, chair, principal investigator	Group (if more than 1 Group listed in the byline) and/or Subgroup (eg, Steering Committee)
Liying	Lun		MB	Department of endocrinology, Hebei Yiling Hospital	Shijiazhuang, China	Data collection	
Sulin	Hu		MB	Department of endocrinology, Hebei Yiling Hospital	Shijiazhuang, China	Data collection	
Haipeng	Liu		MB	Department of endocrinology, Second Affiliated Hospital of Yunnan University of Chinese Medicine	Kunming, China	Data collection	
Guangyu	Cai		MB	Department of endocrinology, Second Affiliated Hospital of Yunnan University of Chinese Medicine	Kunming, China	Data collection	
Huan	Li		MB	Department of endocrinology, Second Affiliated Hospital of Yunnan University of Chinese Medicine	Kunming, China	Data collection	
Ling	Huang		MB	Department of endocrinology, Second Affiliated Hospital of Yunnan University of Chinese Medicine	Kunming, China	Data collection	
Mengling	Tian		MB	Department of endocrinology, Second Affiliated Hospital of Yunnan University of Chinese Medicine	Kunming, China	Data collection	
Jiale	Wan		MB	Department of endocrinology, Baoji Second People's Hospital	Baoji, China	Data collection	
Peizhen	Song		MB	Department of endocrinology, Baoji Second People's Hospital	Baoji, China	Data collection	
Yaqin	Zhang		MB	Department of endocrinology, Baoji Second People's Hospital	Baoji, China	Data collection	

Supplemental Online Content: Nonauthor Collaborators
*First name, last name, and suffix (if applicable) are required and will appear in PubMed.

***First Name and Middle Initial(s)**	***Last Name**	***Suffix (eg, Jr, III)**	Academic Degrees	Institution	Location (city, state/province, country)	Role or Contribution, eg, chair, principal investigator	Group (if more than 1 Group listed in the byline) and/or Subgroup (eg, Steering Committee)
Fan	Li		MB	Department of endocrinology, Baoji Second People's Hospital	Baoji, China	Data collection	
Xiaoyan	Hu		MB	Department of endocrinology, Baoji Second People's Hospital	Baoji, China	Data collection	
Jing	Xu		M.M	Department of Geriatric Internal Medicine II (Endocrinology and Metabolism) The First Affiliated Hospital of Xi'an Jiaotong University	Xi’an, China	Data collection	
Ying	Pang		MB	Department of Geriatric Internal Medicine II (Endocrinology and Metabolism) The First Affiliated Hospital of Xi'an Jiaotong University	Xi’an, China	Data collection	
Jing	Shi		M.D.	Department of Geriatric Internal Medicine II (Endocrinology and Metabolism) The First Affiliated Hospital of Xi'an Jiaotong University	Xi’an, China	Data collection	
Hongwei	Cheng		M.M	Department of endocrinology, Shaanxi Provincial Hospital of Chinese Medicine	Xi’an, China	Data collection	
Wenhong	Tian		M.M	Department of endocrinology, Shaanxi Provincial Hospital of Chinese Medicine.	Xi'an, China	Data collection	
Huiling	Wang		M.D.	Department of endocrinology, Affiliated Hospital of Shaanxi University of Trad Shenzhen Hospital itional Chinese Medicine	Xianyang, China	Data collection	

Supplemental Online Content: Nonauthor Collaborators
*First name, last name, and suffix (if applicable) are required and will appear in PubMed.

***First Name and Middle Initial(s)**	***Last Name**	***Suffix (eg, Jr, III)**	Academic Degrees	Institution	Location (city, state/province, country)	Role or Contribution, eg, chair, principal investigator	Group (if more than 1 Group listed in the byline) and/or Subgroup (eg, Steering Committee)
Gangxin	Qin		M.M	Department of endocrinology, Affiliated Hospital of Shaanxi University of Traditional Chinese Medicine	Xianyang, China	Data collection	
Qiuju	Zhao		M.M	Department of endocrinology, Affiliated Hospital of Shaanxi University of Traditional Chinese Medicine	Xianyang, China	Data collection	
Lulu	Wang		M.M	Department of endocrinology, Affiliated Hospital of Shaanxi University of Traditional Chinese Medicine	Xianyang, China	Data collection	
Junting	Luo		M.M	Department of endocrinology, Affiliated Hospital of Shaanxi University of Traditional Chinese Medicine	Xianyang, China	Data collection	
Luyv	Liu		M.M	Department of endocrinology, Affiliated Hospital of Shaanxi University of Traditional Chinese Medicine	Xianyang, China	Data collection	
Li	Zhao		M.M	Department of endocrinology, Affiliated Hospital of Shaanxi University of Traditional Chinese Medicine	Xianyang, China	Data collection	
Wenjiao	Xie		M.M	Department of endocrinology, Kunming Municipal Hospital of Traditional Chinese Medicine	Kunming, China	Data collection	
Xinlun	Cai		MB	Department of endocrinology, Kunming Municipal Hospital of Traditional Chinese Medicine	Kunming, China	Data collection	
Gaohong	Qiao		MB	Department of endocrinology, Sanmenxia Central Hospital	Sanmenxia, China	Data collection	

Supplemental Online Content: Nonauthor Collaborators
*First name, last name, and suffix (if applicable) are required and will appear in PubMed.

***First Name and Middle Initial(s)**	***Last Name**	***Suffix (eg, Jr, III)**	Academic Degrees	Institution	Location (city, state/province, country)	Role or Contribution, eg, chair, principal investigator	Group (if more than 1 Group listed in the byline) and/or Subgroup (eg, Steering Committee)
Aoran	Song		M.M	Department of endocrinology, Tongliao City Horqin District First People's Hospital	Tongliao, China	Data collection	
Jianxiu	Li		MB	Department of endocrinology, Hebei Cangzhou Hospital of Integrated Chinese and Western Medicine	Cangzhou, China	Data collection	
Wendan	Zhang		MB	Department of endocrinology, Hebei Cangzhou Hospital of Integrated Chinese and Western Medicine	Cangzhou, China	Data collection	
Qiong	Wu		M.M	Department of endocrinology, Hebei Cangzhou Hospital of Integrated Chinese and Western Medicine	Cangzhou, China	Data collection	
Yanan	Wang		M.M	Department of endocrinology, Hebei Cangzhou Hospital of Integrated Chinese and Western Medicine	Cangzhou, China	Data collection	
Qijun	Fang		M.D.	Department of Traditional Chinese Medicine, Nanjing Drum Tower Hospital	Nanjing, China	Data collection	
Lijun	Yue		M.D.	Department of Traditional Chinese Medicine, Nanjing Drum Tower Hospital	Nanjing, China	Data collection	
Yuling	Zhang		MB	Department of Traditional Chinese Medicine, Nanjing Drum Tower Hospital	Nanjing, China	Data collection	
Li	Hu		M.M	Department of endocrinology, The First Affiliated Hospital of Guangdong Pharmaceutical University	Guangzhou, China	Data collection	

Supplemental Online Content: Nonauthor Collaborators
*First name, last name, and suffix (if applicable) are required and will appear in PubMed.

***First Name and Middle Initial(s)**	***Last Name**	***Suffix (eg, Jr, III)**	Academic Degrees	Institution	Location (city, state/province, country)	Role or Contribution, eg, chair, principal investigator	Group (if more than 1 Group listed in the byline) and/or Subgroup (eg, Steering Committee)
Qin	You		MB	Department of endocrinology, The First Affiliated Hospital of Anhui University of Chinese Medicine	Anhui, China	Data collection	
Li	Wang		M.M	Department of endocrinology, Shenzhen Hospital (Futian) of Guangzhou University of Chinese Medicine	Shenzhen, China	Data collection	
Siyu	Zhao		M.M	Department of General Medicine, Baotou Clinical Medical College, Inner Mongolia Medical University	Baotou, China	Data collection	
Liwei	Zhang		M.M	Department of General Medicine, Baotou Clinical Medical College, Inner Mongolia Medical University	Baotou, China	Data collection	
Hui	Guo		M.M	Department of General Medicine, Baotou Clinical Medical College, Inner Mongolia Medical University	Baotou, China	Data collection	
Lina	Dai		M.M	Department of General Medicine, Baotou Clinical Medical College, Inner Mongolia Medical University	Baotou, China	Data collection	
Wenjuan	Niu		MB	Department of General Medicine, Baotou Clinical Medical College, Inner Mongolia Medical University	Baotou, China	Data collection	
Wei	Gao		M.M	Traditional Chinese Medicine Department,Inner Mongolia Autonomous Region People's Hospital	Huhehaote, China	Data collection	
Minzhou	Li		M.D.	Traditional Chinese Medicine Department,Inner Mongolia Autonomous Region People's Hospital	Huhehaote, China	Data collection	

Supplemental Online Content: Nonauthor Collaborators
*First name, last name, and suffix (if applicable) are required and will appear in PubMed.

***First Name and Middle Initial(s)**	***Last Name**	***Suffix (eg, Jr, III)**	Academic Degrees	Institution	Location (city, state/province, country)	Role or Contribution, eg, chair, principal investigator	Group (if more than 1 Group listed in the byline) and/or Subgroup (eg, Steering Committee)
Ruixia	Zhang		M.M	Traditional Chinese Medicine Department,Inner Mongolia Autonomous Region People's Hospital	Huhehaote, China	Data collection	
Yixuan	Liu		M.M	Traditional Chinese Medicine Department,Inner Mongolia Autonomous Region People's Hospital	Huhehaote, China	Data collection	
Jingwen	Wang		M.M	Traditional Chinese Medicine Department,Inner Mongolia Autonomous Region People's Hospital	Huhehaote, China	Data collection	
Congchao	Zhang		M.M	Traditional Chinese Medicine Department,Inner Mongolia Autonomous Region People's Hospital	Huhehaote, China	Data collection	
Siyuan	Li		M.M	Traditional Chinese Medicine Department,Inner Mongolia Autonomous Region People's Hospital	Huhehaote, China	Data collection	
Yurong	Feng		M.M	Traditional Chinese Medicine Department,Inner Mongolia Autonomous Region People's Hospital	Huhehaote, China	Data collection	
Yingran	Dai		M.M	Traditional Chinese Medicine Department,Inner Mongolia Autonomous Region People's Hospital	Huhehaote, China	Data collection	
Ren	Sa		M.M	Traditional Chinese Medicine Department,Inner Mongolia Autonomous Region People's Hospital	Huhehaote. China	Data collection	

Supplemental Online Content: Nonauthor Collaborators
*First name, last name, and suffix (if applicable) are required and will appear in PubMed.

***First Name and Middle Initial(s)**	***Last Name**	***Suffix (eg, Jr, III)**	Academic Degrees	Institution	Location (city, state/province, country)	Role or Contribution, eg, chair, principal investigator	Group (if more than 1 Group listed in the byline) and/or Subgroup (eg, Steering Committee)
Lili	Sang		M.M	Geriatrics Department , Liaocheng People's Hospital Geriatrics Department	Liaocheng. China	Data collection	
Haiyan	Wang		M.M	Geriatrics Department , Liaocheng People's Hospital Geriatrics Department	Liaocheng. China	Data collection	
Weiwei	Yin		M.M	Geriatrics Department , Liaocheng People's Hospital Geriatrics Department	Liaocheng. China	Data collection	
Hong	Zhang		MB	Geriatrics Department , Liaocheng People's Hospital Geriatrics Department	Liaocheng. China	Data collection	
Fengjun	Sun		MB	Geriatrics Department , Liaocheng People's Hospital Geriatrics Department	Liaocheng. China	Data collection	
Li	Ma		M.M	Department of Traditional Chinese medicine, The First Affiliated Hospital of Medical College of Shihezi University	Shihezi, China	Data collection	
Junyong	Yang		M.M	Department of Traditional Chinese medicine, The First Affiliated Hospital of Medical College of Shihezi University	Shihezi, China	Data collection	
Xuanmin	Zhang		M.D.	Department of Traditional Chinese medicine, The First Affiliated Hospital of Medical College of Shihezi University	Shihezi, China	Data collection	
Xia	Gan		M.M	Department of Traditional Chinese medicine, The First Affiliated Hospital of Medical College of Shihezi University	Shihezi, China	Data collection	

Supplemental Online Content: Nonauthor Collaborators
*First name, last name, and suffix (if applicable) are required and will appear in PubMed.

***First Name and Middle Initial(s)**	***Last Name**	***Suffix (eg, Jr, III)**	Academic Degrees	Institution	Location (city, state/province, country)	Role or Contribution, eg, chair, principal investigator	Group (if more than 1 Group listed in the byline) and/or Subgroup (eg, Steering Committee)
Yang	Sheng		M.M	Department of Traditional Chinese medicine, The First Affiliated Hospital of Medical College of Shihezi University	Shihezi, China	Data collection	
Yun	Zhou		MB	Department of Traditional Chinese medicine, The First Affiliated Hospital of Medical College of Shihezi University	Shihezi, China	Data collection	
Junhu	Lu		M.M	Department of Traditional Chinese medicine, The First Affiliated Hospital of Medical College of Shihezi University	Shihezi, China	Data collection	
Tong	Xu		MB	Department of Traditional Chinese medicine, The First Affiliated Hospital of Medical College of Shihezi University	Shihezi, China	Data collection	
Haoran	Li		M.M	Department of Traditional Chinese medicine, The First Affiliated Hospital of Medical College of Shihezi University	Shihezi, China	Data collection	
Zhen	Li		M.M	Department of Traditional Chinese medicine, The First Affiliated Hospital of Medical College of Shihezi University	Shihezi, China	Data collection	
Xiaoxia	Pan		MB	Department of Endocrinology, Fourth People's Hospital of Jinan City	Jinan, China	Data collection	
Li	Zhang		M.M	Department of Endocrinology, Fourth People's Hospital of Jinan City	Jinan, China	Data collection	

Supplemental Online Content: Nonauthor Collaborators
*First name, last name, and suffix (if applicable) are required and will appear in PubMed.

***First Name and Middle Initial(s)**	***Last Name**	***Suffix (eg, Jr, III)**	Academic Degrees	Institution	Location (city, state/province, country)	Role or Contribution, eg, chair, principal investigator	Group (if more than 1 Group listed in the byline) and/or Subgroup (eg, Steering Committee)
Jialin	Han		M.D.	Department of Endocrinology, Weifang Hospital of Traditional Chinese Medicine	Weifang, China	Data collection	
Acui	Yu		M.M	Department of Geriatrics, Yantai Affiliated Hospital of Binzhou Medical College	Yantai, China	Data collection	
Jingqing	Jing		M.M	Department of Geriatrics, Yantai Affiliated Hospital of Binzhou Medical College	Yantai, China	Data collection	
Li	Huang		M.M	Department of Geriatrics, Yantai Affiliated Hospital of Binzhou Medical College	Yantai, China	Data collection	
Xi	Chen		M.M	Department of Geriatrics, Yantai Affiliated Hospital of Binzhou Medical College	Yantai, China	Data collection	
Xuling	Wang		M.M	Department of endocrinologyHeilongjiang Academy Tradition of Chinese Medicine	Harbin, China	Data collection	
Jilai	Shi		M.M	Department of Endocrinology, Shandong Provincial Hospital of Traditional	Jinan, China	Data collection	
Bingyan	Wang		M.M	Department of Endocrinology, Shandong Provincial Hospital of Traditional	Jinan, China	Data collection	
Guanghui	Sun		M.M	Department of Endocrinology, Shandong Provincial Hospital of Traditional	Jinan, China	Data collection	
Kaimin	Li		M.M	Department of Endocrinology, Shandong Provincial Hospital of Traditional	Jinan, China	Data collection	

Supplemental Online Content: Nonauthor Collaborators
*First name, last name, and suffix (if applicable) are required and will appear in PubMed.

***First Name and Middle Initial(s)**	***Last Name**	***Suffix (eg, Jr, III)**	Academic Degrees	Institution	Location (city, state/province, country)	Role or Contribution, eg, chair, principal investigator	Group (if more than 1 Group listed in the byline) and/or Subgroup (eg, Steering Committee)
Tingting	Zhou		M.M	Department of endocrinology, Hebei Yiling Hospital	Shijiazhuang, China	Data collection	
Mengying	Shi		M.M	Department of Traditional Chinese medicine, Shijiazhuang 2nd Hospital	Shijiazhuang, China	Data collection	
Hongli	Liu		M.D.	Department of Traditional Chinese medicine, Shijiazhuang 2nd Hospital	Shijiazhuang, China	Data collection	
Xueling	Sun		B.S	Department of endocrinology, Tangshan Gongren Hospital	Tangshan, China	Data collection	
Hewei	Zhang		MB	Department of endocrinology, Tangshan Gongren Hospital	Tangshan, China	Data collection	
Wenhui	Hong		M.M	Department of endocrinology, Beijing Daxing District Hospital of Integrated Chinese and Western Medicine	Beijing, China	Data collection	
Nan	Huang		M.M	Department of endocrinology, Beijing Daxing District Hospital of Integrated Chinese and Western Medicine	Beijing, China	Data collection	
Xiufen	Chen		M.M	Department of endocrinology, Beijing Daxing District Hospital of Integrated Chinese and Western Medicine	Beijing, China	Data collection	
Jinxiang	Zheng		M.M	Department of endocrinology, Beijing Daxing District Hospital of Integrated Chinese and Western Medicine	Beijing, China	Data collection	
Yongjuan	Juan		M.M	Department of endocrinology, Beijing Daxing District Hospital of Integrated Chinese and Western Medicine	Beijing, China	Data collection	

Supplemental Online Content: Nonauthor Collaborators
*First name, last name, and suffix (if applicable) are required and will appear in PubMed.

***First Name and Middle Initial(s)**	***Last Name**	***Suffix (eg, Jr, III)**	Academic Degrees	Institution	Location (city, state/province, country)	Role or Contribution, eg, chair, principal investigator	Group (if more than 1 Group listed in the byline) and/or Subgroup (eg, Steering Committee)
Rongrong	Zhou		M.M	Department of endocrinology, Guang’anmen Hospital China Academy of Chinese Medical Sciences	Beijing, China	Data collection	
Huidan	Wang		B.S	Department of endocrinology, Central Hospital of Jinzhou	Jinzhou, China	Data collection	
Chunxue	Zang		M.D.	Department of endocrinology, Affiliated Hospital of Liaoning University of Traditional Chinese Medicine	Shenyang, China	Data collection	
Yiwen	Lai		M.D.	Department of endocrinology, Affiliated Hospital of Liaoning University of Traditional Chinese Medicine	Shenyang, China	Data collection	
Zedong	Peng		M.M	Department of endocrinology, Affiliated Hospital of Liaoning University of Traditional Chinese Medicine	Shenyang, China	Data collection	
Ruyu	Chen		M.M	Department of endocrinology, Affiliated Hospital of Liaoning University of Traditional Chinese Medicine	Shenyang, China	Data collection	
Xinhui	Liu		M.M	Department of endocrinology, Affiliated Hospital of Liaoning University of Traditional Chinese Medicine	Shenyang, China	Data collection	
Mengjie	Cai		M.M	Department of Endocrinology, Shuguang Hospital Affiliated to Shanghai University of Traditional Chinese Medicine	Shanghai, China	Data collection	

Supplemental Online Content: Nonauthor Collaborators
*First name, last name, and suffix (if applicable) are required and will appear in PubMed.

***First Name and Middle Initial(s)**	***Last Name**	***Suffix (eg, Jr, III)**	Academic Degrees	Institution	Location (city, state/province, country)	Role or Contribution, eg, chair, principal investigator	Group (if more than 1 Group listed in the byline) and/or Subgroup (eg, Steering Committee)
Xu	Han		M.D.	Department of Endocrinology, Shuguang Hospital Affiliated to Shanghai University of Traditional Chinese Medicine	Shanghai, China	Data collection	
