# Supplementary material for: Jinlida for Diabetes Prevention in Impaired Glucose Tolerance and Multiple Metabolic Abnormalities: The FOCUS Randomized Clinical Trial
Source: JAMA Intern Med. 2024 Jun 3;184(7):727–35. doi: 10.1001/jamainternmed.2024.1190 (PMC11148787; doi:10.1001/jamainternmed.2024.1190)
Supplement: Supplement 4. — Data Sharing Statement [file jamainternmed-e241190-s004.pdf]

Data Sharing Statement

Ji. Jinlida for Diabetes Prevention in Impaired Glucose Tolerance and Multiple Metabolic Abnormalities. *JAMA Intern Med*. Published June 03, 2024.
doi:10.1001/jamainternmed.2024.1190

Data
Data available: Yes
Data types: Deidentified participant data
How to access data: jzhjiazhenhua@163.com
When available: With publication

Supporting Documents
Document types: Statistical/analytic code, Informed consent form
How to access documents: jzhjiazhenhua@163.com
When available: With publication

Additional Information
Who can access the data: jzhjiazhenhua@163.com
Types of analyses: for a specified purpose
Mechanisms of data availability: with investigator support
